# A transdiagnostic prodrome for severe mental disorders: an electronic health record study

**DOI:** 10.1038/s41380-024-02533-5

**Published:** 2024-05-06

**Authors:** Maite Arribas, Dominic Oliver, Rashmi Patel, Daisy Kornblum, Hitesh Shetty, Stefano Damiani, Kamil Krakowski, Umberto Provenzani, Daniel Stahl, Nikolaos Koutsouleris, Philip McGuire, Paolo Fusar-Poli

**Affiliations:** 1 Early Psychosis: Interventions and Clinical-Detection (EPIC) Lab, Department of Psychosis Studies, Institute of Psychiatry, Psychology & Neuroscience, King’s College London, London, SE5 8AF UK; 2 Department of Psychiatry, University of Oxford, Oxford, OX3 7JX UK; 3 NIHR Oxford Health Biomedical Research Centre, Oxford, OX3 7JX UK; 4 OPEN Early Detection Service, Oxford Health NHS Foundation Trust, Oxford, OX3 7JX UK; 5 Department of Psychological Medicine, Institute of Psychiatry, Psychology & Neuroscience, King’s College London, London, SE5 8AF UK; 6 NIHR Maudsley Biomedical Research Centre, London, UK; 7 Department of Brain and Behavioral Sciences, University of Pavia, Pavia, Italy; 8 Department of Biostatistics and Health Informatics, Institute of Psychiatry, Psychology and Neuroscience, London, SE5 8AF UK; 9 Department of Psychosis Studies, Institute of Psychiatry, Psychology and Neuroscience, King’s College London, London, SE5 8AF UK; 10 Department of Psychiatry and Psychotherapy, Ludwig-Maximilian University, Munich, Germany; 11 Max-Planck Institute of Psychiatry, Munich, Germany; 12 Outreach and Support in South-London (OASIS) Service, South London and Maudsley (SLaM) NHS Foundation Trust, London, SE11 5DL UK

**Keywords:** Bipolar disorder, Depression, Schizophrenia

## Abstract

Effective prevention of severe mental disorders (SMD), including non-psychotic unipolar mood disorders (UMD), non-psychotic bipolar mood disorders (BMD), and psychotic disorders (PSY), rely on accurate knowledge of the duration, first presentation, time course and transdiagnosticity of their prodromal stages. Here we present a retrospective, real-world, cohort study using electronic health records, adhering to RECORD guidelines. Natural language processing algorithms were used to extract monthly occurrences of 65 prodromal features (symptoms and substance use), grouped into eight prodromal clusters. The duration, first presentation, and transdiagnosticity of the prodrome were compared between SMD groups with one-way ANOVA, Cohen’s f and d. The time course (mean occurrences) of prodromal clusters was compared between SMD groups with linear mixed-effects models. 26,975 individuals diagnosed with ICD-10 SMD were followed up for up to 12 years (UMD = 13,422; BMD = 2506; PSY = 11,047; median[IQR] age 39.8[23.7] years; 55% female; 52% white). The duration of the UMD prodrome (18[36] months) was shorter than BMD (26[35], d = 0.21) and PSY (24[38], d = 0.18). Most individuals presented with multiple first prodromal clusters, with the most common being non-specific (‘other’; 88% UMD, 85% BMD, 78% PSY). The only first prodromal cluster that showed a medium-sized difference between the three SMD groups was positive symptoms (f = 0.30). Time course analysis showed an increase in prodromal cluster occurrences approaching SMD onset. Feature occurrence across the prodromal period showed small/negligible differences between SMD groups, suggesting that most features are transdiagnostic, except for positive symptoms (e.g. paranoia, f = 0.40). Taken together, our findings show minimal differences in the duration and first presentation of the SMD prodromes as recorded in secondary mental health care. All the prodromal clusters intensified as individuals approached SMD onset, and all the prodromal features other than positive symptoms are transdiagnostic. These results support proposals to develop transdiagnostic preventive services for affective and psychotic disorders detected in secondary mental healthcare.

## Introduction

Severe mental disorders (SMD) include non-psychotic unipolar mood disorders (UMD) [1, 2], non-psychotic bipolar mood disorders (BMD) [3–6] and psychotic disorders (PSY) [7] and are characterised by high clinical, societal, familial and personal burden [8–10]. Preventive approaches for SMD can improve these outcomes but rely on accurate knowledge of the duration, first presentation, time course and specificity of prodromal features preceding their onset. The prodromal phases to SMD are typically investigated in “look-back” studies [2, 6, 11–16] (Supplementary Introduction 1) employing lengthy and complex interviews. Consequently, the findings from these studies may not directly reflect real-world clinical practice, limiting their translational relevance [3]. For example, sample sizes of look-back studies are typically small (on average ~130 UMD patients [1], ~100 BMD patients [6], ~240 PSY patients [14, 15, 17]) and the design is compromised by the effects of recall biases. Furthermore, only a limited number of prodromal symptoms are collected at a single time point, while the time course of the prodromal phase to SMD can unfold over several months and years [18] (1–84 months for UMD [2], 5–130 months for BMD [6], and 1–110 months for PSY [19]). Finally, available look-back studies are typically restricted to a single disorder, precluding the investigation of diagnostic spectrum-specific (i.e. specific to UMD, BMD or PSY) or transdiagnostic (i.e. present in at least two groups across UMD, BMD and PSY) [18, 20, 21] prodromal features.

To address these limitations, we aimed to characterise the duration and first presentation, time course and transdiagnosticity of the prodrome for SMD by employing natural language processing (NLP) algorithms in contemporaneously recorded electronic health records (EHRs) that represent real-world secondary care settings.

## Methods

This study (protocol: https://osf.io/ujcr8/) adhered to the Reporting of Studies Conducted Using Observational Routinely Collected Health Data statement (RECORD) [22] (Supplementary Table 1).

### Data source

Data were from the South London and Maudsley National Health Service Foundation Trust (SLaM). SLaM provides secondary mental healthcare across four socioeconomically diverse South London boroughs (Lambeth, Southwark, Lewisham and Croydon, 1.3 million people, Supplementary Methods 1). Incidence of psychosis in SLaM (from 58.3 to 71.9 cases per 100,000 person-years) [23, 24] is one of the highest worldwide [25]. Clinical Record Interactive Search (CRIS) was implemented in the EHR to facilitate research with full but anonymised clinical information [26] and has been extensively validated [27**–**29]. CRIS received ethical approval as an anonymised dataset for secondary analyses from Oxfordshire REC C (Ref: 23/SC/0257).

### Variables

At the index date, data were extracted from structured text on age, gender, self-reported ethnicity (UK Office of National Statistics, Supplementary Table 2), medication prescription variables (Supplementary Table 3) and ICD-10 diagnoses.

During the prodromal period, data were extracted monthly on the occurrence (yes/no) of NLP prodromal features within that month (contemporaneously recorded). Specifically, NLP algorithms were used to convert unstructured EHR information (i.e. free text) into structured quantifiable data: prodromal features [30] (see Supplementary Methods 2 for further details on NLP algorithm development and validation, and Supplementary Table 4 for the final list of NLP algorithms employed and their respective precision).

A total of 65 NLP-derived prodromal features with precision≥80% (mean=90%) were extracted monthly. These prodromal features were grouped into eight broader prodromal clusters (Supplementary Table 5): catatonic symptoms, depressive symptoms, disorganised symptoms, manic symptoms, negative symptoms, positive symptoms, substance use and other symptoms (hereby referred to as positive, negative, disorganised, manic, negative, positive, substance use and other clusters). This categorization, developed by Jackson et al. [30], is based on previous studies that utilised symptomatology factor analysis [31, 32] and is aligned with publicly available, validated NLP dictionaries [33]. This maximises reliability while simultaneously preserving real-world clinical interpretability and facilitates large-scale clinical pattern identification, crucial for evaluating treatment effectiveness and characterizing interventions, symptom profiles, and outcome-influencing factors. Therefore, each of the eight prodromal clusters is pragmatically relevant for clinical decisions in the context of secondary mental healthcare. However, as shown in Supplementary Table 5, these eight prodromal clusters are not completely independent because a few of the prodromal features (e.g. weight loss, apathy, and visual hallucinations) are included in different prodromal clusters. This overlap represents transdiagnostic phenomena spanning multiple clinical dimensions as they are observed in real-world clinical practice. Finally, to fully analyse the independent impact of each individual prodromal feature we have additionally presented a more fine-grained analysis employing prodromal features as opposed to broader prodromal clusters. When multiple first prodromal features/clusters were recorded at the same date, all of them were considered to have occurred simultaneously.

### Study design

Retrospective (up to 12 years), real-world, EHR cohort study (Supplementary Fig. 1). All individuals accessing SLaM services in the period between 1st January 2008 and 10th August 2021 and receiving a primary (i.e. not comorbid) ICD-10 diagnosis of any SMD (UMD, BMD, PSY as operationalised in Supplementary Table 6; individuals with multiple SMD diagnoses were stratified according to severity, i.e. UMD < BMD < PSY) were eligible. Therefore, if an individual receives a diagnosis of UMD and BMD simultaneously, we consider BMD to be of higher severity and they would be included in the BMD group. The index date reflected the date of the first diagnosis within an individual’s SMD group recorded in the EHR (index diagnosis, T-0mo, Supplementary Fig. 1). The antecedent date was defined by a data cut-off at six months before the index date (T-6mo), defining the antecedent period, which may overlap with the actual onset of SMD. The prodromal period (up to 12 years, T-144mo, Supplementary Fig. 1) was defined as the time from the first occurrence of prodromal features until the antecedent date in the EHR. Therefore, there were inter-individual differences in prodrome duration. Individuals with data recorded exclusively after the index date or in the antecedent period were excluded.

### Statistical analysis

We computed descriptive analyses for sociodemographic (age, gender, self-reported ethnicity) and clinical (medication prescription) variables at index date as well as the proportion (N [%]) of individuals with specific ICD-10 diagnoses in UMD, BMD and PSY. Statistical comparisons of descriptive results were not computed, in accordance with current reporting statements [34].

First, as primary outcome, we compared the duration (median [interquartile range, IQR]) of the prodromal period and the incidence of first prodromal clusters (the proportion of individuals who experienced each first prodromal cluster) between SMD groups using a one-way ANOVA model. From this model, we derived an effect size and 95% confidence intervals across all three SMD groups (Cohen’s f, three-wise “f” hereafter) and for pair-wise comparisons (Cohen’s d, pair-wise “d” hereafter). Effect sizes rather than *p*-values were primarily reported for the incidence analyses as *p*-values are confounded by the large sample size and multiple comparisons [35]. These analyses were repeated at the prodromal feature-level as supplementary. These results were complemented by violin plots (duration) and UpSet plots (incidence), which represent the most common (top 20) combinations of first prodromal clusters. These analyses were restricted to individuals for whom there was at least one NLP-derived prodromal feature. ANOVA model assumption of homoscedasticity (homogeneity of variance) was conserved, and the assumption of normality can be assumed due to the large sample size [36].

Second, we compared the annualised mean occurrences of each prodromal cluster between SMD groups for each of the 12 prodromal years using linear mixed-effects models. SMD group (with three levels: UMD, BMD, PSY) and time were included as fixed effects. Individual was included as a random intercept to account for within-subject correlations. Four models of varying complexity were fitted for each prodromal cluster: L: linear term for time; Q: linear and quadratic terms for time; L + I: interaction terms for SMD group and linear time; Q + I: interaction terms for SMD group and linear and quadratic terms for time. Random slopes were added to all models, but model convergence was not attained possibly due to a highly complex random-effects structure [37], and so were not included in the analysis. Model fit was assessed with the conditional Akaike Information Criterion (AICc) statistic which balances both model complexity and goodness of fit [38], and accounts for both random and fixed effects. Model assumptions were assessed by visual inspection of the residuals and random effect estimates [39, 40]. Line graphs and stacked line graphs were used to visualise the findings. Corrections for multiple comparisons were performed using Benjamini-Hochberg procedure with false discovery rate set at 5%.

Third, we followed the transdiagnostic research recommendations in psychiatry (TRANSD) [20, 21] to assess transdiagnosticity of the prodrome (see details in Supplementary Methods 3). The transdiagnostic construct was defined as the mean number of occurrences of each prodromal feature in the prodromal period. Comparative analyses required by TRANSD criteria were performed twofold: (i) with the above linear mixed-effects model and (ii) with three-wise and pair-wise discriminability scores. These discriminability scores estimated the degree to which the mean occurrence of a prodromal feature discriminated the three SMD groups and paired groups (BMD-UMD, PSY-UMD, PSY-BMD), respectively. These scores were based on f and d of mean occurrences of each prodromal feature in the prodromal period. The discriminability scores f and d were appraised using pre-defined thresholds [41] (positive d values indicated greater mean occurrence in the first compared to the second group, and viceversa): f < 0.1/d < 0.2 “negligible”, 0.1≤f < 0.25/0.2≤d < 0.5 “small”, 0.25≤f < 0.4/0.5≤d < 0.8 “medium”, otherwise “large”. A heat map was used to visualise the findings. Prodromal features with near-zero variance were not considered further [42].

Sensitivity analyses were conducted by repeating analyses for all core outcomes restricting the sample to: (i) individuals aged 35 or under; (ii) individuals without diagnostic spectra-relevant medication at index (UMD: antidepressants; BMD: mood stabilisers or antipsychotics; PSY: antipsychotics).

Complementary analyses and additional visual illustrations of findings were appended supplementary. All analyses were conducted in R version 4.2.3 employing the lme4 (version 1.1_18_1), emmeans (version 1.8.1_1), complexHeatmap [43] (version 3.18) and effectsize (version 0.8.1) packages. The level of significance was set as *p* < 0.05 when frequentist statistics were conducted.

## Results

### Sample characteristics

A total of 76,534 individuals received an SMD index diagnosis at SLaM in the study period; 21,156 were excluded due to no data before the index date, and 28,403 were excluded due to no data before the antecedent period (Supplementary Fig. 2). The final sample consisted of 26,975 individuals (UMD = 49.8%; BMD = 9.3%; PSY = 41.0%; mean follow-up 2.3 years; 68,359 person-years) with a mean age of 41.8 (SD = 17.4, median[IQR] = 39.8[23.7]) years at index, 55% of which were females and 52% of white self-reported ethnicity (Table 1, Supplementary Tables 7, 8).

**Table 1:** Baseline sociodemographic variables stratified by SMD group.

	Whole sample *N* = 26,975	UMD *N* = 13,422	BMD *N* = 2506	PSY *N* = 11,047
**Age** (mean, SD)	41.8 (17.4)	41.1 (18.7)	42.5 (15.7)	42.4 (16.1)
**Gender** (*n*, %)				
Female	14,939 (55)	8643 (64)	1546 (62)	4750 (43)
Male	12,019 (45)	4767 (36)	959 (38)	6293 (57)
Other	12 (< 0.1)	10 (< 0.1)	<10 (< 0.1)	<10 (< 0.1)
Missing	<10 (< 0.1)	<10 (< 0.1)	<10 (< 0.1)	<10 (< 0.1)
**Self-reported ethnicity** (*n*, %)				
Asian	1688 (6.3)	740 (5.5)	131 (5.2)	817 (7.4)
Black	6842 (25)	2195 (16)	340 (14)	4307 (39)
Mixed	874 (3.2)	493 (3.7)	73 (2.9)	308 (2.8)
White	14,158 (52)	7838 (58)	1643 (66)	4677 (42)
Other	1496 (5.5)	846 (6.3)	141 (5.6)	509 (4.6)
Missing	1917 (7.1)	1310 (9.8)	178 (7.1)	429 (3.9)
**Prescribed antidepressants** (*n*, %)	9431 (35)	5301 (39)	853 (34)	3277 (30)
**Prescribed antipsychotics** (*n*, %)	9426 (35)	1163 (8.7)	1104 (44)	7159 (65)
**Prescribed anxiolytics** (*n*, %)	5101 (19)	1698 (13)	627 (25)	2776 (25)
**Prescribed mood stabilisers** (*n*, %)	2704 (10)	486 (3.6)	1033 (41)	1185 (11)

All variables refer to information at index date. *UMD* Unipolar Mood Disorders, *BMD* Bipolar Mood Disorders, *PSY* Psychotic Disorders.

### Comparing the duration and first presentation of the prodrome between SMD groups

Among the study sample, 3660 individuals had no detectable NLP-derived prodromal features, leaving 23,315 individuals available for this analysis (Supplementary Table 9).

#### Duration of prodromal period

**Fig. 1:**
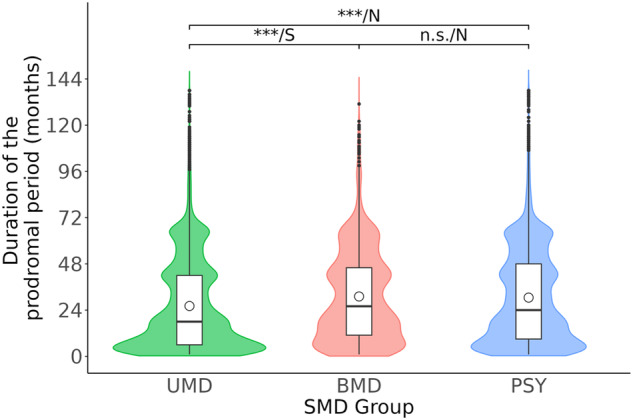
Duration of the prodrome. Violin plots representing the distribution of the duration of the prodromal period (months). The circle shape in the box plots represents mean values, the lower end of the box represents the lower quartile, the upper end of the box represents the upper quartile and the horizontal bar median values; vertical lines on each side of the box indicate the maximum and minimum values with the black points representing outliers. *P*-values/Effect sizes are represented for each comparison. *P*-values are represented with asterisks (***p < 0.0001, **p < 0.001, *p < 0.05 or n.s: non-significant [*p* > 0.05]). Effect sizes are shown as *N* (negligible d < 0.2), S (small, 0.2 ≤ d < 0.5), M (medium, 0.5 ≤ d < 0.8) or L (large, d ≥ 0.8). UMD Unipolar Mood Disorders, BMD Bipolar Mood Disorders, PSY Psychotic Disorders, *N* = 23,315.

The prodromal period was shorter in UMD (mean [SD], median [IQR] = 26.1[23.9], 18[36] months) than for both BMD (31.1[23.8], 26[35] months, d = 0.21 (95%CI = 0.16–0.25), *p* < 0.0001) and PSY (30.5[24.6], 24[39] months, d = 0.18 (95%CI = 0.15–0.21), *p* < 0.0001). There were no significant differences between PSY and BMD (d = −0.02, 95%CI = −0.07 to 0.02, *p* = 0.072) (Fig. 1, Supplementary Table 10).

When individuals over 35 years old and individuals with relevant medication at index were removed, the prodromal periods were slightly shorter but the pattern and comparisons remained unchanged (Supplementary Table 11).

#### First presentation of prodromal clusters

**Fig. 2:**
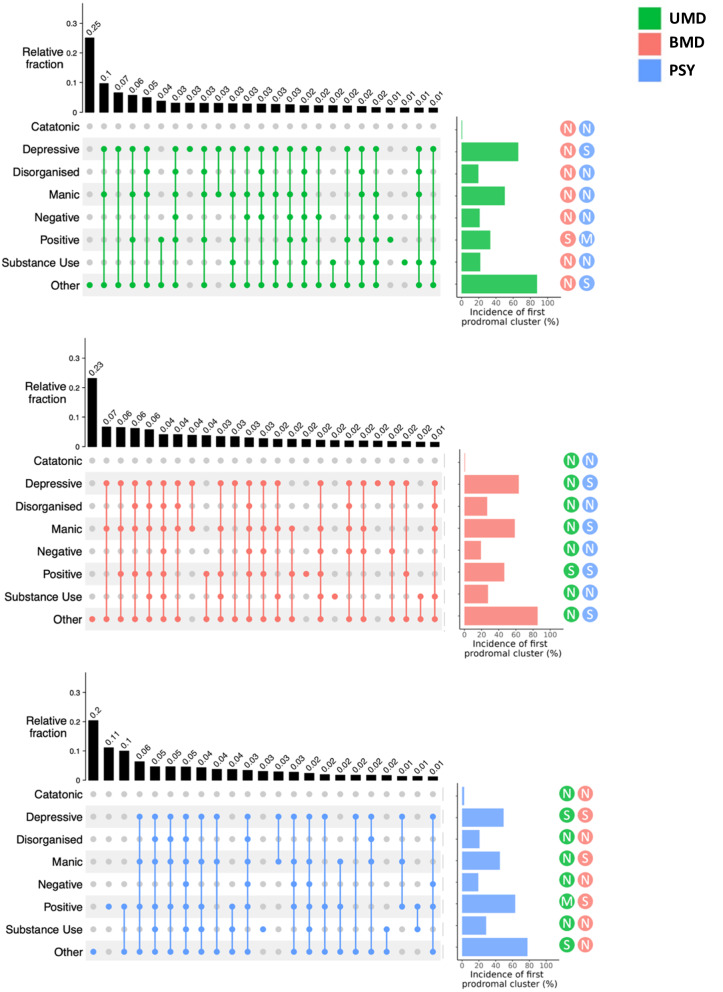
First presentation of the prodrome. UpSet plot representing the 20 most common combinations of first prodromal clusters in each SMD group. Vertical bars show the relative fraction (0-1) of each combination; horizontal bars show the total incidence (%) of each first prodromal cluster. Effect sizes (d) for the pair-wise difference between two SMD groups are shown in the colour of the comparison group as *N* (negligible d < 0.2), S (small, 0.2≤d < 0.5), M (medium, 0.5≤ d < 0.8) or L (large, d ≥ 0.8). UMD Unipolar Mood Disorders, BMD Bipolar Mood Disorders, PSY Psychotic Disorders, *N* = 23,315.

The majority of individuals (72.4% UMD; 74.4% BMD; 69.8% PSY) experienced a combination of two or more first prodromal clusters (Fig. 2, Supplementary Tables 12A and 13).

Across all SMD groups, the most common first presenting prodromal clusters consisted of the other (88% UMD; 85% BMD; 78% PSY) and depressive clusters (66% UMD; 63% BMD; 49% PSY). The catatonic (0.8% UMD; 0.8% BMD; 2.5% PSY) and negative clusters (21% UMD; 19% BMD; 19% PSY) were the least common (Fig. 2).

The most common first presentations of prodromal clusters for UMD were: other only (25%), depressive-manic-other (10%) and depressive-other (7%); for BMD: other only (23%), depressive-manic-other (7%) and depressive-manic-positive-other (6%); for PSY: other only (20%), positive only (11%) and positive-other (10%) (Fig. 2). First presentations of prodromal clusters were similar when individuals over 35 years old and individuals with relevant medication were excluded (Supplementary Results 1).

When comparing the incidence of the first presentation of all eight prodromal clusters (Fig. 2, Supplementary Table 12B) across all three SMD groups, a medium-sized effect was seen for the positive cluster (f = 0.30, 95%CI = 0.28–0.31) with only small/negligible effect sizes observed for other clusters. In pair-wise comparisons between two SMD groups, medium effect sizes were observed only for a higher incidence of the positive cluster in PSY compared to UMD (d = 0.62, 95%CI = 0.60-0.65). All other pair-wise comparisons were associated with small/negligible effect sizes (Supplementary Table 12B).

When individuals over 35 years old and individuals with relevant medication were excluded, there were only minor changes in the overall pattern of results. (Supplementary Table 12C).

### Comparing the time course of prodromal clusters between SMD groups

**Fig. 3:**
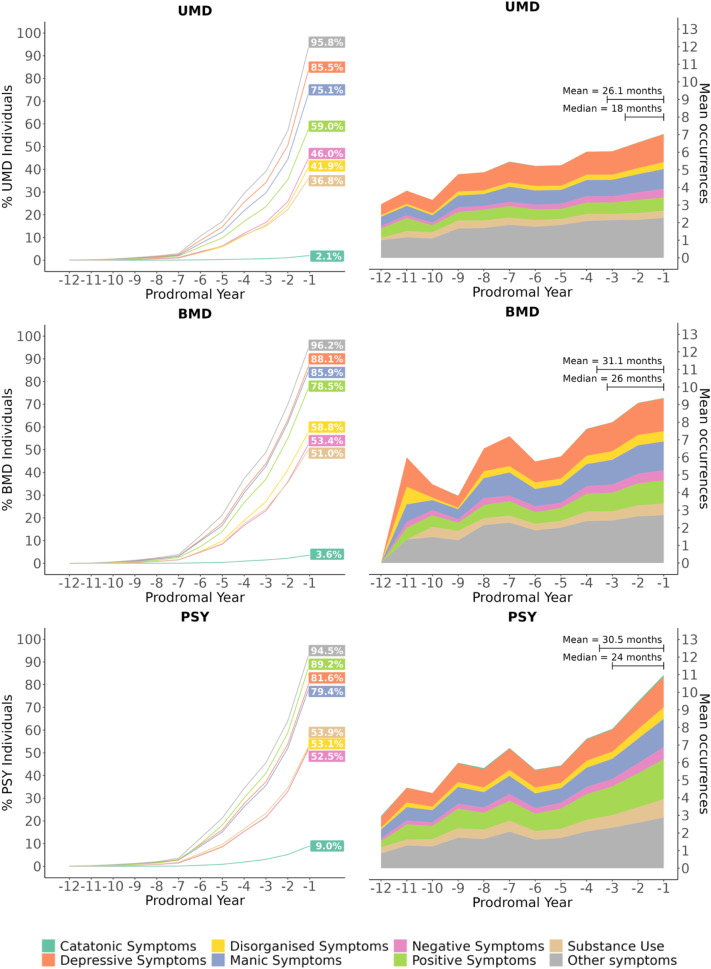
Time course of the prodrome. (Left) Line graph showing the cumulative proportion of individuals in each SMD group who experience each prodromal cluster by prodromal year. (Right) Stacked line graph showing the annualised mean occurrences of each prodromal cluster by each prodromal year. Mean and median prodromal periods (in months) are depicted in the right-hand corner for each SMD group. UMD Unipolar Mood Disorders, BMD Bipolar Mood Disorders, PSY Psychotic Disorders, *N* = 26,975.

Annualised mean occurrences (see Supplementary Table 14 for raw data) increased over time across all clusters (Fig. 3, Supplementary Table 15A). Adding an interaction term for SMD group improved model fit across all prodromal clusters (ΔAICc>65), with occurrences tending to diverge on approach to SMD onset (Supplementary Fig. 3, Supplementary Table 15A). The Q + I model produced the best fit across all clusters, except for catatonic, where L + I had a marginally better fit (Supplementary Table 15B).

There was a significant interaction between PSY and linear time for the catatonic, disorganised, manic, negative, positive, substance use and other clusters (*p* < 0.048), between BMD and linear time for the manic cluster (*p* = 0.021), and between PSY and quadratic time for the manic, positive, substance use and other clusters (*p* < 0.024) compared to UMD (Supplementary Table 15C).

When individuals over 35 years old were excluded there was no change in the pattern of results. Model fit was additionally greater in the linear interaction models across the depressive, disorganised, manic, positive and substance use clusters, and in the linear model (without interaction) for the negative symptom cluster, when individuals with relevant medication were excluded (Supplementary Table 15D, E).

### Comparing the transdiagnosticity of prodromal features between SMD groups

To meet the TRANSD criteria, we defined the gold standard by including specific primary ICD-10 diagnoses and by providing their codes (Supplementary Tables 6, 7), acknowledged the primary outcome of this study, defined the transdiagnostic construct in the methods, appraised it across 90 diagnoses (18 UMD; 13 BMD; 59 for PSY), and across three diagnostic spectra (UMD, BMD and PSY), performed two types of comparative analyses, but could not externally validate our findings.

A total of 28 prodromal features with near-zero variance were identified (Supplementary Table 16).

**Fig. 4:**
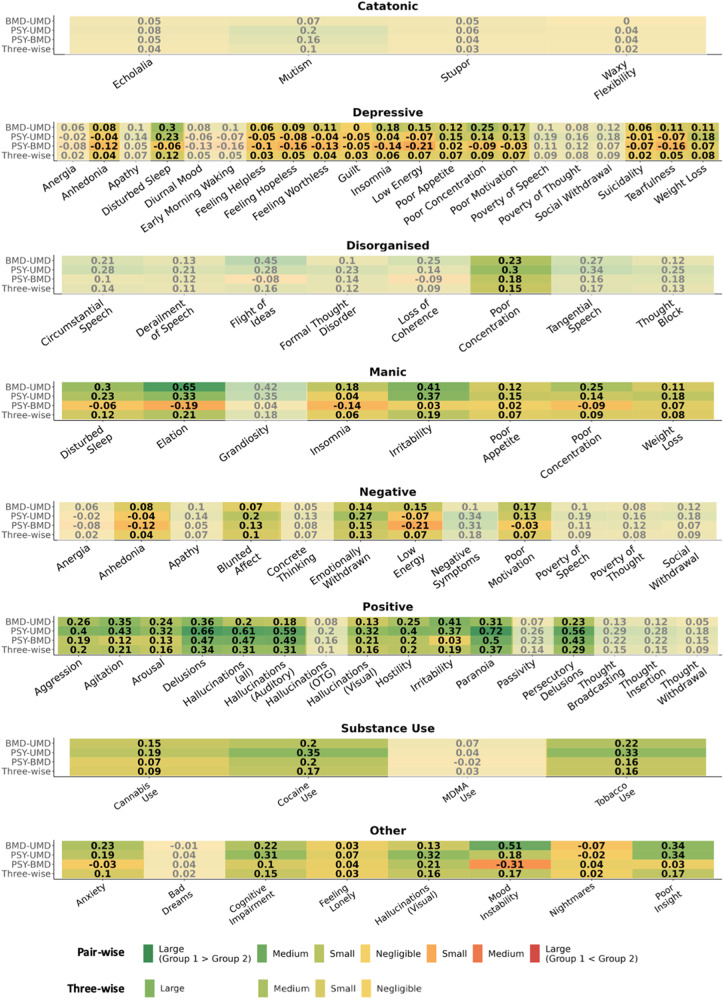
Transdiagnosticity of the prodrome. Heat maps representing the pair-wise and three-wise discriminability scores for each prodromal feature. Positive d values indicate greater mean occurrence in the first group listed compared to the second one, and viceversa. Features with transparency have near-zero variance, i.e. were rarely endorsed. UMD: Unipolar Mood Disorders, BMD: Bipolar Mood Disorders, PSY: Psychotic Disorders, *N* = 26,975.

The three-wise discriminability analysis (Fig. 4) showed medium discriminability scores for paranoia (f = 0.37, 95%CIs=0.35, 0.38), delusions (f = 0.34, 95%CIs = 0.33, 0.36), hallucinations (all) (f = 0.31, 95%CIs = 0.30, 0.33), auditory hallucinations (f = 0.31, 95%CIs = 0.29, 0.32), and persecutory delusions (f = 0.29, 95%CIs = 0.28, 0.30) across UMD, BMD and PSY.

The pair-wise discriminability analysis (Fig. 4) showed medium discriminability scores for paranoia (d = 0.72, 95%CIs = 0.69, 0.74), delusions (d = 0.66, 95%CIs = 0.64, 0.69), hallucinations (all) (d = 0.61, 95%CIs=0.58, 0.64), auditory hallucinations (d = 0.59, 95%CIs = 0.56, 0.62), and persecutory delusions (d = 0.56, 95%CIs = 0.53, 0.59) between PSY-UMD; for elation (d = 0.65, 95%CIs = 0.59, 0.71) and mood instability (d = 0.51, 95%CIs = 0.46, 0.56) between BMD-UMD; for paranoia (d = 0.50, 95%CIs = 0.46, 0.54) between PSY-BMD.

All the remaining three-wise and pair-wise discriminability scores were small/negligible (see also Supplementary Tables 17, 18 and Supplementary Results 2). When individuals over 35 years old were removed, results were largely unchanged but agitation and cannabis use also showed medium effect sizes, driven by higher occurrences in PSY compared to UMD (Supplementary Table 19). There were no differences in results when individuals with relevant medication were removed (Supplementary Table 19).

## Discussion

This study identified only negligible/small differences in the duration and first presentation of the prodrome across three SMD groups. In terms of time course, we found that prodromal features intensified when approaching the onset of SMD. Furthermore, with the exception of positive symptoms, most prodromal features appear to be transdiagnostic.

This study analysed 26,975 individuals monthly for up to 12 years preceding the onset of their disorder. To our best knowledge, this is the largest study characterising the prodrome for SMD (approximately 63 times larger than the largest previously published look-back study in BMD) [6]. It is also the first combining NLP and EHR, thus delivering a rich source of information close to clinical practice and sampled with high granularity over a very long look-back period. Furthermore, it endorsed a comprehensive and unprecedentesd transdiagnostic approach across 90 ICD diagnoses (18 UMD, 13 BMD and 59 PSY) and across three diagnostic spectra (UMD, BMD and PSY), with adherence to transdiagnostic reporting guidelines [6, 17, 20, 21, 44].

First, this study corroborates a detectable prodrome not only in PSY but also in BMD and UMD, with 72% of SMD individuals having EHR data before their diagnosis and 49% of these individuals with NLP-derived prodromal features at least six months before their diagnosis. However, given the risk enrichment in secondary mental health care, it is reasonable to assume that the duration of the prodrome is longer and the prevalence of prodromal features is lower in the general population than what we have observed in this setting. For example, outside secondary care, individuals with BMD may experience prodromal symptoms up to 11 years before an initial diagnosable mood episode [6]. At the same time, the presence of a prodrome in most EHR cases highlights a pragmatic window for preventing SMD onset for those already being treated in secondary mental health care. These early detection and preventive opportunities are further informed by our finding that the SMD prodromes mapped in secondary healthcare are relatively long, with a median of 18 months for UMD, 26 months for BMD and 24 months for PSY and are highly variable, with a range of 1–138 months for UMD, 1–131 for BMD and 1-138 for PSY, aligning with previous literature [2, 6, 19]. However, our results record only the peak of prodrome symptom intensity captured in secondary healthcare and cannot be considered to cover the full naturalistic course of the prodrome. Despite this, these durations are long enough for the detection of SMD before their onset to be clinically feasible. They also indicate that the short duration of clinical care (less than 24 months) [45] offered by most (72.4%) early detection services does not successfully capture the full range of the SMD prodrome durations and need to be extended [46–49].

Second, we found that the highest incidence of first presenting prodromal clusters included either non-specific (other) or depressive symptoms, with most people presenting with multiple prodromal clusters at the same time. In particular, when we analysed the specific prodromal features composing the other cluster, anxiety emerged as the most common across SMD (occurring in 66%, 63% and 53% of UMD, BMD and PSY, respectively). This finding aligns with prospective evidence indicating that comorbid anxiety disorders are common in individuals at clinical high risk for psychosis [50] and bipolar at risk [51–53]. Interestingly, cognitive impairment, poor insight, and poor concentration were also prevalent first prodromal features across SMD, albeit less frequent than anxiety. This suggests that subtle cognitive deficits are already abundant in the early phases before the onset of SMD. This observation aligns with our recent meta-analysis in prospective clinical high risk for psychosis showing relevant neurocognitive deficits compared to controls [54]. Within the depressive cluster, disturbed sleep was the most common first prodromal feature across SMD (occurring in 33%, 36% and 28% of UMD, BMD and PSY, respectively), supporting previous findings that show high prevalence of sleep disturbances in the earlier [55–57] and later [58] stages of SMD. Moreover, most individuals presented with multiple prodromal clusters at the same time. This suggests an initial transdiagnostic risk state, aligning with previous evidence indicating that non-specific and depressive symptoms tend to manifest earlier during the emergence of SMD [17, 59]. In fact, we confirmed negligible to small differences in the incidence of all first prodromal clusters, except for positive symptoms. These findings confirm the transdiagnostic first presentation of the SMD prodrome (at least in secondary mental health care) that has previously been theorised [60–64]. Transdiagnostic youth mental health services have started becoming implemented in clinical practice, such as the clinical high at-risk mental state (CHARMS), which includes prodromal bipolar disorder, depression and personality disorders in addition to psychosis [65].

Third, this study characterised the dynamic time course of the prodrome for SMD over 12 years. Its dynamic evolution differed across prodromal clusters, with most consistently increasing over time, while (non-specific) other symptoms appeared to exponentially increase approaching SMD onset. The rate of increase was especially high for manic, positive, substance use and other clusters in PSY. Disturbed sleep (manic), paranoia (positive), cannabis use (substance use) and anxiety (other) were the most commonly experienced features within these clusters across the prodromal period in PSY individuals. These symptom occurrences appear to signpost BMD and PSY onset and distinctly intensify over the prodromal period; their systematic screening and monitoring with automated NLP-based algorithms could represent an efficient strategy to boost early detection and preventive capacity. Previous research has already shown that using EHR-based approaches may inform early intervention strategies [66, 67], through prediction of clinical outcomes [68] including disorder onset [67, 69–73], cardiometabolic risk [74, 75] and treatment response [76]. Overall, these findings (Fig. 3) update and extend the seminal ABC study (conducted in a sample of 232 patients) [11], which was key to introducing the concept of a prodrome for psychosis.

Fourth, this study provides the first comparative atlas of diagnostic spectrum-specific and transdiagnostic prodromal features across SMD, with only positive symptoms (paranoia, delusions, hallucinations [all], auditory hallucinations and persecutory delusions) able to discriminate between all three prodromes (UMD, BMD and PSY) with a medium strength. This finding reflected our operationalisation of non-psychotic BMD and UMD and supports the role of psychometric instruments (CAARMS [77]/SIPS [78]) to detect psychosis risk, which largely focus on positive symptoms. Interestingly, occurrences of cognitive impairment features were higher in the PSY prodrome compared to UMD, confirming that subtle cognitive deficits are already present in the early phases preceding the onset of psychosis [54, 79]. On the other hand, the five most transdiagnostic and shared phenomena across SMD included feeling helpless, feeling lonely, guilt, nightmares and suicidality.

The present study has certain limitations that must be taken into account. First, the study reflects secondary healthcare clinical pathways and as such it is not naturalistically capturing the time course of the whole prodrome in the general population. Future studies will require data linkage with primary care and perinatal databases to achieve this. Second, while the use of EHRs in this study has high ecological validity, the symptoms recorded in clinical notes are not psychometrically validated. However, the use of structured diagnostic interviews can itself lead to selection biases [80], and there is meta-analytical evidence indicating administrative data recorded in EHR are generally predictive of true validated diagnoses [81]. Third, the mean age of this sample is relatively high compared to the expected peak of risk for mental disorders [82, 83] and proportion of individuals already receiving medication, meaning that we may not be capturing the full prodrome. However, our sensitivity analyses suggest that our main results are consistent when restricting to young individuals and those not having received medication prior to their diagnosis. Fourth, NLP tools generate some degree of noise as it is impossible to extract data from free text with 100% precision; clinician subjectivity, including structural or unconscious bias, can impact how symptoms are recorded for given individuals, thereby reducing standardisation of output [84]. We mitigated against this issue by pre-selecting NLP algorithms for an adequate level of precision (≥80%). Fifth, we could not externally validate these findings, and therefore their generalisability to other healthcare settings should be confirmed. Efforts are underway to conduct future validations within other NHS Trusts differing from SLaM in population sociodemographics, service configuration, and risk of psychosis onset [73].

## Conclusions

This large NLP-based analysis identified largely negligible/small differences in the duration and first presentation of the prodromes for UMD, BMD and PSY, as recorded in secondary mental healthcare. All  the prodromal clusters intensified when approaching the onset of SMD and all the prodromal features other than positive symptoms were transdiagnostic. These findings support proposals to develop transdiagnostic preventive services for SMD in secondary mental healthcare.

## Supplementary information

Supplementary Material

## Data Availability

The data accessed by CRIS remain within an NHS firewall and governance is provided by a patient-led oversight committee. Subject to these conditions, data access is encouraged and those interested should contact Robert Stewart (robert.stewart@kcl.ac.uk), CRIS academic lead. There is no permission for data sharing.
