# Supplementary material for: A transdiagnostic prodrome for severe mental disorders: an electronic health record study
Source: Mol Psychiatry. 2024 May 6;29(11):3305–15. doi: 10.1038/s41380-024-02533-5 (PMC11540905; doi:10.1038/s41380-024-02533-5)
Supplement: Supplementary file 1 — Supplementary Material [file 41380_2024_2533_MOESM1_ESM.docx]

**SUPPLEMENTARY MATERIAL**

Arribas M, Oliver D, Patel R et al. A transdiagnostic prodrome for severe mental disorders: an electronic health record study

*Table of Contents*

eIntroduction1 2

[eTable 1 The RECORD statement 3](#_Toc146189870)

[eMethods 1 SLaM service characteristics and use of CRIS data 7](#_Toc146189871)

[eTable 2 Definition of self-reported ethnicity 8](#_Toc146189872)

[*eTable 3 Medication Classification .........................................................................................................................9*](#_Toc146189872)

[eMethods 2 NLP algorithm development and validation 11](#_Toc146189873)

[eTable 4 Type and precision for 65 NLP algorithms 12](#_Toc146189874)

[eTable 5 Prodromal clusters 14](#_Toc146189875)

[eFigure 1 Study design. 15](#_Toc146189876)

[eTable 6 Operationalisation of ICD-10 diagnoses 16](#_Toc146189877)

[eMethods 3 TRANSD criteria for assessing transdiagnosticity of the SMD prodrome 17](#_Toc146189878)

[eFigure 2 Flow-chart of the study population. 18](#_Toc146189879)

[eTable 7 ICD-10 codes for index diagnoses stratified by SMD group 19](#_Toc146189882)

[eTable 8 Baseline sociodemographic variables for sensitivity analysis populations. 20](#_Toc146189883)

[eTable 9 Baseline sociodemographic variables stratified by inclusion in duration/first presentation analyses 21](#_Toc146189884)

[eTable 10 Duration of prodromal period stratified by SMD group 22](#_Toc146189885)

[eTable 11 Duration of prodromal period for sensitivity analysis populations stratified by SMD group 23](#_Toc146189886)

[eTable 12 First prodromal clusters 24](#_Toc146189887)

[eTable 13 First prodromal features 27](#_Toc146189889)

[eResults 1 First presentation of prodromal clusters in sensitivity analysis populations 30](#_Toc146189892)

[eTable 14 Number of feature occurrences and normalised frequency. 31](#_Toc146189893)

[eFigure 3 Plotted model predictions for prodromal clusters 33](#_Toc146189894)

[eTable 15 Mixed Linear Effects Model Results. 34](#_Toc146189895)

[eTable 16 Near-zero and non-near-zero variance features 40](#_Toc146189896)

[eTable 17 Discriminability scores for feature occurrences 42](#_Toc146189897)

[eTable 18 Discriminability scores for feature normalised frequency 44](#_Toc146189898)

[eResults 2 Discriminability analysis for feature normalised frequency 46](#_Toc146189899)

[eTable 19 Discriminability scores for feature occurrences in sensitivity analyses. 47](#_Toc146189900)

[eReferences 51](#_Toc146189901)

**eIntroduction 1**

The prodrome of psychotic disorders is extensively studied. Early detection services are recommended by clinical guidelines worldwide and aim to recognise prodromal symptoms and intervene to reduce the risk of transition to psychosis (1,2). Psychometric interviews (CAARMS (3), SIPS (4)) are used to identify individuals at risk for psychosis and focus on unusual thought content, non-bizarre ideas (e.g. suspiciousness/persecution), perceptual abnormalities and disorganised speech. These instruments perform well at predicting the onset of psychosis (5), however their prioritisation of attenuated positive psychotic symptoms puts less emphasis on other prodromal characteristics. Other studies investigating the prodromal symptoms of psychotic disorders (6,7) have identified prodromal symptoms outside of these that are commonly experienced, such as relationship disturbances, social withdrawal, irritability, sleeping issues, depression, restlessness, anxiety, thinking/concentration difficulties, worrying, lack of self-confidence, lack of energy, poor work and problems communicating.

For MDD, the most recent review (8) (k=25, n=1324) identifies sleep disturbances, fatigue, reduced energy, generalised anxiety and tension, irritability, and somatic complaints as the most commonly reported prodromal symptoms.

Similarly for BMD, the most common prodromal symptoms identified in a recent meta-analysis (9) (k=11 studies, n=1078 subjects) are: excessive energy (87%), excessive talkativeness (60%), racing thoughts (59%), elated mood (59%), decreased need for sleep (57%), irritable mood (54%), hyperactive behaviour (50%), and over-productive goal-directed (50%) behaviour.

# **eTable 1 The RECORD statement – checklist of items, extended from the STROBE statement, that should be reported in observational studies using routinely collected health data.**

|  | **Item No.** | **STROBE items** | **Location in manuscript where items are reported** | **RECORD items** | **Location in manuscript where items are reported** |
| --- | --- | --- | --- | --- | --- |
| **Title and abstract** | | | | | |
|  | 1 | (a) Indicate the study’s design with a commonly used term in the title or the abstract (b) Provide in the abstract an informative and balanced summary of what was done and what was found | Abstract | RECORD 1.1: The type of data used should be specified in the title or abstract. When possible, the name of the databases used should be included.  RECORD 1.2: If applicable, the geographic region and timeframe within which the study took place should be reported in the title or abstract.  RECORD 1.3: If linkage between databases was conducted for the study, this should be clearly stated in the title or abstract. | Abstract  Abstract  NA |
| **Introduction** | | | | | |
| Background rationale | 2 | Explain the scientific background and rationale for the investigation being reported | Introduction |  |  |
| Objectives | 3 | State specific objectives, including any prespecified hypotheses | Abstract, Introduction, Materials and methods |  |  |
| **Methods** | | | | | |
| Study Design | 4 | Present key elements of study design early in the paper | Abstract |  |  |
| Setting | 5 | Describe the setting, locations, and relevant dates, including periods of recruitment, exposure, follow-up, and data collection | Abstract, Materials and methods |  |  |
| Participants | 6 | *(a) Cohort study* - Give the eligibility criteria, and the sources and methods of selection of participants. Describe methods of follow-up  *Case-control study* - Give the eligibility criteria, and the sources and methods of case ascertainment and control selection. Give the rationale for the choice of cases and controls  *Cross-sectional study* - Give the eligibility criteria, and the sources and methods of selection of participants  *(b) Cohort study* - For matched studies, give matching criteria and number of exposed and unexposed  *Case-control study* - For matched studies, give matching criteria and the number of controls per case | Abstract  Materials and methods  NA  NA  NA  NA | RECORD 6.1: The methods of study population selection (such as codes or algorithms used to identify subjects) should be listed in detail. If this is not possible, an explanation should be provided.  RECORD 6.2: Any validation studies of the codes or algorithms used to select the population should be referenced. If validation was conducted for this study and not published elsewhere, detailed methods and results should be provided.  RECORD 6.3: If the study involved linkage of databases, consider use of a flow diagram or other graphical display to demonstrate the data linkage process, including the number of individuals with linked data at each stage. | Materials and methods  Materials and methods, referenced previous publications addressing the codes or algorithms used  NA |
| Variables | 7 | Clearly define all outcomes, exposures, predictors, potential confounders, and effect modifiers. Give diagnostic criteria, if applicable. | Materials and methods, Table 1 | RECORD 7.1: A complete list of codes and algorithms used to classify exposures, outcomes, confounders, and effect modifiers should be provided. If these cannot be reported, an explanation should be provided. | Materials and methods, supplementary material |
| Data sources/ measurement | 8 | For each variable of interest, give sources of data and details of methods of assessment (measurement).  Describe comparability of assessment methods if there is more than one group | Materials and methods |  |  |
| Bias | 9 | Describe any efforts to address potential sources of bias | Materials and methods |  |  |
| Study size | 10 | Explain how the study size was arrived at | Materials and methods  eFigure 2 |  |  |
| Quantitative variables | 11 | Explain how quantitative variables were handled in the analyses. If applicable, describe which groupings were chosen, and why | Statistical analysis |  |  |
| Statistical methods | 12 | (a) Describe all statistical methods, including those used to control for confounding  (b) Describe any methods used to examine subgroups and interactions  (c) Explain how missing data were addressed  (d) *Cohort study* - If applicable, explain how loss to follow-up was addressed  *Case-control study* - If applicable, explain how matching of cases and controls was addressed  *Cross-sectional study* - If applicable, describe analytical methods taking account of sampling strategy  (e) Describe any sensitivity analyses | Statistical analysis  Statistical analysis  Statistical analysis  Statistical analysis  NA  NA  Statistical analysis |  |  |
| Data access and cleaning methods |  | .. |  | RECORD 12.1: Authors should describe the extent to which the investigators had access to the database population used to create the study population.  RECORD 12.2: Authors should provide information on the data cleaning methods used in the study. | Discussion  NA |
| Linkage |  | .. |  | RECORD 12.3: State whether the study included person-level, institutional-level, or other data linkage across two or more databases. The methods of linkage and methods of linkage quality evaluation should be provided. | NA |
| **Results** | | | | | |
| Participants | 13 | (a) Report the numbers of individuals at each stage of the study (*e.g.*, numbers potentially eligible, examined for eligibility, confirmed eligible, included in the study, completing follow-up, and analysed)  (b) Give reasons for non-participation at each stage.  (c) Consider use of a flow diagram | Results  NA  eFigure 1 | RECORD 13.1: Describe in detail the selection of the persons included in the study (*i.e.,* study population selection) including filtering based on data quality, data availability and linkage. The selection of included persons can be described in the text and/or by means of the study flow diagram. | Results |
| Descriptive data | 14 | (a) Give characteristics of study participants (*e.g.*, demographic, clinical, social) and information on exposures and potential confounders  (b) Indicate the number of participants with missing data for each variable of interest  (c) *Cohort study* - summarise follow-up time (*e.g.*, average and total amount) | Table 1  eFigure 1  Results |  |  |
| Outcome data | 15 | *Cohort study* - Report numbers of outcome events or summary measures over time  *Case-control study* - Report numbers in each exposure category, or summary measures of exposure  *Cross-sectional study* - Report numbers of outcome events or summary measures | Results and Figure 2  NA  NA |  |  |
| Main results | 16 | (a) Give unadjusted estimates and, if applicable, confounder-adjusted estimates and their precision (e.g., 95% confidence interval). Make clear which confounders were adjusted for and why they were included  (b) Report category boundaries when continuous variables were categorized  (c) If relevant, consider translating estimates of relative risk into absolute risk for a meaningful time period | Results  NA  NA |  |  |
| Other analyses | 17 | Report other analyses done—e.g., analyses of subgroups and interactions, and sensitivity analyses | Results |  |  |
| **Discussion** | | | | | |
| Key results | 18 | Summarise key results with reference to study objectives | First paragraph of discussion |  |  |
| Limitations | 19 | Discuss limitations of the study, taking into account sources of potential bias or imprecision. Discuss both direction and magnitude of any potential bias | Discussion (strengths and weaknesses of the study section) | RECORD 19.1: Discuss the implications of using data that were not created or collected to answer the specific research question(s). Include discussion of misclassification bias, unmeasured confounding, missing data, and changing eligibility over time, as they pertain to the study being reported. | Discussion (implications for clinical practice and strengths and weaknesses of the study sections) |
| Interpretation | 20 | Give a cautious overall interpretation of results considering objectives, limitations, multiplicity of analyses, results from similar studies, and other relevant evidence | Discussion (interpretation of findings and strengths and weaknesses of the study sections) |  |  |
| Generalisability | 21 | Discuss the generalisability (external validity) of the study results | Discussion |  |  |
| **Other Information** | | | | | |
| Funding | 22 | Give the source of funding and the role of the funders for the present study and, if applicable, for the original study on which the present article is based | Financial support statement in the abstract |  |  |
| Accessibility of protocol, raw data, and programming code |  | .. |  | RECORD 22.1: Authors should provide information on how to access any supplemental information such as the study protocol, raw data, or programming code. | Statistical analysis, supplementary material |

**eMethods 1** **SLaM service characteristics and use of CRIS data**

With respect to service characteristics, SLaM early intervention teams serve an overall catchment area of 443 050 people aged 16–35 years (2017) (10), and are amongst the largest of their kinds in the UK and worldwide.

The trust is digitized and paper-free (11), with each patient having a personal EHR since 2007 (12,13). SLaM healthcare professionals are legally required to update these records (11). The SLaM register contains all these clinical records which are constantly updated throughout the patient’s care, irrespective of any discharges from and/or referrals to other services.

To limit potential identifiability of any individuals, no data representing fewer than ten individuals are presented.

# **eTable 2 Definition of self-reported ethnicity according to UK Office of National Statistics**

| **Ethnic group** | **Self-reported ethnicity as recorded in EHR** |
| --- | --- |
| Black | Black or Black British - African  Black or Black British - Caribbean  Black or Black British - Any other Black background |
| White | White - British  White - Irish  White - Any other White background |
| Asian | Asian or Asian British - Bangladeshi  Asian or Asian British - Indian  Asian or Asian British - Pakistani  Asian or Asian British - Any other Asian background  Other Ethnic Groups - Chinese |
| Mixed | Mixed - White and Asian  Mixed - White and Black African  Mixed - White and Black Caribbean  Mixed - Any other mixed background |
| Other | Other Ethnic Groups - Any other ethnic group |
| Missing | Not Known  Not Recorded |

**eTable 3 Medication Classification**

| **Antipsychotics** | **Antidepressants** | **Mood Stabilisers** | **Anxiolytics** |
| --- | --- | --- | --- |
| Amisulpride (Solian) | Agomelatine (Valdoxan) | Carbamazepine (Carbagen, Tegretol) | Alprazolam |
| Aripiprazole (Abilify) | Amitriptyline (Triptafen) | Desitrend | Bio-Melatonin |
| Asenapine (Sycrest) | Amitriptyline hydrochloride | Lamotrigine (Lamictal) | Buspirone |
| Benperidol | Buproprion (Wellbutrin, Zyban) | Levetiracetam (Keppra) | Buspirone hydrochloride |
| Cariprazine (Reagila) | Citalopram (Cipramil) | Lithium Carbonate (Camcolit, Liskonum, Priadel) | Chloral Hydrate |
| Chlorpromazine (Thorazine, Largactil) | Citalopram Hydrobromide | Lithium Citrate (Priadel) | Chlordiazepoxide |
| Clozapine (Clozaril) | Citalopram Hydrochloride | Pregabalin (Alzain, Lecaent, Lyrica, Rewisca) | Chlorhexidine |
| Denzapine | Clomipramine (Anafranil) | Sodium Valproate (Convulex, Epilim, Epilim Chronosphere, Episenta, Epivalk, Orlept) | Clomethiazole |
| Droperidol | Dosulepin (Dothiepin, Prothiaden) | Topiramate (Topamax) | Clonazepam |
| Flupentixol Decanoate (Psytixol) | Doxepin | Valproic Acid (Valproate) | Diazepam |
| Flupentixol Hydrochloride (Depixol) | Duloxetine (Cymbalta, Duciltia) |  | Diazepam Rectube |
| Fluphenazine | Escitalopram (Cipralex) |  | Flurazepam |
| Fluphenazine Decanoate (Modecate) | Fluoxetine (Olena, Oxactin, Prozac, Prozep) |  | Loprazolam |
| Fluphenazine Hydrochloride | Flupentixol (Fluanxol) |  | Lorazepam |
| Haloperidol (Haldol, Seranace) | Flupentixol Hydrochloride (Depixol) |  | Lormetazepam |
| Haloperidol Decanoate | Fluvoxamine (Faverin) |  | Melatonin |
| Levomepromazine (Nozinan) | Imipramine |  | Nitrazepam |
| Levomepromazine Hydrochloride | Isocarboxazid |  | Oxazepam |
| Levomepromazine Maleate (Levinan) | Lofepramine |  | Rivotril |
| Lurasidone (Latuda) | Maprotiline |  | Temazepam |
| Melperone | Mianserin |  |  |
| Olanzapine (Zalasta, Zypadhera, Zyprexa) | Mirtazapine (Zispin) |  |  |
| Olanzapine embonate | Moclobemide (Manerix) |  |  |
| Paliperidone (Invega, Trevicta, Xeplion) | Nefazodone |  |  |
| Penfluridol | Nortriptyline |  |  |
| Pericyazine | Opipromal (Insidon) |  |  |
| Perphenazine (Fentazin) | Paroxetine (Seroxat) |  |  |
| Pimozide (Orap) | Reboxetine (Edronax) |  |  |
| Pipotiazine (Piportil) | Sertraline (Lustral) |  |  |
| Pipotiazine palmitate | Tianeptine (Coaxil, Stablon) |  |  |
| Promazine | Tranylcypromine |  |  |
| Quetiapine (Atrolak, Biquelle, Brancico, Ebesque, Mintreleq, Neotiapim, Psyquet, Seroquel, Sondate, Zaluron) | Trazodone (Molipaxin) |  |  |
| Risperidone (Risperdal) | Trimipramine (Surmontil) |  |  |
| Sulpiride (Dolmatil) | Tryptophan (Optimax) |  |  |
| Thioridazine (Melleril) | Venlafaxine (Alventa, Amphero, Depefex, Efexor, Politid, Rodomel, Sunveniz, Tonpular, Venax, Vencarm, Venlablue, Venladex, Venlalic, Vensir, ViePax, Winfex) |  |  |
| Trifluoperazine (Stelazine) | Vortioxetine (Brintellix) |  |  |
| Zaponex |  |  |  |
| Ziprasidone (Geodon) |  |  |  |
| Zuclopenthixol (Clopixol, Clopixol Acuphase) |  |  |  |
| Zuclopenthixol Acetate |  |  |  |
| Zuclopenthixol Decanoate |  |  |  |
| Zuclopenthixol Hydrochloride |  |  |  |

# **eMethods 2 NLP algorithm development and validation**

The CRIS symptom algorithms (e.g. ‘guilt’) have been developed using machine learning approaches against gold standard training sets manually annotated for positive, negative and unknown (irrelevant) mentions. As such, they are able to exclude language features such as negation (e.g. ‘patient denies guilt’, ‘patient has no guilt’) and irrelevant mentions (e.g. ‘his mother felt guilty’). Patterns of failure driving false positives are identified through manual testing of algorithm output (e.g. ‘ZZZZZ was found guilty of stealing’); the machine learning classifier is then trained on these false positives to ignore these and similar statements in an iterative process of testing and redeveloping until acceptable precision is achieved. Patterns of failure identified through testing can be found in the CRIS service’s comprehensive online NLP algorithm library provided at <https://www.maudsleybrc.nihr.ac.uk/facilities/clinical-record-interactive-search-cris/cris-natural-language-processing/>.

The performance of each NLP algorithm was measured with precision (proportion of true positive instances of total NLP-labelled positive instances) and recall (proportion of true positive instances of all positive instances in the text). As EHRs provide multiple opportunities for term detection, we favour precision over recall, using only NLP algorithms with at least 80% precision (see eTable 4 for a final list of NLP algorithms employed).

These algorithms were manually validated by an independent researcher at the SLaM Biomedical Research Centre Nucleus prior to the current research project. The programme for algorithms validation was responsive to the specific needs of scheduled CRIS research activities and therefore the approach was not standardised. For example, depression symptom algorithms have been validated against records for SLaM individuals who had ever had a depression diagnosis; other algorithms have been validated against records for all individuals on the SLaM register.

# **eTable 4 Type and precision for 65 NLP algorithms selected in the current study**

Precision values are taken from the CRIS Natural Language Processing Library (2021) (14), and were obtained by randomly selecting *n* positive annotations from each algorithm for a specified cohort, limited to one annotation per patient ID. Precision was then calculated as the ratio of the number of relevant (true positive) instances retrieved out of the total NLP-labelled positive instances (including irrelevant [false positive] and relevant [true positive] instances) for each NLP algorithm.

| **NLP algorithms** | **Cohort** | **Annotations validated (n)** | **Precision (%)** |
| --- | --- | --- | --- |
| Aggression | Random sample | 100 | 90 |
| Agitation | Random sample | 100 | 85 |
| Anergia | Random sample | 100 | 84 |
| Anhedonia | Random sample | 100 | 94 |
| Anxiety | Random sample | 100 | 94 |
| Apathy | Random sample | 100 | 94 |
| Arousal | Random sample | 100 | 89 |
| Bad dreams | CAMHS events | 100 | 92 |
| Blunted affect | Random sample | 100 | 98 |
| Cannabis use | All patients | 100 | 88 |
| Circumstantiality | Random sample | 100 | 97 |
| Cocaine use | Random sample | 30 | 97 |
| Cognitive impairment | Patients with F20 | 100 | 84 |
| Concrete thinking | Random sample | 146 | 91 |
| Delusional thinking | Random sample | 100 | 90 |
| Derailment | Random sample | 100 | 87 |
| Disturbed sleep | Random sample | 100 | 89 |
| Diurnal mood | Random sample | 100 | 86 |
| Early morning wakening | Random sample | 100 | 96 |
| Echolalia | Random sample | 100 | 96 |
| Elation | Random sample | 100 | 95 |
| Emotional withdrawal | Random sample | 100 | 87 |
| Feeling helpless | Random sample | 100 | 92 |
| Feeling hopeless | Random sample | 100 | 88 |
| Feeling lonely | Random sample | 100 | 87 |
| Feeling worthless | Random sample | 100 | 91 |
| Flight of ideas | Random sample | 100 | 89 |
| Formal thought disorder | Random sample | 100 | 85 |
| Grandiosity | Random sample | 100 | 89 |
| Guilt | Random sample | 100 | 84 |
| Hallucinations (all) | Random sample | 100 | 90 |
| Hallucinations (auditory) | Random sample | 100 | 92 |
| Hallucinations (OTG: olfactory, tactile, gustatory) | Random sample | 100 | 86 |
| Hallucinations (visual) | Random sample | 100 | 83 |
| Hostility | Random sample | 100 | 86 |
| Insomnia | Random sample | 100 | 97 |
| Irritability | Random sample | 100 | 99 |
| Loss of coherence | Random sample | 158 | 85 |
| Low energy | CAMHS events | 100 | 89 |
| MDMA use | Random sample | 100 | 94 |
| Mood instability | Random sample | 100 | 91 |
| Mutism | Random sample | 100 | 95 |
| Negative symptoms | Random sample | 100 | 87 |
| Nightmares | Random sample | 100 | 89 |
| Paranoia | Random sample | 100 | 89 |
| Passivity | Random sample | 100 | 88 |
| Persecutory ideation | Random sample | 100 | 80 |
| Poor appetite | Random sample | 100 | 89 |
| Poor concentration | Random sample | 100 | 88 |
| Poor insight | Random sample | 100 | 85 |
| Poor motivation | Random sample | 100 | 95 |
| Poverty of speech | Random sample | 100 | 88 |
| Poverty of thought | Random sample | 100 | 98 |
| Social withdrawal | Random sample | 100 | 98 |
| Stupor | Random sample | 100 | 88 |
| Suicidality | CAMHS events | 100 | 87 |
| Tangential speech | Random sample | 100 | 90 |
| Tearfulness | Random sample | 100 | 94 |
| Thought block | Random sample | 100 | 92 |
| Thought broadcast | Random sample | 100 | 84 |
| Thought insertion | Random sample | 100 | 84 |
| Thought withdrawal | Random sample | 100 | 84 |
| Tobacco use | Random sample | 118 | 90 |
| Waxy flexibility | Random sample | 100 | 81 |
| Weight loss | Random sample | 100 | 80 |

**eTable** **5 Prodromal clusters employed to stratify the 65 prodromal features investigated in this study.**

| **Prodromal Cluster (number of prodromal features in the prodromal cluster)** | | | | | | | | |
| --- | --- | --- | --- | --- | --- | --- | --- | --- |
| **Catatonic**  **(0-4)** | **Depressive**  **(0-21)** | **Disorganised**  **(0-8)** | **Manic**  **(0-8)** | **Negative**  **(0-12)** | **Positive**  **(0-16)** | **Substance use**  **(0-4)** | **Other**  **(0-8)** |  |
| Echolalia | Anergia | Circumstantiality | Disturbed sleep | Anergia | Aggression | Cannabis use | Anxiety |  |
| Mutism | Anhedonia | Derailment of speech | Elation | Anhedonia | Agitation | Cocaine use | Bad dreams |  |
| Stupor | Apathy | Flight of ideas | Grandiosity | Apathy | Arousal | MDMA use | Cognitive impairment |  |
| Waxy flexibility | Disturbed sleep | Formal thought disorder | Insomnia | Blunted affect | Delusions | Tobacco use | Feeling lonely |  |
|  | Diurnal mood | Loss of coherence | Irritability | Concrete thinking | Hallucinations (all) |  | Hallucinations (visual) |  |
|  | Early morning wakening | Poor concentration | Poor appetite | Emotionally withdrawn | Hallucinations (auditory) |  | Mood instability |  |
|  | Guilt | Tangential speech | Poor concentration | Low energy | Hallucinations (OTG) |  | Nightmares |  |
|  | Feeling helpless | Thought block | Weight loss | Negative symptoms | Hallucinations (visual) |  | Poor insight |  |
|  | Feeling hopeless |  |  | Poor motivation | Hostility |  |  |  |
|  | Feeling worthless |  |  | Poverty of speech | Irritability |  |  |  |
|  | Insomnia |  |  | Poverty of thought | Paranoia |  |  |  |
|  | Low energy |  |  | Social withdrawal | Passivity |  |  |  |
|  | Poor appetite |  |  |  | Persecutory delusions |  |  |  |
|  | Poor concentration |  |  |  | Thought broadcast |  |  |  |
|  | Poor motivation |  |  |  | Thought insertion |  |  |  |
|  | Poverty of speech |  |  |  | Thought withdrawal |  |  |  |
|  | Poverty of thought |  |  |  |  |  |  |  |
|  | Social withdrawal |  |  |  |  |  |  |  |
|  | Suicidality |  |  |  |  |  |  |  |
|  | Tearfulness |  |  |  |  |  |  |  |
|  | Weight loss |  |  |  |  |  |  |  |

# **eFigure 1 Study design. The prodromal period varied between individuals with the maximum prodromal period running between T-144mo and T-6mo. Each individual may have their first occurrence of prodromal features at a different time-point, which leads to varying durations of the prodromal period.**


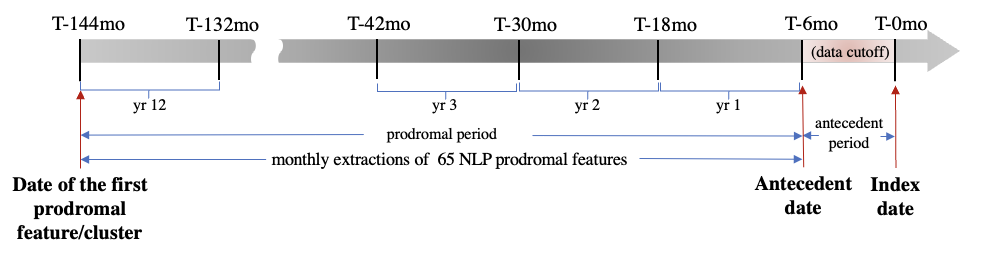


**eTable 6 Operationalisation of ICD-10 diagnoses employed in the current study (**[**https://icd.who.int/browse10/**](https://icd.who.int/browse10/)**), stratified by SMD group and excluded diagnoses (including organic disorders, non-SMD and unspecified mental disorders).**

| **Diagnostic group** | **Specific ICD-10 code** | **Specific ICD-10 diagnosis, version 2016** |
| --- | --- | --- |
| **Unipolar mood disorders (UMD)** | F32.x (excluding F32.3) | Non-psychotic depressive episode |
|  | F33.x (excluding F33.3) | Non-psychotic recurrent depressive disorder |
|  | F34.x (excluding F34.0) | Persistent unipolar mood disorders |
|  | F34.8, F34.9, F38, F39, F38.x, F39.x | Unspecified mood disorders |
| **Bipolar mood disorders (BMD)** | F30.x (excluding *.2) | Non-psychotic manic episode |
|  | F31.x (excluding F31.2 and F31.5) | Non-psychotic bipolar affective disorder |
|  | F34.0 | Cyclothymia |
| **Psychotic disorders (PSY)** | [F10-F19].4, [F10-F19].5, [F10-F19].7 | Mental and behavioural disorders due to psychoactive substance use with psychotic symptoms or delirium |
|  | F20-29 | Schizophrenia, schizotypal and delusional disorders |
|  | F30.2 | Mania with psychotic symptoms |
|  | F31.2 | Bipolar affective disorder, current episode manic with psychotic symptoms |
|  | F31.5 | Bipolar affective disorder, current episode severe depression with psychotic symptoms |
|  | F32.3 | Severe depressive episode with psychotic symptoms |
|  | F33.3 | Recurrent depressive disorder, current episode severe with psychotic symptoms |
|  | F53.1 | Severe mental and behavioural disorders associated with the puerperium, not elsewhere classified (post-partum psychosis) |
| **Excluded** | F00-F09 | Organic, including symptomatic, mental disorders |
|  | F10-19 (excluding *.4, *.5 and *.7) | Mental and behavioural disorders due to psychoactive substance use |
|  | F40-48 | Neurotic, stress-related and somatoform disorders |
|  | F50-59 (excluding F53.1) | Behavioural syndromes associated with physiological disturbances and physical factors |
|  | F60-69 | Disorders of adult personality and behaviour |
|  | F70-79 | Mental retardation |
|  | F80-89 | Disorders of psychological development |
|  | F90-98 | Behavioural and emotional disorders with onset usually occurring in childhood and adolescence |
|  | F99 | Unspecified mental disorder |

# **eMethods 3 TRANSD criteria for assessing transdiagnosticity of the SMD prodrome**

TRANSD criteria have been fully detailed elsewhere (15,16) and already employed in various publications (17,18). Briefly, TRANSD criteria requires appraising the transparent definition of the gold standard (criterion T), reporting the primary outcome (criterion R), appraising the transdiagnostic approach (criterion A), numerate the diagnostic categories and spectra (criterion N), show the degree of change of the transdiagnostic construct compared to diagnostic-specific constructs with comparative analyses (criterion S), and demonstrate the generalizability of the transdiagnostic construct (criterion D) (15,16).

# **eFigure 2 Flow-chart of the study population.**

Individuals receiving a primary ICD-10 diagnosis of SMD within SLaM between 01/01/2008 and 10/08/21

(N=76 534)

Individuals with data recorded exclusively after index date

(N=21 156) or with data recorded exclusively in the antecedent period

(N=28 403)

Final study population for time course and transdiagnosticity analyses

(N=26 975)

(UMD N=13 422

BMD N=2 506

PSY N=11 047)

Individuals with no detectable NLP-derived prodromal features

(N=3 660)

Final study population for duration and first presentation analyses (N=23 315)

(UMD N=11 445

BMD N=2 155

PSY N=9 715)

# **eTable** **7 ICD-10 codes for index diagnoses stratified by SMD group (N = 26 975).**

| **ICD-10 code** | **UMD**  **N = 13 422** | **BMD**  **N = 2 506** | **PSY**  **N = 11 047** |
| --- | --- | --- | --- |
|  | n (%) | n (%) | n (%) |
| F30.x (excluding F30.2) | 0.00 (0.00) | 109 (4.35) | 0.00 (0.00) |
| F31.x (excluding F31.2 and F31.5) | 0.00 (0.00) | 2 279 (90.94) | 0.00 (0.00) |
| F34.0 | 0.00 (0.00) | 118 (4.71) | 0.00 (0.00) |
| F32.x (excluding F32.3) | 8 829 (65.78) | 0.00 (0.00) | 0.00 (0.00) |
| F33.x (excluding F33.3) | 3 317 (24.71) | 0.00 (0.00) | 0.00 (0.00) |
| F34.1 | 188 (1.40) | 0.00 (0.00) | 0.00 (0.00) |
| F34.8, F34.9, F38, F39, F38.x, F39.x | 945 (7.04) | 0.00 (0.00) | 0.00 (0.00) |
| F34 | 143 (1.07) | 0.00 (0.00) | 0.00 (0.00) |
| [F10-F19].4, [F10-F19].5, [F10-F19].7 | 0.00 (0.00) | 0.00 (0.00) | 376 (3.40) |
| F20 | 0.00 (0.00) | 0.00 (0.00) | 5 407 (48.95) |
| F22, F24 | 0.00 (0.00) | 0.00 (0.00) | 634 (5.74) |
| F23 | 0.00 (0.00) | 0.00 (0.00) | 810 (7.33) |
| F25 | 0.00 (0.00) | 0.00 (0.00) | 1 029 (9.31) |
| F28, F29 | 0.00 (0.00) | 0.00 (0.00) | 1 349 (12.21) |
| F30.2 | 0.00 (0.00) | 0.00 (0.00) | 79 (0.72) |
| F31.2 | 0.00 (0.00) | 0.00 (0.00) | 311 (2.82) |
| F31.5 | 0.00 (0.00) | 0.00 (0.00) | 52 (0.47) |
| F32.3 | 0.00 (0.00) | 0.00 (0.00) | 748 (6.77) |
| F33.3 | 0.00 (0.00) | 0.00 (0.00) | 240 (2.17) |
| F53.1 | 0.00 (0.00) | 0.00 (0.00) | 12 (0.11) |

**eTable 8 Baseline sociodemographic variables for sensitivity analysis populations. All variables refer to information at index diagnosis date. UMD: Unipolar Mood Disorders, BMD: Bipolar Disorders, PSY: Psychotic Disorders.**

|  | **≤35 years of age** | | | |
| --- | --- | --- | --- | --- |
|  | **Whole Sample**  **(N=10 491)** | **UMD**  **N = 5 588** | **BMD**  **N = 892** | **PSY**  **N = 4 011** |
| **Age (mean, SD)** | 25.2 (6.3) | 24.0 (6.7) | 26.8 (5.4) | 26.6 (5.4) |
| **Gender (n, %)** |  |  |  |  |
| Female | 5 941 (57) | 3 934 (70) | 605 (68) | 1 402 (35) |
| Male | 4,537 (43) | 1 643 (29) | 286 (32) | 2 608 (65) |
| Other | 11 (0.1) | 10 (0.1) | <10 (<0.1) | <10 (<0.1) |
| Missing | <10 (<0.1) | <10 (<0.1) | <10 (<0.1) | <10 (<0.1) |
| **Self-reported Ethnicity (n, %)** |  |  |  |  |
| Asian | 665 (6.3) | 330 (5.9) | 45 (5.0) | 290 (7.2) |
| Black | 3 093 (29) | 1 144 (20) | 136 (15) | 1 813 (45) |
| Mixed | 566 (5.4) | 346 (6.2) | 52 (5.8) | 168 (4.2) |
| White | 4 651 (44) | 2 802 (50) | 507 (57) | 1 342 (33) |
| Other | 688 (6.6) | 391 (7.0) | 74 (8.3) | 223 (5.6) |
| Missing | 828 (7.9) | 575 (10) | 78 (8.7) | 175 (4.4) |
| **Prescribed antidepressants (n, %)** | 3 416 (33) | 1 957 (35) | 299 (34) | 1 160 (29) |
| **Prescribed antipsychotics (n, %)** | 3 550 (34) | 385 (6.9) | 397 (45) | 2 768 (69) |
| **Prescribed anxiolytics (n, %)** | 2 058 (20) | 592 (11) | 218 (24) | 1 248 (31) |
| **Prescribed mood stabilisers (n, %)** | 993 (9.5) | 157 (2.8) | 369 (41) | 467 (12) |

|  | **Relevant medication at index excluded** | | | |
| --- | --- | --- | --- | --- |
|  | **Whole Sample**  **(N=13 021)** | **UMD**  **N = 8 121** | **BMD**  **N = 1 012** | **PSY**  **N = 3 888** |
| **Age (mean, SD)** | 41.3 (17.7) | 39.6 (18.4) | 43.3 (15.9) | 44.3 (16.3) |
| **Gender (n, %)** |  |  |  |  |
| Female | 7 488 (58) | 5 286 (65) | 607 (60) | 1 595 (41) |
| Male | 5 528 (42) | 2 831 (35) | 405 (40) | 2 292 (59) |
| Other | <10 (<0.1) | <10 (<0.1) | 0 (0) | <10 (<0.1) |
| Missing | <10 (<0.1) | <10 (<0.1) | 0 (0) | <10 (<0.1) |
| **Self-reported Ethnicity (n, %)** |  |  |  |  |
| Asian | 687 (5.3) | 398 (4.9) | 47 (4.6) | 242 (6.2) |
| Black | 2 993 (23) | 1 406 (17) | 130 (13) | 1 457 (37) |
| Mixed | 456 (3.5) | 321 (4.0) | 27 (2.7) | 108 (2.8) |
| White | 6,685 (51) | 4 410 (54) | 630 (62) | 1 645 (42) |
| Other | 764 (5.9) | 546 (6.7) | 58 (5.7) | 160 (4.1) |
| Missing | 1 436 (11) | 1 040 (13) | 120 (12) | 276 (7.1) |
| **Prescribed antidepressants (n, %)** | 387 (3) | 0 (0) | 132 (13) | 255 (6.6) |
| **Prescribed antipsychotics (n, %)** | 141 (1.1) | 141 (1.7) | 0 (0) | 0 (0) |
| **Prescribed anxiolytics (n, %)** | 380 (2.9) | 186 (2.3) | 53 (5.2) | 141 (3.6) |
| **Prescribed mood stabilisers (n, %)** | 160 (1.2) | 108 (1.3) | 0 (0) | 52 (1.3) |

**eTable** **9 Baseline sociodemographic variables stratified by inclusion in duration and first presentation analyses and SMD group. All variables refer to information at index diagnosis date. UMD: Unipolar Mood Disorders, BMD: Bipolar Disorders, PSY: Psychotic Disorders.**

|  | **Included (N=23 315)** | | | **Excluded (N=3 660)** | | |
| --- | --- | --- | --- | --- | --- | --- |
|  | **UMD**  **N = 11 445** | **BMD**  **N = 2 155** | **PSY**  **N = 9 715** | **UMD**  **N = 1 977** | **BMD**  **N = 351** | **PSY**  **N = 1 332** |
| **Age (mean, SD)** | 41.4 (18.9) | 42.6 (15.8) | 42.3 (16.2) | 39.4 (17.4) | 42.2 (15.0) | 43.2 (15.4) |
| **Gender (n, %)** |  |  |  |  |  |  |
| Female | 7,361 (64) | 1,329 (62) | 4,208 (43) | 1,282 (65) | 217 (62) | 542 (41) |
| Male | 4,072 (36) | 825 (38) | 5,503 (57) | 695 (35) | 134 (38) | 790 (59) |
| Other | 10 (<0.1) | <10 (<0.1) | <10 (<0.1) | <10 (<0.1) | <10 (<0.1) | <10 (<0.1) |
| Missing | <10 (<0.1) | <10 (<0.1) | <10 (<0.1) | <10 (<0.1) | <10 (<0.1) | <10 (<0.1) |
| **Self-reported Ethnicity (n, %)** |  |  |  |  |  |  |
| Asian | 653 (5.7) | 121 (5.6) | 715 (7.4) | 87 (4.4) | 10 (2.8) | 102 (7.7) |
| Black | 1,917 (17) | 283 (13) | 3,798 (39) | 278 (14) | 57 (16) | 509 (38) |
| Mixed | 430 (3.8) | 68 (3.2) | 280 (2.9) | 63 (3.2) | 5 (1.4) | 28 (2.1) |
| White | 6,731 (59) | 1,427 (66) | 4,125 (42) | 1,107 (56) | 216 (62) | 552 (41) |
| Other | 685 (6.0) | 120 (5.6) | 450 (4.6) | 161 (8.1) | 21 (6.0) | 59 (4.4) |
| Missing | 1,029 (9.0) | 136 (6.3) | 347 (3.6) | 281 (14) | 42 (12) | 82 (6.2) |
| **Prescribed antidepressants (n, %)** | 4,717 (41) | 788 (37) | 3,051 (31) | 584 (30) | 65 (19) | 226 (17) |
| **Prescribed antipsychotics (n, %)** | 1,081 (9.4) | 1,005 (47) | 6,561 (68) | 82 (4.1) | 99 (28) | 598 (45) |
| **Prescribed anxiolytics (n, %)** | 1,544 (13) | 584 (27) | 2,592 (27) | 154 (7.8) | 43 (12) | 184 (14) |
| **Prescribed mood stabilisers (n, %)** | 444 (3.9) | 935 (43) | 1,112 (11) | 42 (2.1) | 98 (28) | 73 (5.5) |

**eTable 10 Duration in months of prodromal period stratified by SMD group. Overall Welch comparisons, and derived Cohen’s f effect size are displayed in the fourth column. Pairwise comparisons using Wilcoxon rank sum test and derived Cohen’s d effect size and magnitude are displayed in columns 5-7. (N= 23 315) UMD: Unipolar Mood Disorders, BMD: Bipolar Mood Disorders, PSY: Psychotic Disorders.**

| **Duration of prodromal period (months)** | | | **Three-wise comparison** | **Pairwise comparisons** | | |
| --- | --- | --- | --- | --- | --- | --- |
| **UMD,**  **N = 11 445** | **BMD,**  **N = 2 155** | **PSY,**  **N = 9 715** |  | **BMD-UMD** | **PSY-BMD** | **PSY-UMD** |
| Mean (SD), Median (IQR) | Mean (SD), Median (IQR) | Mean (SD), Median (IQR) | Welch ANOVA F-value, p-value | p-value | p-value | p-value |
| 26.1 (23.9),  18 (36) | 31.1 (23.8),  26 (35) | 30.5 (24.6),  24 (39) | F(2,6 083.8)=100.24  **p-value <.0001** | **<.0001** | 0.072 | **<.0001** |
|  |  |  | Cohen’s f effect size, 95%CIs,  magnitude | Cohen’s d effect size, 95%CIs, magnitude | Cohen’s d effect size, 95%CIs,  magnitude | Cohen’s d effect size, 95%CIs,  magnitude |
|  |  |  | 0.09,  0.08-0.11,  negligible | 0.21,  0.16, 0.25,  small | -0.02,  -0.07, 0.02,  negligible | 0.18,  0.15, 0.21,  negligible |

**eTable 11 Duration in months of prodromal period for sensitivity analysis populations stratified by SMD group. Overall Welch comparisons, and derived Cohen’s f effect size are displayed in the fourth column. Pairwise comparisons using Wilcoxon rank sum test and derived Cohen’s d effect size and magnitude are displayed in columns 5-7. UMD: Unipolar Mood Disorders, BMD: Bipolar Mood Disorders, PSY: Psychotic Disorders.**

| **Duration of prodromal period (months)** | | | **Three-wise comparison** | **Pairwise comparisons** | | |
| --- | --- | --- | --- | --- | --- | --- |
| **≤35 years of age (N= 9 048/10 491)** | | | | | | |
| **UMD,**  **N = 4 712** | **BMD,**  **N = 768** | **PSY,**  **N= 3 568** |  | **BMD-UMD** | **PSY-BMD** | **PSY-UMD** |
| Mean (SD), Median (IQR) | Mean (SD), Median (IQR) | Mean (SD), Median (IQR) | Welch ANOVA F-value, p-value | p-value | p-value | p-value |
| 24.7 (23.2),  17 (36) | 27.7 (22.8),  20.5 (35) | 26.7 (23.2),  19 (36) | F(2,2154.4)=10.343,  **p-value <0.0001** | **<.0001** | 0.15 | **<.0001** |
|  |  |  | Cohen’s f effect size, 95%CIs,  magnitude | Cohen’s d effect size, 95%CIs, magnitude | Cohen’s d effect size, 95%CIs,  magnitude | Cohen’s d effect size, 95%CIs,  magnitude |
|  |  |  | 0.05,  0.03-0.7,  negligible | 0.13,  0.05, 0.20,  negligible | -0.04,  -0.12, 0.04,  negligible | 0.09,  0.04, 0.13  negligible |
| **Relevant medication at index excluded (N= 10 688/13 021)** | | | | | | |
| **UMD,**  **N = 6 728** | **BMD,**  **N = 806** | **PSY,**  **N= 3 154** |  | **BMD-UMD** | **PSY-BMD** | **PSY-UMD** |
| Mean (SD), Median (IQR) | Mean (SD), Median (IQR) | Mean (SD), Median (IQR) | Welch ANOVA F-value, p-value | p-value | p-value | p-value |
| 22.9 (21.5),  16 (33) | 25.3 (21.3),  19 (34) | 26.1 (21.4),  20 (36) | F(2,2156.4)=25.113  **p-value <0.0001** | **.0002** | 0.41 | **<.0001** |
|  |  |  | Cohen’s f effect size, 95%CIs,  Magnitude | Cohen’s d effect size, 95%CIs, magnitude | Cohen’s d effect size, 95%CIs,  magnitude | Cohen’s d effect size, 95%CIs,  magnitude |
|  |  |  | 0.07,  0.05-0.09,  negligible | 0.04,  0.04, 0.11,  small | -0.11,  -0.19, 0.04,  negligible | 0.15,  0.10, 0.19,  negligible |

**eTable 12 First prodromal clusters.**

1. **Proportion of individuals who experienced each number of first prodromal clusters, stratified by SMD group. (N= 23 315)**

| **Number of first prodromal clusters** | **UMD**  **N=11 397** | | **BMD**  **N=2 142** | | **PSY**  **N=9 674** | |
| --- | --- | --- | --- | --- | --- | --- |
|  | n | % | n | % | n | % |
| **1** | 3161 | 27.62 | 551 | 25.57 | 2930 | 30.16 |
| **2** | 1836 | 16.04 | 281 | 13.04 | 1701 | 17.51 |
| **3** | 2000 | 17.47 | 338 | 15.68 | 1320 | 13.59 |
| **4** | 1960 | 17.13 | 361 | 16.75 | 1191 | 12.26 |
| **5** | 1441 | 12.59 | 326 | 15.13 | 1201 | 12.36 |
| **6** | 801 | 7 | 219 | 10.16 | 910 | 9.37 |
| **7** | 241 | 2.11 | 78 | 3.62 | 427 | 4.4 |
| **8** | 5 | 0.04 | 1 | 0.05 | 35 | 0.36 |

1. **Proportion of individuals who experience each first prodromal cluster over the number of individuals who experienced at least one prodromal feature, stratified by SMD group. Cohen’s f effect size and magnitude derived from ANOVA model are displayed in the fifth and sixth columns. Pairwise comparisons (Cohen’s d effect sizes and magnitudes) are displayed in columns 7-12. (N= 23 315)**

|  |  |  |  | **Three-wise comparison** | | **Pairwise comparisons** | | | | | |
| --- | --- | --- | --- | --- | --- | --- | --- | --- | --- | --- | --- |
| **Cluster** | **UMD**  **N = 11 445** | **BMD**  **N = 2 155** | **PSY**  **N = 9 715** |  | | **BMD-UMD** | | **PSY-BMD** | | **PSY-UMD** | |
|  | **n (%)** | **n (%)** | **n (%)** | **Cohen’s f effect size (95%CI)** | **Magnitude** | **Cohen’s d effect size (95%CI)** | **Magnitude** | **Cohen’s d effect size (95%CI)** | **Magnitude** | **Cohen’s d effect size (95%CI)** | **Magnitude** |
| *Catatonic* | 87 (0.8) | 17 (0.8) | 242 (2.5) | **0.07**  **(0.06-0.08)** | **Negligible** | -0.03  (-0.02- -0.04) | Negligible | **0.13 (0.1-0.17)** | **Negligible** | **0.14 (0.11-0.16)** | **Negligible** |
| *Depressive* | 7 544 (66) | 1 365 (63) | 4 800 (49) | **0.16**  **(0.15-0.18)** | **Small** | -0.04  (-0.05- -0.03) | Negligible | **-0.28 (-0.33- -0.24)** | **Small** | **-0.34 (-0.37- -0.31)** | **Small** |
| *Disorganised* | 2 240 (20) | 572 (27) | 2 019 (21) | **0.05**  **(0.03-0.06)** | **Negligible** | **0.16**  **(0.15-0.18)** | **Negligible** | **-0.14 (-0.18- -0.09)** | **Negligible** | 0.03 (0-0.06) | Negligible |
| *Manic* | 5 757 (50) | 1 265 (59) | 4 371 (45) | **0.08**  **(0.07-0.09)** | **Negligible** | **0.19**  **(0.18-0.20)** | **Negligible** | **-0.28 (-0.32- -0.23)** | **Small** | **-0.11 (-0.13- -0.08)** | **Negligible** |
| *Negative* | 2 413 (21) | 416 (19) | 1 875 (19) | **0.02**  **(0.01-0.03)** | **Negligible** | -0.04  (-0.05- -0.02) | Negligible | 0 (-0.05-0.05) | Negligible | **-0.04 (-0.07- -0.02)** | **Negligible** |
| *Positive* | 3 827 (33) | 1 003 (47) | 6 140 (63) | **0.30**  **(0.28-0.31)** | **Medium** | **0.21**  **(0.19-0.22)** | **Small** | **0.34 (0.29-0.39)** | **Small** | **0.62 (0.6-0.65)** | **Medium** |
| *Substance use* | 2 496 (22) | 592 (27) | 2 782 (29) | **0.08**  **(0.06-0.09)** | **Negligible** | **0.12**  **(0.10-0.13)** | **Negligible** | 0.03 (0.02-0.07) | Negligible | **0.16 (-0.13-0.18)** | **Negligible** |
| *Other* | 10 047 (88) | 1 839 (85) | 7 561 (78) | **0.13**  **(0.12-0.14)** | **Small** | **-0.08**  **(-0.06- -0.09)** | **Negligible** | **-0.19 (-0.24- -0.15)** | **Negligible** | **-0.27 (-0.29--0.24)** | **Small** |

**C. Proportion of individuals in sensitivity analyses who experience each first prodromal cluster over the number of individuals who experienced at least one prodromal feature, stratified by SMD group. Cohen’s f effect size and magnitude derived from ANOVA model are displayed in the fifth and sixth columns. Pairwise comparisons (Cohen’s d effect sizes and magnitudes) are displayed in columns 7-12.**

|  | **≤35 years of age**  **(N=9 048/ 10 491)** | | | **Three-wise comparison** | | **Pairwise comparisons** | | | | | |
| --- | --- | --- | --- | --- | --- | --- | --- | --- | --- | --- | --- |
| **Cluster** | **UMD,**  **N=4 712** | **BMD,**  **N=768** | **PSY,**  **N=3 568** |  | | **BMD-UMD** | | **PSY-BMD** | | **PSY-UMD** | |
|  | **n (%)** | **n (%)** | **n (%)** | **Cohen’s f effect size (95%CI)** | **Magnitude** | **Cohen’s d effect size (95%CI)** | **Magnitude** | **Cohen’s d effect size (95%CI)** | **Magnitude** | **Cohen’s d effect size (95%CI)** | **Magnitude** |
| *Catatonic* | 49 (1.0) | 11 (1.4) | 136 (3.8) | **0.09 (0.07-0.11)** | **Negligible** | 0.04 (-0.04-0.12) | Negligible | **0.15 (0.08-0.22)** | **Negligible** | **0.18 (0.14-0.23)** | **Negligible** |
| *Depressive* | 3 025 (64) | 514 (67) | 2 042 (57) | **0.08 (0.05-0.1)** | **Negligible** | 0.06 (-0.02-0.13) | Negligible | **-0.2 (-0.28- -0.12)** | **Small** | **-0.14 (-0.19- -0.1)** | **Negligible** |
| *Disorganised* | 832 (18) | 225 (29) | 958 (27) | **0.12 (0.1-0.14)** | **Small** | **0.28 (0.2-0.36)** | **Small** | -0.05 (-0.13- -0.02) | Negligible | **0.22 (0.18-0.27)** | **Small** |
| *Manic* | 2 217 (47) | 469 (61) | 1 823 (51) | **0.08 (0.06-0.1)** | **Negligible** | **0.28 (0.21-0.36)** | **Small** | **-0.2 (-0.28- -0.12)** | **Small** | **0.08 (0.04-0.12)** | **Negligible** |
| *Negative* | 977 (21) | 163 (21) | 924 (26) | **0.06 (0.04-0.08)** | **Negligible** | 0.01 (-0.06-0.09) | Negligible | **0.11 (0.03-0.19)** | **Negligible** | **0.12 (0.08-0.17)** | **Negligible** |
| *Positive* | 1 544 (33) | 391 (51) | 2 350 (66) | **0.33 (0.27-0.36)** | **Medium** | **0.37 (0.29-0.45)** | **Small** | **0.31 (0.23-0.39)** | **Small** | **0.7 (0.66-0.75)** | **Medium** |
| *Substance use* | 1 138 (24) | 272 (35) | 1 463 (41) | **0.18 (0.15-0.2)** | **Small** | **0.25 (0.17-0.33)** | **Small** | **0.12 (0.04-0.19)** | **Small** | **0.37 (0.32-0.41)** | **Small** |
| *Other* | 4 165 (88) | 683 (89) | 3 022 (85) | **0.06 (0.03-0.07)** | **Negligible** | 0.02 (-0.06-0.09) | Negligible | **-0.13 (-0.2- -0.05)** | **Small** | **-0.11 (-0.15- -0.06)** | **Negligible** |

|  | **Relevant medication at index excluded (N=10 688/ 13 021)** | | | **Three-wise comparison** | | **Pairwise comparisons** | | | | | |
| --- | --- | --- | --- | --- | --- | --- | --- | --- | --- | --- | --- |
| **Cluster** | **UMD,**  **N = 6 728** | **BMD,**  **N = 806** | **PSY,**  **N= 3 154** |  | | **BMD-UMD** | | **PSY-BMD** | | **PSY-UMD** | |
| *Catatonic* | 57 (0.8) | 5 (0.6) | 76 (2.4) | **0.06 (0.04-0.08)** | **Negligible** | -0.03 (-0.1-0.04) | Negligible | 0.15 (0.08-0.21) | Negligible | **0.12 (0.08-0.17)** | **Negligible** |
| *Depressive* | 4 359 (65) | 509 (63) | 1 412 (45) | **0.19 (0.17-0.21)** | **Small** | -0.03 (-0.11-0.04) | Negligible | **-0.38 (-0.45- -0.3)** | **Small** | **-0.41 (-0.45- -0.37)** | **Small** |
| *Disorganised* | 1 264 (19) | 204 (25) | 575 (18) | 0.05 (0.03-0.06) | Negligible | **0.16 (0.08-0.23)** | **Negligible** | **-0.17 (-0.25- -0.09)** | **Negligible** | -0.01 (-0.06-0.03) | Negligible |
| *Manic* | 3 258 (48) | 466 (58) | 1 265 (40) | **0.1 (0.08-0.12)** | **Small** | **0.19 (0.12-0.26)** | **Negligible** | **-0.36 (-0.44- -0.28)** | **Small** | **-0.17 (-0.21- -0.13)** | **Negligible** |
| *Negative* | 1 333 (20) | 159 (20) | 556 (18) | 0.03 (0-0.04) | Negligible | 0 (-0.08-0.07) | Negligible | -0.05 (-0.13- -0.02) | Negligible | -0.06 (-0.1- -0.01) | Negligible |
| *Positive* | 2 116 (31) | 353 (44) | 1 970 (62) | **0.29 (0.24-0.32)** | **Medium** | **0.26 (0.18-0.33)** | **Small** | **0.38 (0.3-0.46)** | **Small** | **0.65 (0.61-0.7)** | **Medium** |
| *Substance use* | 1 380 (21) | 210 (26) | 846 (27) | **0.07 (0.05-0.09)** | **Negligible** | **0.13 (0.06-0.21)** | **Negligible** | 0.02 (-0.06-0.09) | Negligible | **0.15 (0.11-0.19)** | **Negligible** |
| *Other* | 5 892 (88) | 689 (85) | 2 371 (75) | **0.15 (0.13-0.17)** | **Small** | -0.06 (-0.14-0.01) | Negligible | **-0.26 (-0.33- -0.19)** | **Small** | **-0.32 (-0.37- -0.28)** | **Small** |

# **eTable 13 First prodromal features**

1. **Proportion of individuals who experienced each number of first prodromal features, stratified by SMD group. (N= 23 315)**

| **Number of first prodromal features** | **UMD (N=11 445)** | | **BMD (N=2 155)** | | **PSY (N=9 715)** | |
| --- | --- | --- | --- | --- | --- | --- |
|  | **n** | **%** | **n** | % | **n** | % |
| **1** | 2 766 | 24.17 | 517 | 23.99 | 2 622 | 26.99 |
| **2** | 1 802 | 15.74 | 282 | 13.09 | 1 404 | 14.45 |
| **3** | 1 459 | 12.75 | 217 | 10.07 | 1 004 | 10.33 |
| **4** | 1 172 | 10.24 | 196 | 9.1 | 782 | 8.05 |
| **5** | 1 034 | 9.03 | 162 | 7.52 | 617 | 6.35 |
| **6** | 784 | 6.85 | 152 | 7.05 | 506 | 5.21 |
| **7** | 624 | 5.45 | 129 | 5.99 | 440 | 4.53 |
| **8** | 495 | 4.33 | 91 | 4.22 | 389 | 4 |
| **9** | 356 | 3.11 | 83 | 3.85 | 309 | 3.18 |
| **10** | 262 | 2.29 | 60 | 2.78 | 239 | 2.46 |
| **11** | 184 | 1.61 | 60 | 2.78 | 229 | 2.36 |
| **12** | 132 | 1.15 | 37 | 1.72 | 192 | 1.98 |
| **13** | 105 | 0.92 | 41 | 1.9 | 180 | 1.85 |
| **14** | 69 | 0.6 | 30 | 1.39 | 124 | 1.28 |
| **15** | 64 | 0.56 | 24 | 1.11 | 148 | 1.52 |
| **16** | 42 | 0.37 | 23 | 1.07 | 106 | 1.09 |
| **17** | 23 | 0.2 | 12 | 0.56 | 78 | 0.8 |
| **18** | 17 | 0.15 | 11 | 0.51 | 86 | 0.89 |
| **19** | 15 | 0.13 | 4 | 0.19 | 55 | 0.57 |
| **20** | 14 | 0.12 | 3 | 0.14 | 52 | 0.54 |
| **21** | 6 | 0.05 | 2 | 0.09 | 40 | 0.41 |
| **22** | 6 | 0.05 | 6 | 0.28 | 36 | 0.37 |
| **23** | 4 | 0.03 | 5 | 0.23 | 26 | 0.27 |
| **24** | 1 | 0.01 | 5 | 0.23 | 21 | 0.22 |
| **25** | 3 | 0.03 | 2 | 0.09 | 6 | 0.06 |
| **27** | 2 | 0.02 | 0 | 0 | 5 | 0.05 |

# **Proportion of individuals who experienced each first prodromal feature, stratified by SMD group. (N=23 315)**

| **Feature** | **UMD (N=11 445)** | **BMD (N=2 155)** | **PSY (N=9 715)** |
| --- | --- | --- | --- |
|  | **n (%)** | **n (%)** | **n (%)** |
| Aggression | 1,048 (9.2) | 309 (14) | 1,763 (18) |
| Agitation | 1,071 (9.4) | 392 (18) | 1,833 (19) |
| Anergia | 81 (0.7) | 15 (0.7) | 37 (0.4) |
| Anhedonia | 575 (5.0) | 97 (4.5) | 261 (2.7) |
| Anxiety | 7,559 (66) | 1,364 (63) | 5,168 (53) |
| Apathy | 135 (1.2) | 25 (1.2) | 146 (1.5) |
| Arousal | 189 (1.7) | 94 (4.4) | 498 (5.1) |
| Bad dreams | 80 (0.7) | 6 (0.3) | 64 (0.7) |
| Blunted affect | 243 (2.1) | 29 (1.3) | 296 (3.0) |
| Cannabis use | 1,320 (12) | 350 (16) | 1,865 (19) |
| Circumstantial speech | 43 (0.4) | 33 (1.5) | 160 (1.6) |
| Cocaine use | 786 (6.9) | 210 (9.7) | 855 (8.8) |
| Cognitive Impairment | 6,488 (57) | 1,140 (53) | 5,197 (53) |
| Concrete thinking | 7 (<0.1) | 2 (<0.1) | 40 (0.4) |
| Delusions | 376 (3.3) | 206 (9.6) | 2,024 (21) |
| Derailment of speech | 11 (<0.1) | 11 (0.5) | 95 (1.0) |
| Disturbed sleep | 3,764 (33) | 772 (36) | 2,684 (28) |
| Diurnal mood | 100 (0.9) | 19 (0.9) | 19 (0.2) |
| Early morning wakening | 199 (1.7) | 51 (2.4) | 67 (0.7) |
| Echolalia | 3 (<0.1) | 3 (0.1) | 12 (0.1) |
| Elation | 114 (1.0) | 290 (13) | 454 (4.7) |
| Emotionally withdrawn | 525 (4.6) | 105 (4.9) | 710 (7.3) |
| Feeling helpless | 374 (3.3) | 50 (2.3) | 128 (1.3) |
| Feeling hopeless | 1,351 (12) | 198 (9.2) | 608 (6.3) |
| Feeling lonely | 659 (5.8) | 79 (3.7) | 331 (3.4) |
| Feeling worthless | 488 (4.3) | 82 (3.8) | 182 (1.9) |
| Flight of ideas | 61 (0.5) | 126 (5.8) | 246 (2.5) |
| Formal thought disorder | 14 (0.1) | 7 (0.3) | 118 (1.2) |
| Grandiosity | 48 (0.4) | 170 (7.9) | 546 (5.6) |
| Guilt | 1,204 (11) | 221 (10) | 535 (5.5) |
| Hallucinations (all) | 747 (6.5) | 214 (9.9) | 2,356 (24) |
| Hallucinations (auditory) | 347 (3.0) | 112 (5.2) | 1,722 (18) |
| Hallucinations (OTG) | 31 (0.3) | 10 (0.5) | 139 (1.4) |
| Hallucinations (visual) | 239 (2.1) | 60 (2.8) | 612 (6.3) |
| Hostility | 190 (1.7) | 84 (3.9) | 628 (6.5) |
| Insomnia | 759 (6.6) | 142 (6.6) | 428 (4.4) |
| Irritability | 957 (8.4) | 352 (16) | 1,177 (12) |
| Loss of coherence | 34 (0.3) | 35 (1.6) | 252 (2.6) |
| Low energy | 794 (6.9) | 150 (7.0) | 308 (3.2) |
| MDMA use | 74 (0.6) | 31 (1.4) | 47 (0.5) |
| Mood instability | 1,284 (11) | 485 (23) | 937 (9.6) |
| Mutism | 84 (0.7) | 14 (0.6) | 227 (2.3) |
| Negative symptom | 13 (0.1) | 3 (0.1) | 229 (2.4) |
| Nightmare | 595 (5.2) | 70 (3.2) | 308 (3.2) |
| Paranoia | 1,224 (11) | 346 (16) | 3,513 (36) |
| Passivity | 20 (0.2) | 6 (0.3) | 162 (1.7) |
| Persecutory delusions | 234 (2.0) | 88 (4.1) | 1,153 (12) |
| Poor appetite | 524 (4.6) | 99 (4.6) | 410 (4.2) |
| Poor concentration | 2,077 (18) | 424 (20) | 1,326 (14) |
| Poor insight | 1,837 (16) | 426 (20) | 1,612 (17) |
| Poor motivation | 805 (7.0) | 139 (6.5) | 447 (4.6) |
| Poverty of speech | 41 (0.4) | 12 (0.6) | 107 (1.1) |
| Poverty of thought | 10 (<0.1) | 5 (0.2) | 50 (0.5) |
| Social withdrawal | 182 (1.6) | 47 (2.2) | 237 (2.4) |
| Stupor | 1 (<0.1) | 1 (<0.1) | 13 (0.1) |
| Suicidality | 1,557 (14) | 248 (12) | 792 (8.2) |
| Tangential speech | 99 (0.9) | 71 (3.3) | 421 (4.3) |
| Tearfulness | 3,004 (26) | 427 (20) | 1,265 (13) |
| Thought block | 24 (0.2) | 9 (0.4) | 166 (1.7) |
| Thought broadcast | 18 (0.2) | 10 (0.5) | 201 (2.1) |
| Thought insertion | 19 (0.2) | 8 (0.4) | 187 (1.9) |
| Thought withdrawal | 16 (0.1) | 1 (<0.1) | 87 (0.9) |
| Tobacco use | 1,213 (11) | 269 (12) | 1,248 (13) |
| Waxy flexibility | 0 (0) | 0 (0) | 3 (<0.1) |
| Weight Loss | 941 (8.2) | 173 (8.0) | 671 (6.9) |

# **eResults 1 First presentation of prodromal clusters in sensitivity analysis populations**

When individuals over 35 were removed, the most common first presentations of prodromal clusters for UMD were: other only (27%), depressive-manic-other (9%) and depressive-other (7%); for BMD: other only (22%), depressive-disorganised-manic-positive-substance use-other (7%) and depressive-manic-other (6%); for PSY: other only (17%), depressive-disorganised-manic-negative-positive-substance use-other (8%) and positive-other (7%).

When individuals with relevant medication at index were removed, the most common first presentations of prodromal clusters for UMD were: other only (27%), depressive-manic-other (10%) and depressive-other (7%); for BMD: other only (24%), depressive-manic-other (7%) and depressive-manic-positive-other (6%); for PSY: other only (21%), positive only (14%), positive-other (10%).

# **eTable 14 Number of occurrences and normalised frequency (number of occurrences normalised by word count in EHR *10000) (mean [SD]) across prodromal period for each feature, stratified by SMD group (N=26 975).**

| **Feature** | **Number of occurrences** | | | **Normalised frequency** | | |
| --- | --- | --- | --- | --- | --- | --- |
|  | **UMD**  **N = 13 422** | **BMD**  **N = 2 506** | **PSY**  **N = 11 047** | **UMD**  **N = 13 422** | **BMD**  **N = 2 506** | **PSY**  **N = 11 047** |
|  | **Mean (SD)** | **Mean (SD)** | **Mean (SD)** | **Mean (SD)** | **Mean (SD)** | **Mean (SD)** |
|  |  |  |  |  |  |  |
| **Aggression** | 0.1 (0.8) | 0.2 (1.6) | 0.4 (2.6) | 0.3 (1.6) | 0.7 (2.9) | 1.4 (5.0) |
| **Agitation** | 0.4 (1.3) | 1.1 (2.4) | 1.5 (3.0) | 0.7 (4.3) | 1.1 (3.6) | 1.3 (4.3) |
| **Anergia** | 0.0 (0.2) | 0.0 (0.2) | 0.0 (0.2) | 0.0 (0.4) | 0.0 (0.3) | 0.0 (0.2) |
| **Anhedonia** | 0.2 (0.6) | 0.2 (0.7) | 0.1 (0.6) | 0.3 (2.1) | 0.2 (0.7) | 0.1 (0.5) |
| **Anxiety** | 33 (5.3) | 4.7 (6.7) | 4.5 (6.7) | 14.3 (23.2) | 10.9 (16.2) | 8.2 (13.6) |
| **Apathy** | 0.0 (0.2) | 0.0 (0.1) | 0.0 (0.2) | 0.0 (0.4) | 0.0 (0.3) | 0.0 (0.4) |
| **Arousal** | 0.0 (0.2) | 0.0 (0.6) | 0.1 (0.6) | 0.1 (0.4) | 0.2 (1.1) | 0.2 (1.2) |
| **Bad dreams** | 0..0 (0.2) | 0.0 (0.3) | 0.0 (0.3) | 0.0 (0.4) | 0.0 (0.3) | 0.0 (0.4) |
| **Blunted affect** | 0.1 (0.4) | 0.1 (0.6) | 0.2 (0.7) | 0.1 (0.7) | 0.1 (0.4) | 0.1 (0.5) |
| **Cannabis use** | 0.4 (1.4) | 0.8 (2.0) | 1.3 (3.4) | 1.0 (6.9) | 1.0 (3.5) | 2.0 (7.1) |
| **Circumstantial speech** | 0.0 (0.1) | 0.0 (0.1) | 0.0 (0.2) | 0.0 (0.1) | 0.0 (0.2) | 0.0 (0.3) |
| **Cocaine use** | 0.3 (1.3) | 0.5 (1.8) | 0.6 (2.6) | 0.8 (5.9) | 0.9 (7.4) | 1.0 (5.5) |
| **Cognitive impairment** | 2.6 (4.1) | 3.6 (5.3) | 4.2 (6.2) | 8.3 (14.0) | 6.3 (9.0) | 7.2 (11.1) |
| **Concrete thinking** | 0.0 (0.1) | 0.0 (0.2) | 0.0 (0.3) | 0.0 (0.1) | 0.0 (0.1) | 0.0 (0.1) |
| **Delusions** | 0.1 (0.5) | 0.4 (1.1) | 1.4 (2.6) | 0.1 (1.2) | 0.4 (2.0) | 1.6 (6.6) |
| **Derailment of speech** | 0.0 (0.0) | 0.0 (0.1) | 0.0 (0.1) | 0.0 (0.1) | 0.0 (0.1) | 0.0 (0.2) |
| **Disturbed sleep** | 1.3 (2.4) | 2.2 (3.4) | 2.0 (3.5) | 2.8 (7.1) | 2.8 (5.7) | 2.1 (4.9) |
| **Diurnal mood** | 0.0 (0.2) | 0.0 (0.3) | 0.0 (0.2) | 0.0 (0.4) | 0.0 (0.3) | 0.0 (0.1) |
| **Early morning wakening** | 0.1 (0.3) | 0.1 (0.4) | 0.0 (0.2) | 0.1 (0.6) | 0.1 (0.9) | 0.0 (0.4) |
| **Echolalia** | 0.0 (0.0) | 0.0 (0.0) | 0.0 (0.0) | 0.0 (0.0) | 0.0 (0.0) | 0.0 (0.1) |
| **Elation** | 0.0 (0.3) | 0.7 (1.5) | 0.4 (1.6) | 0.0 (0.8) | 0.9 (6.2) | 0.2 (3.2) |
| **Emotionally withdrawn** | 0.2 (0.6) | 0.3 (1.0) | 0.5 (1.3) | 0.2 (1.2) | 0.2 (0.9) | 0.3 (1.7) |
| **Feeling helpless** | 0.1 (0.5) | 0.1 (0.5) | 0.1 (0.5) | 0.2 (1.3) | 0.1 (0.6) | 0.0 (0.5) |
| **Feeling hopeless** | 0.5 (1.2) | 0.5 (1.3) | 0.4 (1.3) | 0.7 (2.6) | 0.4 (1.7) | 0.2 (1.2) |
| **Feeling lonely** | 0.3 (0.8) | 0.3 (1.0) | 0.3 (1.1) | 0.4 (2.5) | 0.2 (1.0) | 0.2 (1.7) |
| **Feeling worthless** | 0.2 (0.5) | 0.2 (0.7) | 0.1 (0.5) | 0.2 (1.1) | 0.1 (0.6) | 0.1 (0.4) |
| **Flight of ideas** | 0.0 (0.1) | 0.0 (0.4) | 0.0 (0.3) | 0.0 (0.1) | 0.1 (0.7) | 0.1 (0.7) |
| **Formal thought disorder** | 0.0 (0.0) | 0.0 (0.1) | 0.0 (0.1) | 0.0 (0.1) | 0.0 (0.1) | 0.0 (0.2) |
| **Grandiosity** | 0.0 (0.2) | 0.3 (1.1) | 0.4 (1.5) | 0.0 (0.3) | 0.4 (4.9) | 0.3 (1.6) |
| **Guilt** | 0.1 (0.5) | 0.1 (0.6) | 0.0 (0.5) | 0.3 (0.9) | 0.3 (1.0) | 0.2 (1.1) |
| **Hallucinations (all)** | 0.1 (0.6) | 0.1 (0.7) | 0.3 (1.6) | 0.2 (1.2) | 0.3 (1.3) | 1.1 (3.1) |
| **Hallucinations (auditory)** | 0.1 (0.7) | 0.3 (1.0) | 1.2 (2.5) | 0.1 (1.4) | 0.2 (1.7) | 0.4 (4.5) |
| **Hallucinations (OTG)** | 0.0 (0.2) | 0.0 (0.2) | 0.1 (0.5) | 0.2 (2.1) | 0.3 (3.3) | 1.2 (4.2) |
| **Hallucinations (visual)** | 0.1 (0.4) | 0.2 (0.7) | 0.3 (1.1) | 0.0 (0.3) | 0.0 (1.3) | 0.1 (0.7) |
| **Hostility** | 0.1 (0.4) | 0.3 (1.1) | 0.6 (1.7) | 0.1 (3.6) | 0.2 (1.0) | 0.4 (3.8) |
| **Insomnia** | 0.2 (0.7) | 0.4 (1.1) | 0.3 (0.9) | 0.4 (2.2) | 0.4 (1.4) | 0.2 (1.2) |
| **Irritability** | 0.4 (1.0) | 1.0 (2.1) | 1.1 (2.6) | 0.5 (3.4) | 0.9 (3.8) | 0.7 (2.9) |
| **Loss of coherence** | 0.0 (0.1) | 0.1 (0.3) | 0.2 (0.8) | 0.0 (0.2) | 0.0 (0.3) | 0.1 (0.8) |
| **Low energy** | 0.0 (0.3) | 0.0 (0.3) | 0.0 (0.2) | 0.1 (0.5) | 0.1 (0.5) | 0.1 (0.4) |
| **MDMA use** | 0.0 (0.2) | 0.0 (0.4) | 0.0 (0.4) | 0.0 (0.6) | 0.0 (0.3) | 0.0 (0.5) |
| **Mood instability** | 0.1 (0.5) | 0.2 (1.0) | 0.1 (0.7) | 0.3 (1.0) | 0.8 (1.7) | 0.4 (1.3) |
| **Mutism** | 0.0 (0.2) | 0.0 (0.3) | 0.2 (1.0) | 0.0 (0.8) | 0.0 (0.3) | 0.1 (1.3) |
| **Negative symptom** | 0.0 (0.1) | 0.0 (0.2) | 0.2 (0.8) | 0.0 (0.2) | 0.0 (0.1) | 0.2 (1.2) |
| **Nightmare** | 0.3 (1.0) | 0.2 (0.8) | 0.2 (1.0) | 0.4 (1.9) | 0.2 (0.9) | 0.2 (1.0) |
| **Paranoia** | 0.4 (1.2) | 0.9 (2.1) | 2.6 (4.1) | 0.7 (4.6) | 0.9 (3.0) | 3.6 (8.2) |
| **Passivity** | 0.0 (0.1) | 0.0 (0.1) | 0.1 (0.4) | 0.0 (0.1) | 0.0 (0.3) | 0.1 (0.5) |
| **Persecutory delusions** | 0.1 (0.4) | 0.2 (0.7) | 0.8 (1.7) | 0.1 (0.8) | 0.1 (0.8) | 0.6 (2.9) |
| **Poor appetite** | 0.2 (0.5) | 0.2 (0.8) | 0.3 (0.8) | 0.2 (1.5) | 0.2 (0.9) | 0.2 (1.2) |
| **Poor concentration** | 0.1 (0.5) | 0.1 (0.6) | 0.1 (0.5) | 0.4 (0.9) | 0.4 (1.0) | 0.3 (0.9) |
| **Poor insight** | 0.5 (1.2) | 1.1 (2.0) | 1.2 (2.3) | 0.7 (2.6) | 0.9 (2.0) | 0.8 (2.1) |
| **Poor motivation** | 0.3 (0.8) | 0.5 (1.2) | 0.4 (1.3) | 0.4 (2.3) | 0.4 (1.5) | 0.3 (1.6) |
| **Poverty of speech** | 0.0 (0.1) | 0.0 (0.2) | 0.1 (0.3) | 0..0 (0.2) | 0.0 (0.2) | 0.0 (0.4) |
| **Poverty of thought** | 0.0 (0.1) | 0.0 (0.1) | 0.0 (0.3) | 0.0 (0.2) | 0.0 (0.0) | 0.0 (0.3) |
| **Social withdrawal** | 0.1 (0.3) | 0.1 (0.4) | 0.1 (0.6) | 0.1 (0.7) | 0.1 (0.6) | 0.1 (0.7) |
| **Stupor** | 0.0 (0.0) | 0.0 (0.1) | 0.0 (0.2) | 0.0 (0.0) | 0.0 (0.0) | 0.0 (0.1) |
| **Suicidality** | 0.6 (1.4) | 0.7 (1.6) | 0.5 (1.6) | 1.0 (6.4) | 0.5 (1.8) | 0.4 (2.2) |
| **Tangential speech** | 0.0 (0.2) | 0.1 (0.6) | 0.3 (1.0) | 00 (0.3) | 0.1 (0.7) | 0.1 (0.8) |
| **Tearfulness** | 1.0 (2.2) | 1.3 (2.7) | 0.9 (2.4) | 1.9 (5.2) | 1.2 (2.9) | 0.7 (2.6) |
| **Thought block** | 0.0 (0.1) | 0.0 (0.2) | 0.1 (0.5) | 0.0 (0.2) | 0.0 (0.3) | 0.1 (0.9) |
| **Thought broadcasting** | 0.0 (0.1) | 0.0 (0.2) | 0.1 (0.4) | 0.0 (0.2) | 0.0 (0.3) | 0.1 (0.6) |
| **Thought insertion** | 0.0 (0.1) | 0.0 (0.2) | 0.1 (0.4) | 0.0 (0.1) | 0.0 (0.1) | 0.1 (0.5) |
| **Thought withdrawal** | 0.0 (0.1) | 0.0 (0.1) | 0.0 (0.3) | 0.0 (0.1) | 0.0 (0.0) | 0.0 (0.2) |
| **Tobacco use** | 0.4 (1.1) | 0.7 (1.8) | 1.1 (2.8) | 0.6 (3.7) | 0.5 (1.7) | 0.9 (4.5) |
| **Waxy flexibility** | 0.0 (0.0) | 0.0 (0.0) | 0.0 (0.1) | 0.0 (0.0) | 0.0 (0.0) | 0.0 (0.0) |
| **Weight loss** | 0.3 (0.8) | 0.4 (1.1) | 0.5 (1.2) | 0.4 (2.3) | 0.3 (2.5) | 0.4 (2.9) |

# **eFigure 3 Plotted model predictions for prodromal clusters** **A** Catatonic **B** Depressive **C** Disorganised **D** Manic **E** Negative **F** Positive **G** Substance use **H** Other

**
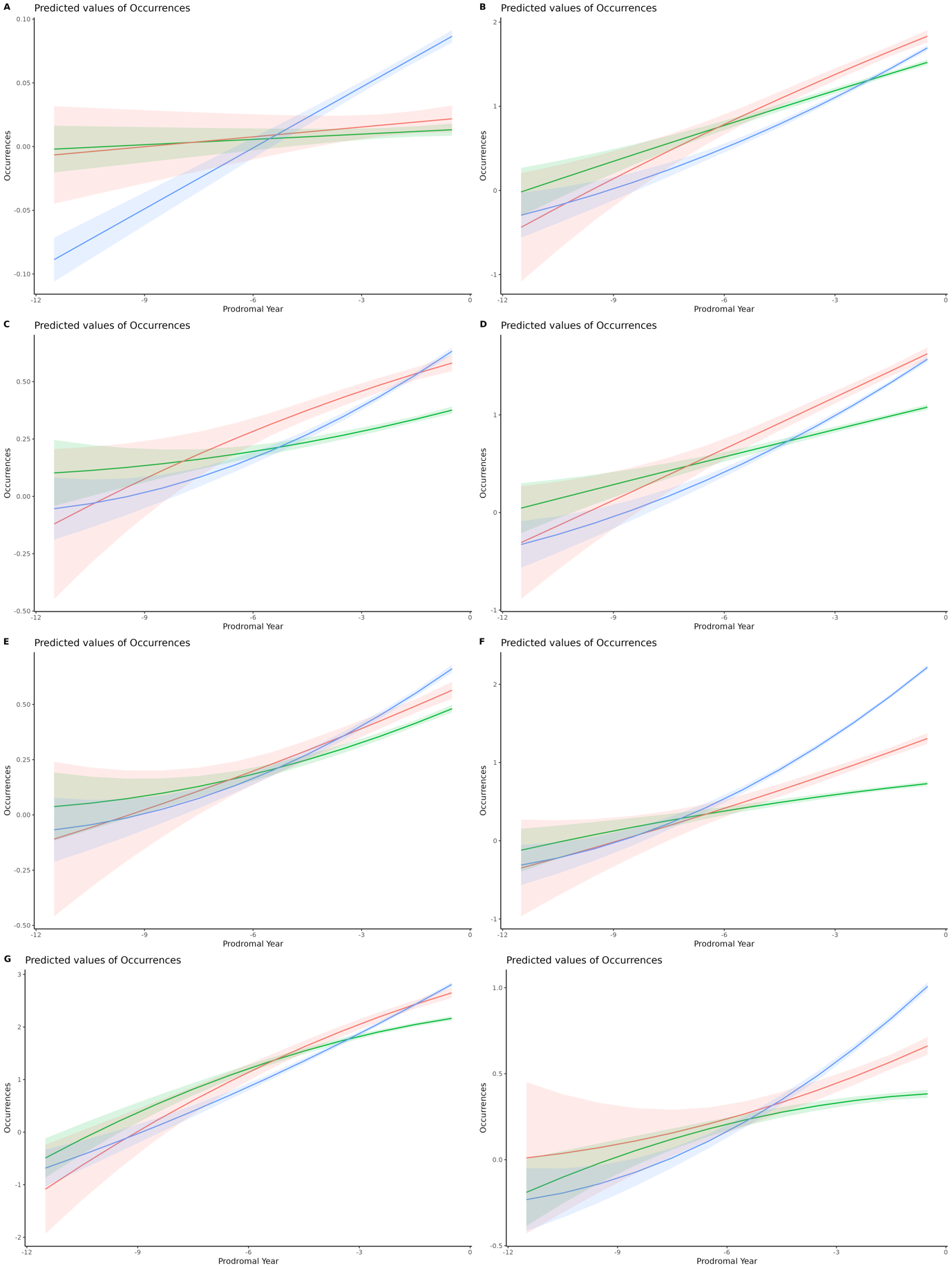
**

**eTable 15 Mixed Linear Effects Model Results. For each prodromal cluster, the coefficient (β), standard error (SE) and p-value (P-val, corrected for multiple comparisons with Benjamini Hochberg) for each fixed effects factor is shown. β indicates how much one unit of input of the predictor variable (prodromal year, SMD group, or interaction) would be expected to change the model’s output (cluster symptom occurrence).**

1. **Effect of time and interactions** (a positive β indicates a higher cluster symptom occurrence as time is closer to SMD onset. Bolded rows indicate statistically significant model terms. L, Linear; Q, Quadratic; L+I, SMD group*linear time Interaction; Q+I, SMD group*linear and quadratic time Interaction; BMD, bipolar mood disorders; PSY, psychotic disorders.

| **Model and term** | **Catatonic** | | | **Depressive** | | | **Disorganised** | | | **Manic** | | |
| --- | --- | --- | --- | --- | --- | --- | --- | --- | --- | --- | --- | --- |
|  | **β** | **SE** | **P-val** | **β** | **SE** | **P-val** | **β** | **SE** | **P-val** | **β** | **SE** | **P-val** |
| **L/**L | **0.008** | **0.001** | **<0.0001** | **0.18** | **0.004** | **<0.0001** | **0.06** | **0.0001** | **<0.0001** | **0.15** | **0.003** | **<0.0001** |
| **Q/**L | **0.009** | **0.001** | **<0.0001** | **0.17** | **0.01** | **<0.0001** | **0.05** | **0.004** | **<0.0001** | **0.14** | **0.008** | **<0.0001** |
| **Q/**Q | -0.0001 | 0.0002 | 0.55 | **0.003** | **0.001** | **0.03** | **0.002** | **0.0007** | **0.001** | **0.003** | **0.001** | **0.003** |
| **L+I/**L | 0.001 | 0.0001 | 0.74 | **0.136** | **0.006** | **<0.0001** | 0.003 | 0.001 | 0.225 | **0.093** | **0.005** | **<0.0001** |
| **L+I/**L*BMD | 0.001 | 0.002 | 0.84 | **0.05** | **0.013** | **<0.0001** | **0.021** | **0.006** | **0.002** | **0.085** | **0.012** | **<0.0001** |
| **L+I/**L*PSY | **0.015** | **0.001** | **<0.0001** | **0.080** | **0.008** | **<0.0001** | **0.052** | **0.004** | **<0.0001** | **0.117** | **0.007** | **<0.0001** |
| **Q+I/**L | 0.0007 | 0.002 | 0.91 | **0.14** | **0.01** | **<0.0001** | 0.025 | 0.007 | 0.001 | **0.09** | **0.01** | **<0.0001** |
| **Q+I**/Q | 0.0001 | 0.0003 | 0.91 | -0.0001 | 0.002 | 0.85 | 0.001 | 0.001 | 0.22 | -0.0002 | 0.002 | 0.91 |
| **Q+I/**L*BMD | 0.00006 | 0.005 | 0.91 | 0.07 | 0.03 | 0.15 | 0.039 | 0.02 | 0.08 | **0.08** | **0.035** | **0.02** |
| **Q+I/**L*PSY | **0.017** | **0.003** | **<0.0001** | 0.04 | 0.02 | 0.11 | **0.038** | **0.009** | **<0.0001** | **0.08** | **0.02** | **<0.0001** |
| **Q+I**/Q*BMD | 0.0001 | 0.0008 | 0.91 | -0.002 | 0.005 | 0.85 | -0.003 | 0.003 | 0.22 | 0.0005 | 0.004 | 0.91 |
| **Q+I/**Q*PSY | -0.0005 | 0.0005 | 0.91 | 0.01 | 0.003 | 0.08 | 0.003 | 0.001 | 0.22 | **0.007** | **0.002** | **0.02** |
| **Model and term** | **Negative** | | | **Positive** | | | **Substance use** | | | **Other** | | |
|  | **β** | **SE** | **P-val** | **β** | **SE** | **P-val** | **β** | **SE** | **P-val** | **β** | **SE** | **P-val** |
| **L/**L | **0.07** | **0.002** | **<0.0001** | **0.19** | **0.00** | **<0.0001** | **0.09** | **0.003** | **<0.0001** | **0.27** | **0.005** | **<0.0001** |
| **L+Q/**L | **0.05** | **0.005** | **<0.0001** | **0.16** | **0.008** | **<0.0001** | **0.08** | **0.01** | **<0.0001** | **0.28** | **0.01** | **<0.0001** |
| **L+Q/**Q | **0.0032** | **0.001** | **<0.0001** | **0.006** | **0.001** | **<0.0001** | **0.002** | **0.0009** | **0.02** | -0.003 | 0.002 | 0.07 |
| **L+I/**L | **0.054** | **0.003** | **<0.0001** | **0.059** | **0.005** | **<0.0001** | **0.032** | **0.004** | **<0.0001** | **0.175** | **0.008** | **<0.0001** |
| **L+I/**L*BMD | 0.013 | 0.007 | 0.082 | **0.01** | **0.01** | **<0.0001** | **0.045** | **0.009** | **<0.0001** | **0.099** | **0.011** | **<0.0001** |
| **L+I/**L*PSY | **0.036** | **0.004** | **<0.0001** | **0.24** | **0.008** | **<0.0001** | **0.121** | **0.006** | **<0.0001** | **0.179** | **0.011** | **<0.0001** |
| **Q+I/**L | **0.04** | **0.007** | **<0.0001** | **0.08** | **0.01** | **<0.0001** | **0.05** | **0.009** | **<0.0001** | **0.24** | **0.02** | **<0.0001** |
| **Q+I**/Q | 0.002 | 0.001 | 0.58 | -0.002 | 0.002 | 0.58 | 0.004 | 0.001 | 0.063 | **-0.01** | **0.003** | **<0.0001** |
| **Q+I/**L*BMD | 0.02 | 0.02 | 0.68 | 0.07 | 0.06 | 0.07 | 0.007 | 0.02 | 0.75 | 0.10 | 0.04 | 0.059 |
| **Q+I/**L*PSY | **0.03** | **0.009** | **0.048** | **0.2** | **0.03** | **<0.0001** | **0.06** | **0.01** | **<0.0001** | **0.08** | **0.02** | **0.004** |
| **Q+I**/Q*BMD | -0.002 | 0.003 | 0.68 | 0.005 | 0.004 | 0.58 | 0.007 | 0.003 | 0.21 | 0.00005 | 0.007 | 0.94 |
| **Q+I/**Q*PSY | 0.002 | 0.002 | 0.68 | **0.02** | **0.003** | **<0.0001** | **0.01** | **0.002** | **<0.0001** | **0.02** | **0.004** | **<0.0001** |

1. **Model fit** A lower AICc indicates better model fit. A higher R^2^c indicates a higher proportion of variance explained by the model. Bolded rows indicate best fitting model.

| **Model** | **Catatonic** | | | **Depressive** | | | **Disorganised** | | | **Manic** | | |
| --- | --- | --- | --- | --- | --- | --- | --- | --- | --- | --- | --- | --- |
|  | **AICc** | **ΔAICc** | **R^2^c** | **AICc** | **ΔAICc** | **R^2^c** | **AICc** | **ΔAICc** | **R^2^c** | **AICc** | **ΔAICc** | **R^2^c** |
| Q+I | 21988 | 5 | 0.19 | **255122** | **0** | **0.31** | **165249** | **0** | **0.21** | **234459** | **0** | **0.29** |
| L+I | **21983** | **0** | **0.19** | 255127 | 5 | 0.31 | 165261 | 12 | 0.21 | 234471 | 12 | 0.29 |
| Q | 22111 | 128 | 0.19 | 255219 | 97 | 0.31 | 165411 | 162 | 0.20 | 234739 | 280 | 0.29 |
| L | 22109 | 126 | 0.19 | 255221 | 99 | 0.31 | 165420 | 171 | 0.20 | 234746 | 287 | 0.29 |
| **Yr*PSY** | **Negative** | | | **Positive** | | | **Substance use** | | | **Other** | | |
|  | **AICc** | **ΔAICc** | **R^2^c** | **AICc** | **ΔAICc** | **R^2^c** | **AICc** | **ΔAICc** | **R^2^c** | **AICc** | **ΔAICc** | **R^2^c** |
| Q+I | **174937** | **0** | **0.24** | **244890** | **0** | **0.35** | **208339** | **0** | **0.37** | **290892** | **0** | **0.35** |
| L+I | 174952 | 15 | 0.24 | 248938 | 48 | 0.35 | 208370 | 31 | 0.37 | 290915 | 23 | 0.35 |
| Q | 175002 | 65 | 0.24 | 249883 | 993 | 0.34 | 208831 | 492 | 0.36 | 291189 | 297 | 0.34 |
| L | 175018 | 81 | 0.24 | 249898 | 1008 | 0.34 | 208835 | 495 | 0.36 | 291190 | 298 | 0.34 |

**(C) Final model coefficients** Full model terms for best fitting model per cluster. P-values are corrected using the Benjamini-Holm procedure. Bolded rows indicate statistically significant model terms. UMD was the reference category for SMD group. BMD, bipolar mood disorders; PSY, psychotic disorders.

| **Model and term** | **Catatonic** | | | **Depressive** | | | **Disorganised** | | | **Manic** | | |
| --- | --- | --- | --- | --- | --- | --- | --- | --- | --- | --- | --- | --- |
|  | **β** | **SE** | **P-val** | **β** | **SE** | **P-val** | **β** | **SE** | **P-val** | **β** | **SE** | **P-val** |
| (Intercept) | 0.006 | 0.004 | 0.793 | **0.772** | **0.027** | **<0.0001** | **0.20** | **0.014** | **<0.0001** | **0.498** | **0.024** | **<0.0001** |
| Time | 0.001 | 0.001 | 0.740 | **0.140** | **0.012** | **<0.0001** | **0.025** | **0.007** | **0.001** | **0.086** | **0.011** | **<0.0001** |
| BMD | 0.002 | 0.01 | 0.841 | 0.01 | 0.063 | 0.853 | **0.088** | **0.032** | **0.037** | 0.073 | 0.056 | 0.509 |
| PSY | -0.007 | 0.006 | 0.793 | **-0.270** | **0.037** | **<0.0001** | -0.028 | 0.019 | 0.224 | **-0.142** | **0.033** | **<0.0001** |
| Time^2^ | NA | NA | NA | -0.001 | 0.002 | 0.853 | 0.001 | 0.001 | 0.224 | 0 | 0.002 | 0.907 |
| Time*BMD | 0.001 | 0.002 | 0.841 | 0.066 | 0.03 | 0.151 | 0.039 | 0.016 | 0.082 | **0.076** | **0.027** | **0.021** |
| Time*PSY | **0.015** | **0.001** | **<0.0001** | 0.041 | 0.017 | 0.110 | **0.038** | **0.009** | **<0.0001** | **0.075** | **0.015** | **<0.0001** |
| Time^2^*BMD | NA | NA | NA | -0.002 | 0.005 | 0.852 | -0.003 | 0.003 | 0.224 | 0.002 | 0.004 | 0.907 |
| Time^2^*PSY | NA | NA | NA | 0.007 | 0.003 | 0.08 | 0.003 | 0.001 | 0.224 | **0.007** | **0.002** | **0.024** |
| **Model and term** | **Negative** | | | **Positive** | | | **Substance use** | | | **Other** | | |
|  | **β** | **SE** | **P-val** | **β** | **SE** | **P-val** | **β** | **SE** | **P-val** | **β** | **SE** | **P-val** |
| (Intercept) | **0.184** | **0.015** | **<0.0001** | **0.380** | **0.027** | **<0.0001** | **0.207** | **0.02** | **<0.0001** | **1.208** | **0.038** | **<0.0001** |
| Time | **0.040** | **0.007** | **<0.0001** | **0.077** | **0.012** | **<0.0001** | **0.052** | **0.009** | **<0.0001** | **0.241** | **0.017** | **<0.0001** |
| BMD | 0.015 | 0.035 | 0.678 | 0.034 | 0.063 | 0.584 | 0.03 | 0.046 | 0.745 | -0.069 | 0.088 | 0.864 |
| PSY | -0.019 | 0.02 | 0.678 | **0.154** | **0.037** | **0.0001** | -0.043 | 0.027 | 0.332 | **-0.346** | **0.052** | **<0.0001** |
| Time^2^ | 0.002 | 0.001 | 0.183 | -0.002 | 0.002 | 0.584 | -0.004 | 0.001 | 0.063 | **-0.012** | **0.003** | **<0.0001** |
| Time*BMD | 0.02 | 0.017 | 0.678 | 0.073 | 0.031 | 0.068 | 0.007 | 0.022 | 0.745 | 0.098 | 0.042 | 0.059 |
| Time*PSY | **0.026** | **0.009** | **0.048** | **0.153** | **0.017** | **<0.0001** | **0.061** | **0.012** | **<0.0001** | **0.076** | **0.023** | **0.004** |
| Time^2^*BMD | -0.002 | 0.003 | 0.678 | 0.005 | 0.005 | 0.584 | 0.007 | 0.004 | 0.205 | 0.0005 | 0.007 | 0.941 |
| Time^2^*PSY | 0.002 | 0.002 | 0.678 | **0.016** | **0.003** | **<0.0001** | **0.011** | **0.002** | **<0.0001** | **0.019** | **0.004** | **<0.0001** |

1. **Model fit** Sensitivity analyses investigating model fit in individuals ≤35 years of age and with relevant medication at index excluded. A lower AICc indicates better model fit. A higher R^2^c indicates a higher proportion of variance explained by the model. Bolded rows indicate best fitting model.

|  | **≤35 years of age** | | | | | | | | | | | |
| --- | --- | --- | --- | --- | --- | --- | --- | --- | --- | --- | --- | --- |
| **Model** | **Catatonic** | | | **Depressive** | | | **Disorganised** | | | **Manic** | | |
|  | **AICc** | **ΔAICc** | **R^2^c** | **AICc** | **ΔAICc** | **R^2^c** | **AICc** | **ΔAICc** | **R^2^c** | **AICc** | **ΔAICc** | **R^2^c** |
| Q+I | 16410 | 5 | 0.19 | **92790** | **0** | **0.30** | **628780** | **0** | **0.21** | **87128** | **0** | **0.30** |
| L+I | **16405** | **0** | **0.20** | 92802 | 11 | 0.30 | 62889 | 9 | 0.21 | 87137 | 9 | 0.30 |
| Q | 16476 | 72 | 0.19 | 92862 | 72 | 0.30 | 62983 | 103 | 0.21 | 87270 | 142 | 0.29 |
| L | 16474 | 70 | 0.19 | 92866 | 76 | 0.30 | 62991 | 111 | 0.21 | 87275 | 147 | 0.29 |
| **Yr*PSY** | **Negative** | | | **Positive** | | | **Substance use** | | | **Other** | | |
|  | **AICc** | **ΔAICc** | **R^2^c** | **AICc** | **ΔAICc** | **R^2^c** | **AICc** | **ΔAICc** | **R^2^c** | **AICc** | **ΔAICc** | **R^2^c** |
| Q+I | **67675** | **0** | **0.25** | **89638** | **0** | **0.37** | **79735** | **0** | **0.38** | **105461** | **0** | **0.33** |
| L+I | 67685 | 9 | 0.25 | 89666 | 28 | 0.37 | 79744 | 9 | 0.38 | 105473 | 12 | 0.33 |
| Q | 67734 | 59 | 0.25 | 90108 | 470 | 0.35 | 79970 | 235 | 0.37 | 105657 | 197 | 0.32 |
| L | 67744 | 69 | 0.25 | 90118 | 480 | 0.35 | 79971 | 236 | 0.37 | 105659 | 198 | 0.32 |
|  | **Relevant medication at index excluded** | | | | | | | | | | | |
| **Model** | **Catatonic** | | | **Depressive** | | | **Disorganised** | | | **Manic** | | |
|  | **AICc** | **ΔAICc** | **R^2^c** | **AICc** | **ΔAICc** | **R^2^c** | **AICc** | **ΔAICc** | **R^2^c** | **AICc** | **ΔAICc** | **R^2^c** |
| Q+I | -23255 | 2 | 0.22 | 93615 | 4 | 0.25 | 52092 | 5 | 0.16 | 84953 | 3 | 0.23 |
| L+I | **-23257** | **0** | **0.22** | **93610** | **0** | **0.25** | **52087** | **0** | **0.16** | **83949** | **0** | **0.23** |
| Q | -23254 | 3 | 0.22 | 93622 | 12 | 0.25 | 52110 | 22 | 0.16 | 84994 | 44 | 0.23 |
| L | -23254 | 3 | 0.22 | 93622 | 10 | 0.25 | 52109 | 21 | 0.16 | 84992 | 43 | 0.23 |
| **Yr*PSY** | **Negative** | | | **Positive** | | | **Substance use** | | | **Other** | | |
|  | **AICc** | **ΔAICc** | **R^2^c** | **AICc** | **ΔAICc** | **R^2^c** | **AICc** | **ΔAICc** | **R^2^c** | **AICc** | **ΔAICc** | **R^2^c** |
| Q+I | 56598 | 5 | 0.19 | 87387 | 1 | 0.29 | 27151 | 4 | 0.35 | **111004** | **0** | **0.28** |
| L+I | 56593 | 0 | 0.19 | **87386** | **0** | **0.30** | **27147** | **0** | **0.35** | 111010 | 7 | 0.28 |
| Q | 56599 | 6 | 0.19 | 87647 | 261 | 0.29 | 27156 | 9 | 0.35 | 111087 | 84 | 0.27 |
| L | **56594** | **4** | **0.19** | 87646 | 259 | 0.29 | 27155 | 8 | 0.35 | 111092 | 88 | 0.27 |

1. **Final model coefficients** Full model terms for best fitting model per cluster. P-values are corrected using the Benjamini-Holm procedure. Bolded rows indicate statistically significant model terms. UMD was the reference category for SMD group. BMD, bipolar mood disorders; PSY, psychotic disorders.

|  | **≤35 years of age** | | | | | | | | | | | |
| --- | --- | --- | --- | --- | --- | --- | --- | --- | --- | --- | --- | --- |
| **Model and term** | **Catatonic** | | | **Depressive** | | | **Disorganised** | | | **Manic** | | |
|  | **β** | **SE** | **P-val** | **β** | **SE** | **P-val** | **β** | **SE** | **P-val** | **β** | **SE** | **P-val** |
| (Intercept) | 0.009 | 0.009 | 0.957 | **0.736** | **0.051** | **<0.0001** | **0.019** | **0.026** | **<0.0001** | **0.526** | **0.044** | **<0.0001** |
| Time | 0.002 | 0.002 | 0.957 | **0.138** | **0.025** | **<0.0001** | 0.002 | 0.013 | 0.437 | **0.088** | **0.022** | **<0.0001** |
| BMD | 0.001 | 0.023 | 0.957 | 0.027 | 0.133 | 0.840 | 0.005 | 0.068 | 0.437 | 0.070 | 0.12 | 0.550 |
| PSY | -0.010 | 0.013 | 0.957 | -0.129 | 0.075 | 0.441 | 0.045 | 0.038 | 0.437 | -0.045 | 0.066 | 0.550 |
| Time^2^ | NA | NA | NA | 0.002 | 0.004 | 0.840 | 0.003 | 0.002 | 0.437 | 0.002 | 0.004 | 0.550 |
| Time*BMD | 0.003 | 0.005 | 0.957 | 0.161 | 0.068 | 0.108 | 0.080 | 0.036 | 0.176 | 0.157 | 0.060 | 0.053 |
| Time*PSY | **0.024** | **0.003** | **0.001** | 0.039 | 0.037 | 0.840 | 0.051 | 0.020 | 0.080 | 0.087 | 0.033 | 0.053 |
| Time^2^*BMD | NA | NA | NA | -0.016 | 0.011 | 0.576 | -0.006 | 0.006 | 0.437 | -0.009 | 0.009 | 0.550 |
| Time^2^*PSY | NA | NA | NA | **0.015** | **0.006** | **0.077** | 0.005 | 0.003 | 0.437 | 0.013 | 0.005 | 0.083 |
| **Model and term** | **Negative** | | | **Positive** | | | **Substance use** | | | **Other** | | |
|  | **β** | **SE** | **P-val** | **β** | **SE** | **P-val** | **β** | **SE** | **P-val** | **β** | **SE** | **P-val** |
| (Intercept) | **0.158** | **0.03** | **<0.0001** | **0.401** | **0.048** | **<0.0001** | **0.173** | **0.038** | **<0.0001** | **1.391** | **0.067** | **<0.0001** |
| Time | **0.045** | **0.01** | **0.006** | **0.064** | **0.023** | **0.038** | **0.071** | **0.018** | **0.001** | **0.229** | **0.033** | **<0.0001** |
| BMD | -0.018 | 0.08 | 0.949 | 0.008 | 0.124 | 0.951 | 0.162 | 0.100 | 0.527 | -0.043 | 0.177 | 0.809 |
| PSY | **0.053** | **0.04** | **0.046** | 0.140 | 0.070 | 0.185 | 0.085 | 0.057 | 0.527 | **-0.391** | **0.100** | **0.0007** |
| Time^2^ | 0.004 | 0.002 | 0.951 | -0.0002 | 0.004 | 0.951 | -0.003 | 0.003 | 0.735 | **-0.015** | **0.005** | **0.021** |
| Time*BMD | **0.060** | **0.04** | **0.012** | 0.158 | 0.063 | 0.062 | 0.017 | 0.050 | 0.735 | 0.166 | 0.089 | 0.191 |
| Time*PSY | **0.040** | **0.02** | **<0.0001** | **0.166** | **0.035** | **<0.0001** | **0.082** | **0.028** | **0.018** | 0.105 | 0.049 | 0.133 |
| Time^2^*BMD | -0.006 | 0.006 | 0.516 | -0.006 | 0.010 | 0.951 | 0.003 | 0.008 | 0.735 | -0.008 | 0.014 | 0.809 |
| Time^2^*PSY | **0.005** | **0.003** | **<0.0001** | **0.024** | **0.006** | **<0.0001** | **0.015** | **0.004** | **0.003** | **0.030** | **0.008** | **0.0007** |

|  | **Relevant medication at index excluded** | | | | | | | | | | | |
| --- | --- | --- | --- | --- | --- | --- | --- | --- | --- | --- | --- | --- |
| **Model and term** | **Catatonic** | | | **Depressive** | | | **Disorganised** | | | **Manic** | | |
|  | **β** | **SE** | **P-val** | **β** | **SE** | **P-val** | **β** | **SE** | **P-val** | **β** | **SE** | **P-val** |
| (Intercept) | 0.009 | 0.004 | 0.052 | **0.776** | **0.033** | **<0.0001** | **0.190** | **0.015** | **<0.0001** | **0.562** | **0.028** | **<0.0001** |
| Time | -0.0002 | 0.001 | 0.841 | **0.081** | **0.007** | **<0.0001** | **0.017** | **0.003** | **<0.0001** | **0.049** | **0.006** | **<0.0001** |
| BMD | -0.009 | 0.010 | 0.718 | -0.062 | 0.096 | 0.517 | **0.098** | **0.043** | **0.045** | 0.122 | 0.081 | 0.257 |
| PSY | 0.010 | 0.006 | 0.408 | **-0.380** | **0.055** | **<0.0001** | **-0.060** | **0.024** | **0.041** | **-0.233** | **0.046** | **<0.0001** |
| Time^2^ | NA | NA | NA | NA | NA | NA | NA | NA | NA | NA | NA | NA |
| Time*BMD | 0.002 | 0.002 | 0.718 | 0.019 | 0.021 | 0.517 | -0.003 | 0.009 | 0.783 | 0.020 | 0.018 | 0.257 |
| Time*PSY | 0.003 | 0.001 | 0.103 | **0.044** | **0.012** | **0.0005** | **0.026** | **0.005** | **<0.0001** | **0.069** | **0.010** | **<0.0001** |
| Time^2^*BMD | NA | NA | NA | NA | NA | NA | NA | NA | NA | NA | NA | NA |
| Time^2^*PSY | NA | NA | NA | NA | NA | NA | NA | NA | NA | NA | NA | NA |
| **Model and term** | **Negative** | | | **Positive** | | | **Substance use** | | | **Other** | | |
|  | **β** | **SE** | **P-val** | **β** | **SE** | **P-val** | **β** | **SE** | **P-val** | **β** | **SE** | **P-val** |
| (Intercept) | **0.190** | **0.016** | **<0.0001** | **0.393** | **0.030** | **<0.0001** | **0.196** | **0.037** | **<0.0001** | **1.200** | **0.051** | **<0.0001** |
| Time | **0.028** | **0.004** | **<0.0001** | **0.029** | **0.006** | **<0.0001** | **0.029** | **0.008** | **0.001** | **0.172** | **0.027** | **<0.0001** |
| BMD | -0.009 | 0.047 | 0.845 | 0.086 | 0.085 | 0.313 | 0.179 | 0.135 | 0.373 | -0.087 | 0.163 | 0.733 |
| PSY | -0.035 | 0.027 | 0.577 | 0.056 | 0.056 | 0.313 | **0.251** | **0.074** | **0.003** | **-0.688** | **0.096** | **<0.0001** |
| Time^2^ | NA | NA | NA | NA | NA | NA | NA | NA | NA | **-0.012** | **0.004** | **0.022** |
| Time*BMD | -0.005 | 0.010 | 0.845 | 0.025 | 0.025 | 0.313 | -0.009 | 0.028 | 0.743 | -0.031 | 0.092 | 0.733 |
| Time*PSY | **0.017** | **0.006** | **0.016** | **0.170** | **0.170** | **<0.0001** | **0.051** | **0.015** | **0.003** | **0.180** | **0.055** | **0.006** |
| Time^2^*BMD | NA | NA | NA | NA | NA | NA | NA | NA | NA | 0.012 | 0.014 | 0.733 |
| Time^2^*PSY | NA | NA | NA | NA | NA | NA | NA | NA | NA | -0.003 | 0.008 | 0.733 |

# **eTable 16 Near-zero and non-near-zero variance features**

A feature is considered to have near-zero variance if it meets the following 2 requirements:

(a) a large (> 20) frequency ratio (frequency of most prevalent number of occurrences to the frequency of the second most prevalent number of occurrences)

(b) the fraction of unique values of occurrences over the sample size is low (<10%) across the duration of the prodrome

The % of absent values is displayed in column 3 for reference.

**Near-zero variance features (N=28)**

| **Feature** | **% unique values** | **% absent values** | **Frequency Ratio** |
| --- | --- | --- | --- |
| Anergia | 0.03 | 97.92 | 57.3 |
| Apathy | 0.05 | 95.23 | 25.87 |
| Bad dreams | 0.04 | 97.14 | 42.4 |
| Circumstantial speech | 0.04 | 96 | 34.03 |
| Concrete thinking | 0.03 | 98.71 | 97.89 |
| Derailment of speech | 0.03 | 98.18 | 69.33 |
| Diurnal mood | 0.03 | 98.06 | 62.09 |
| Early morning wakening | 0.03 | 96.14 | 30.66 |
| Echolalia | 0.02 | 99.6 | 288.88 |
| Flight of ideas | 0.07 | 94.05 | 25.02 |
| Formal thought disorder | 0.03 | 97.94 | 62.02 |
| Grandiosity | 0.1 | 91.63 | 19.92 |
| Hallucinations (OTG) | 0.05 | 97.21 | 52.76 |
| Loss of coherence | 0.06 | 94.78 | 26.83 |
| MDMA use | 0.04 | 98.51 | 102.6 |
| Mutism | 0.07 | 95.89 | 35.78 |
| Negative symptoms | 0.05 | 95.22 | 31.29 |
| Passivity | 0.04 | 97.31 | 51.88 |
| Poverty of speech | 0.03 | 97.81 | 57.73 |
| Poverty of thought | 0.03 | 98.85 | 105.81 |
| Social withdrawal | 0.04 | 93.55 | 19.76 |
| Stupor | 0.03 | 99.76 | 517.48 |
| Tangential speech | 0.06 | 92.37 | 20 |
| Thought block | 0.04 | 97.04 | 43.99 |
| Thought broadcast | 0.04 | 96.89 | 41.62 |
| Thought insert | 0.04 | 96.78 | 40.47 |
| Thought withdrawal | 0.03 | 98.41 | 76.5 |
| Waxy flexibility | 0.02 | 99.93 | 1797.07 |

**Non-near-zero variance features (N=37)**

| **Feature** | **% unique values** | **% absent values** | **Frequency Ratio** |
| --- | --- | --- | --- |
| Aggression | 0.14 | 69.89 | 4.71 |
| Agitation | 0.14 | 67.18 | 4.32 |
| Anhedonia | 0.04 | 88.85 | 11.29 |
| Anxiety | 0.23 | 26.66 | 1.29 |
| Arousal | 0.1 | 88.14 | 12.59 |
| Blunted flat affect | 0.05 | 91.15 | 14.87 |
| Cannabis use | 0.15 | 75.21 | 6.61 |
| Cocaine use | 0.16 | 85.97 | 12.21 |
| Cognitive impairment | 0.22 | 29.21 | 1.34 |
| Delusional ideation | 0.13 | 75.35 | 6.33 |
| Disturbed sleep | 0.14 | 46.9 | 2.2 |
| Elation | 0.1 | 89.05 | 15.01 |
| Emotionally withdrawn | 0.08 | 82.73 | 7.6 |
| Feeling helpless | 0.05 | 91.62 | 14.66 |
| Feeling hopeless | 0.08 | 77.27 | 5.93 |
| Feeling lonely | 0.07 | 84.7 | 8.95 |
| Feeling worthless | 0.04 | 90.7 | 13.53 |
| Guilt | 0.09 | 78.06 | 5.95 |
| Hallucinations (all) | 0.13 | 70.74 | 5.44 |
| Hallucination (auditory) | 0.1 | 78.31 | 7.72 |
| Hallucination (visual) | 0.07 | 89.55 | 13.78 |
| Hostility | 0.1 | 86.51 | 11.43 |
| Insomnia | 0.06 | 83.86 | 8.17 |
| Irritability | 0.13 | 71.64 | 4.98 |
| Low energy | 0.05 | 84.33 | 8.08 |
| Mood instability | 0.12 | 69.91 | 4.42 |
| Nightmare | 0.08 | 88.1 | 12.41 |
| Paranoia | 0.15 | 60.96 | 3.9 |
| Persecutory delusions | 0.08 | 82.69 | 8.69 |
| Poor appetite | 0.06 | 85.81 | 8.35 |
| Poor concentration | 0.09 | 64.5 | 3.45 |
| Poor insight | 0.11 | 62.94 | 3.25 |
| Poor motivation | 0.07 | 79.84 | 6.65 |
| Suicidality | 0.1 | 74.65 | 5.39 |
| Tearfulness | 0.14 | 62.94 | 3.62 |
| Tobacco use | 0.15 | 73.64 | 5.46 |
| Weight loss | 0.08 | 79.55 | 6.27 |

**eTable 17 Discriminability scores for feature occurrences. (N=26 975).**

|  | **Pair-wise discriminability scores** | | | | | | **Three-wise discriminability scores** | |
| --- | --- | --- | --- | --- | --- | --- | --- | --- |
|  | **BMD-UMD** | | **BMD-PSY** | | **UMD-PSY** | |  |  |
|  | **Cohen’s d effect size (95%CI)** | **Magnitude** | **Cohen’s d effect size (95%CI)** | **Magnitude** | **Cohen’s d effect size (95%CI)** | **Magnitude** | **Cohen’s f effect size (95%CI)** | **Magnitude** |
| **Aggression** | 0.26 (0.21-0.31) | Small | -0.19 (-0.23--0.15) | Negligible | -0.4 (-0.43--0.37) | Small | 0.2 (0.16-0.21) | Small |
| **Agitation** | 0.35 (0.3-0.4) | Small | -0.12 (-0.16--0.08) | Negligible | -0.43 (-0.46--0.41) | Small | 0.21 (0.2-0.23) | Small |
| **Anergia** | 0.06 (0.02-0.11) | Negligible | 0.08 (0.03-0.12) | Negligible | 0.02 (-0.01-0.04) | Negligible | 0.02 (0.01-0.03) | Negligible |
| **Anhedonia** | 0.08 (0.04-0.13) | Negligible | 0.12 (0.07-0.16) | Negligible | 0.04 (0.01-0.06) | Negligible | 0.04 (0.02-0.05) | Negligible |
| **Anxiety** | 0.23 (0.18-0.27) | Small | 0.03 (-0.01-0.08) | Negligible | -0.19 (-0.21--0.16) | Negligible | 0.1 (0.09-0.11) | Small |
| **Apathy** | 0.1 (0.05-0.15) | Negligible | -0.05 (-0.09--0.01) | Negligible | -0.14 (-0.17--0.12) | Negligible | 0.07 (0.06-0.08) | Negligible |
| **Arousal** | 0.24 (0.19-0.29) | Small | -0.13 (-0.17--0.1) | Negligible | -0.32 (-0.34--0.29) | Small | 0.16 (0.15-0.17) | Small |
| **Bad dreams** | -0.01 (-0.05-0.04) | Negligible | -0.04 (-0.09-0) | Negligible | -0.04 (-0.07--0.02) | Negligible | 0.02 (0.01-0.03) | Negligible |
| **Blunted affect** | 0.07 (0.02-0.11) | Negligible | -0.13 (-0.17--0.09) | Negligible | -0.2 (-0.23--0.18) | Small | 0.1 (0.09-0.11) | Small |
| **Cannabis use** | 0.2 (0.16-0.25) | Small | -0.2 (-0.23--0.16) | Small | -0.35 (-0.37--0.32) | Small | 0.05 (0.04-0.07) | Negligible |
| **Circumstantial speech** | 0.21 (0.15-0.26) | Small | -0.1 (-0.14--0.06) | Negligible | -0.28 (-0.3--0.25) | Small | 0.14 (0.12-0.15) | Small |
| **Cocaine use** | 0.15 (0.1-0.19) | Negligible | -0.07 (-0.11--0.03) | Negligible | -0.19 (-0.21--0.16) | Negligible | 0.17 (0.14-0.19) | Small |
| **Cognitive impairment** | 0.22 (0.18-0.27) | Small | -0.1 (-0.14--0.06) | Negligible | -0.31 (-0.33--0.28) | Small | 0.15 (0.14-0.16) | Small |
| **Concrete thinking** | 0.05 (0-0.1) | Negligible | -0.08 (-0.12--0.05) | Negligible | -0.13 (-0.16--0.11) | Negligible | 0.07 (0.05-0.08) | Negligible |
| **Delusions** | 0.36 (0.31-0.41) | Small | -0.47 (-0.51--0.44) | Small | -0.66 (-0.69--0.64) | Medium | 0.34 (0.33-0.36) | Medium |
| **Derailment of speech** | 0.13 (0.08-0.18) | Negligible | -0.12 (-0.15--0.08) | Negligible | -0.21 (-0.24--0.19) | Small | 0.11 (0.09-0.12) | Small |
| **Disturbed sleep** | 0.3 (0.25-0.35) | Small | 0.06 (0.02-0.1) | Negligible | -0.23 (-0.25--0.2) | Small | 0.12 (0.11-0.13) | Small |
| **Diurnal mood** | 0.08 (0.03-0.13) | Negligible | 0.13 (0.08-0.19) | Negligible | 0.06 (0.04-0.09) | Negligible | 0.05 (0.04-0.06) | Negligible |
| **Early morning wakening** | 0.1 (0.06-0.15) | Negligible | 0.16 (0.11-0.21) | Negligible | 0.07 (0.04-0.09) | Negligible | 0.05 (0.04-0.07) | Negligible |
| **Echolalia** | 0.05 (0-0.1) | Negligible | -0.05 (-0.08--0.01) | Negligible | -0.08 (-0.11--0.05) | Negligible | 0.04 (0.03-0.05) | Negligible |
| **Elation** | 0.65 (0.59-0.71) | Medium | 0.19 (0.15-0.24) | Negligible | -0.33 (-0.36--0.31) | Small | 0.21 (0.19-0.22) | Small |
| **Emotionally withdrawn** | 0.14 (0.09-0.19) | Negligible | -0.15 (-0.19--0.11) | Negligible | -0.27 (-0.3--0.25) | Small | 0.13 (0.12-0.15) | Small |
| **Feeling helpless** | 0 (-0.05-0.04) | Negligible | 0.05 (0.01-0.09) | Negligible | 0.05 (0.03-0.08) | Negligible | 0.03 (0.01-0.04) | Negligible |
| **Feeling hopeless** | 0.06 (0.02-0.1) | Negligible | 0.1 (0.06-0.15) | Negligible | 0.05 (0.02-0.07) | Negligible | 0.03 (0.02-0.04) | Negligible |
| **Feeling lonely** | 0.03 (-0.02-0.07) | Negligible | -0.04 (-0.08-0) | Negligible | -0.07 (-0.09--0.04) | Negligible | 0.03 (0.02-0.04) | Negligible |
| **Feeling worthless** | 0.09 (0.04-0.13) | Negligible | 0.16 (0.11-0.2) | Negligible | 0.08 (0.06-0.11) | Negligible | 0.08 (0.07-0.1) | Negligible |
| **Flight of ideas** | 0.45 (0.4-0.51) | Small | 0.08 (0.04-0.13) | Negligible | -0.28 (-0.3--0.25) | Small | 0.16 (0.14-0.17) | Small |
| **Formal thought disorder** | 0.1 (0.05-0.15) | Negligible | -0.14 (-0.18--0.11) | Negligible | -0.23 (-0.25--0.2) | Small | 0.12 (0.1-0.13) | Small |
| **Grandiosity** | 0.42 (0.36-0.48) | Small | -0.04 (-0.08-0) | Negligible | -0.35 (-0.38--0.33) | Small | 0.18 (0.15-0.19) | Small |
| **Guilt** | 0.11 (0.06-0.15) | Negligible | 0.13 (0.09-0.18) | Negligible | 0.04 (0.01-0.06) | Negligible | 0.04 (0.03-0.06) | Negligible |
| **Hallucinations (all)** | 0.2 (0.15-0.25) | Small | -0.47 (-0.51--0.44) | Small | -0.61 (-0.64--0.58) | Medium | 0.31 (0.3-0.33) | Medium |
| **Hallucinations (auditory)** | 0.18 (0.14-0.23) | Negligible | -0.49 (-0.52--0.45) | Small | -0.59 (-0.62--0.56) | Medium | 0.31 (0.29-0.32) | Medium |
| **Hallucinations (OTG)** | 0.08 (0.03-0.12) | Negligible | -0.16 (-0.19--0.12) | Negligible | -0.2 (-0.26--0.18) | Small | 0.1 (0.09-0.12) | Small |
| **Hallucinations (visual)** | 0.13 (0.08-0.18) | Negligible | -0.21 (-0.25--0.17) | Small | -0.32 (-0.34--0.29) | Small | 0.16 (0.15-0.17) | Small |
| **Hostility** | 0.25 (0.2-0.31) | Small | -0.2 (-0.24--0.17) | Small | -0.4 (-0.42--0.37) | Small | 0.2 (0.16-0.21) | Small |
| **Insomnia** | 0.18 (0.13-0.23) | Negligible | 0.14 (0.09-0.18) | Negligible | -0.04 (-0.07--0.02) | Negligible | 0.06 (0.04-0.07) | Negligible |
| **Irritability** | 0.41 (0.36-0.46) | Small | -0.03 (-0.07-0.01) | Negligible | -0.37 (-0.39--0.34) | Small | 0.19 (0.15-0.2) | Small |
| **Loss of coherence** | 0.23 (0.18-0.28) | Small | -0.18 (-0.21--0.15) | Negligible | -0.3 (-0.33--0.27) | Small | 0.15 (0.14-0.17) | Small |
| **Low energy** | 0.15 (0.11-0.2) | Negligible | 0.21 (0.17-0.26) | Small | 0.07 (0.05-0.1) | Negligible | 0.07 (0.05-0.08) | Negligible |
| **MDMA use** | 0.07 (0.02-0.12) | Negligible | 0.02 (-0.02-0.07) | Negligible | -0.04 (-0.07--0.02) | Negligible | 0.09 (0.08-0.11) | Negligible |
| **Mood instability** | 0.51 (0.46-0.56) | Medium | 0.31 (0.26-0.36) | Small | -0.18 (-0.2--0.15) | Negligible | 0.17 (0.14-0.18) | Small |
| **Mutism** | 0.07 (0.03-0.12) | Negligible | -0.16 (-0.19--0.13) | Negligible | -0.2 (-0.22--0.17) | Small | 0.1 (0.09-0.11) | Small |
| **Negative symptom** | 0.1 (0.05-0.15) | Negligible | -0.31 (-0.34--0.28) | Small | -0.34 (-0.37--0.31) | Small | 0.18 (0.15-0.19) | Small |
| **Nightmare** | -0.07 (-0.11--0.03) | Negligible | -0.04 (-0.08-0) | Negligible | 0.02 (0-0.05) | Negligible | 0.02 (0.01-0.03) | Negligible |
| **Paranoia** | 0.31 (0.26-0.36) | Small | -0.5 (-0.54--0.46) | Medium | -0.72 (-0.74--0.69) | Medium | 0.37 (0.35-0.38) | Medium |
| **Passivity** | 0.07 (0.02-0.12) | Negligible | -0.23 (-0.26--0.2) | Small | -0.26 (-0.29--0.23) | Small | 0.14 (0.12-0.15) | Small |
| **Persecutory delusions** | 0.23 (0.18-0.28) | Small | -0.43 (-0.46--0.4) | Small | -0.56 (-0.59--0.53) | Medium | 0.29 (0.28-0.3) | Medium |
| **Poor appetite** | 0.12 (0.07-0.17) | Negligible | -0.02 (-0.07-0.02) | Negligible | -0.15 (-0.17--0.12) | Negligible | 0.07 (0.06-0.08) | Negligible |
| **Poor concentration** | 0.25 (0.2-0.29) | Small | 0.09 (0.05-0.14) | Negligible | -0.14 (-0.17--0.12) | Negligible | 0.09 (0.07-0.1) | Negligible |
| **Poor insight** | 0.34 (0.29-0.39) | Small | -0.03 (-0.07-0.01) | Negligible | -0.34 (-0.37--0.32) | Small | 0.17 (0.14-0.18) | Small |
| **Poor motivation** | 0.17 (0.12-0.22) | Negligible | 0.03 (-0.01-0.07) | Negligible | -0.13 (-0.15--0.1) | Negligible | 0.07 (0.06-0.08) | Negligible |
| **Poverty of speech** | 0.1 (0.05-0.15) | Negligible | -0.11 (-0.15--0.07) | Negligible | -0.19 (-0.22--0.16) | Negligible | 0.09 (0.08-0.11) | Negligible |
| **Poverty of thought** | 0.08 (0.03-0.13) | Negligible | -0.12 (-0.16--0.09) | Negligible | -0.16 (-0.19--0.14) | Negligible | 0.08 (0.07-0.1) | Negligible |
| **Social withdrawal** | 0.12 (0.07-0.17) | Negligible | -0.07 (-0.11--0.03) | Negligible | -0.18 (-0.2--0.15) | Negligible | 0.09 (0.07-0.1) | Negligible |
| **Stupor** | 0.05 (0-0.1) | Negligible | -0.04 (-0.07--0.01) | Negligible | -0.06 (-0.09--0.04) | Negligible | 0.03 (0.02-0.04) | Negligible |
| **Suicidality** | 0.06 (0.02-0.11) | Negligible | 0.07 (0.02-0.11) | Negligible | 0.01 (-0.02-0.03) | Negligible | 0.02 (0.01-0.03) | Negligible |
| **Tangential speech** | 0.27 (0.22-0.32) | Small | -0.16 (-0.19--0.12) | Negligible | -0.34 (-0.37--0.32) | Small | 0.17 (0.14-0.19) | Small |
| **Tearfulness** | 0.11 (0.06-0.15) | Negligible | 0.16 (0.12-0.21) | Negligible | 0.07 (0.04-0.09) | Negligible | 0.05 (0.04-0.06) | Negligible |
| **Thought block** | 0.12 (0.07-0.17) | Negligible | -0.18 (-0.22--0.15) | Negligible | -0.25 (-0.27--0.22) | Small | 0.13 (0.12-0.14) | Small |
| **Thought broadcast** | 0.13 (0.08-0.18) | Negligible | -0.22 (-0.25--0.18) | Small | -0.29 (-0.31--0.26) | Small | 0.15 (0.14-0.16) | Small |
| **Thought insertion** | 0.12 (0.07-0.17) | Negligible | -0.22 (-0.25--0.19) | Small | -0.28 (-0.31--0.26) | Small | 0.15 (0.14-0.16) | Small |
| **Thought withdrawal** | 0.05 (0-0.1) | Negligible | -0.15 (-0.18--0.12) | Negligible | -0.18 (-0.2--0.15) | Negligible | 0.09 (0.08-0.1) | Negligible |
| **Tobacco use** | 0.22 (0.17-0.27) | Small | -0.16 (-0.19--0.12) | Negligible | -0.33 (-0.35--0.3) | Small | 0.03 (0.01-0.04) | Negligible |
| **Waxy flexibility** | 0 (0-0) | Negligible | -0.04 (-0.07--0.01) | Negligible | -0.04 (-0.07--0.01) | Negligible | 0.16 (0.13-0.17) | Small |
| **Weight loss** | 0.11 (0.06-0.16) | Negligible | -0.07 (-0.11--0.02) | Negligible | -0.18 (-0.2--0.15) | Negligible | 0.02 (0.01-0.03) | Negligible |

# **eTable 18 Discriminability scores for feature normalised frequency. Pair-wise and three-wise discriminability scores were computed as in eTable 16. (N=26 975).**

|  | **Pair-wise discriminability scores** | | | | | | **Three-wise discriminability scores** | |
| --- | --- | --- | --- | --- | --- | --- | --- | --- |
|  | **BMD-UMD** | | **PSY-BMD** | | **PSY-UMD** | |  |  |
|  | **Cohen’s d effect size (95%CI)** | **Magnitude** | **Cohen’s d effect size (95%CI)** | **Magnitude** | **Cohen’s d effect size (95%CI)** | **Magnitude** | **Cohen’s f effect size (95%CI)** | **Magnitude** |
| **Aggression** | 0.07 (0.03-0.11) | Negligible | -0.11 (-0.15--0.08) | Negligible | -0.16 (-0.19--0.13) | Negligible | 0.08 (0.07-0.09) | Small |
| **Agitation** | 0.1 (0.06-0.14) | Negligible | -0.04 (-0.08-0) | Negligible | -0.13 (-0.16--0.11) | Negligible | 0.06 (0.05-0.08) | Negligible |
| **Anergia** | 0 (-0.04-0.04) | Negligible | 0.06 (0.01-0.1) | Negligible | 0.04 (0.02-0.07) | Negligible | 0.02 (0.01-0.03) | Negligible |
| **Anhedonia** | -0.06 (-0.09--0.03) | Negligible | 0.12 (0.07-0.17) | Negligible | 0.11 (0.09-0.14) | Negligible | 0.05 (0.04-0.06) | Negligible |
| **Anxiety** | -0.17 (-0.21--0.13) | Negligible | 0.18 (0.13-0.22) | Negligible | 0.32 (0.29-0.34) | Small | 0.15 (0.14-0.16) | Small |
| **Apathy** | -0.02 (-0.05-0.01) | Negligible | 0.01 (-0.03-0.06) | Negligible | 0.03 (0-0.05) | Negligible | 0.01 (0-0.02) | Negligible |
| **Arousal** | 0.03 (0-0.06) | Negligible | -0.02 (-0.06-0.02) | Negligible | -0.04 (-0.06--0.01) | Negligible | 0.02 (0-0.03) | Negligible |
| **Bad dreams** | -0.04 (-0.07-0) | Negligible | -0.02 (-0.06-0.02) | Negligible | 0.01 (-0.01-0.04) | Negligible | 0.01 (0-0.02) | Negligible |
| **Blunted affect** | -0.05 (-0.09--0.02) | Negligible | -0.08 (-0.12--0.04) | Negligible | -0.01 (-0.04-0.01) | Negligible | 0.02 (0-0.03) | Negligible |
| **Cannabis use** | 0 (-0.04-0.03) | Negligible | -0.18 (-0.21--0.15) | Negligible | -0.14 (-0.16--0.11) | Negligible | 0.07 (0.06-0.08) | Negligible |
| **Circumstantial speech** | 0.07 (0.03-0.12) | Negligible | -0.05 (-0.09--0.01) | Negligible | -0.11 (-0.13--0.08) | Negligible | 0.05 (0.04-0.06) | Negligible |
| **Cocaine use** | 0.02 (-0.02-0.07) | Negligible | 0 (-0.05-0.04) | Negligible | -0.03 (-0.06--0.01) | Negligible | 0.07 (0.06-0.08) | Negligible |
| **Cognitive impairment** | -0.18 (-0.21--0.14) | Negligible | -0.09 (-0.13--0.05) | Negligible | 0.09 (0.07-0.12) | Negligible | 0.06 (0.05-0.07) | Negligible |
| **Concrete thinking** | -0.01 (-0.05-0.03) | Negligible | -0.07 (-0.11--0.04) | Negligible | -0.06 (-0.08--0.03) | Negligible | 0.03 (0.02-0.04) | Negligible |
| **Delusions** | 0.15 (0.1-0.2) | Negligible | -0.25 (-0.33--0.22) | Small | -0.31 (-0.34--0.29) | Small | 0.16 (0.13-0.18) | Small |
| **Derailment of speech** | 0.06 (0.01-0.11) | Negligible | -0.04 (-0.07-0) | Negligible | -0.08 (-0.11--0.05) | Negligible | 0.04 (0.03-0.05) | Negligible |
| **Disturbed sleep** | 0 (-0.04-0.04) | Negligible | 0.14 (0.09-0.18) | Negligible | 0.12 (0.1-0.14) | Negligible | 0.06 (0.05-0.07) | Negligible |
| **Diurnal mood** | 0 (-0.04-0.04) | Negligible | 0.12 (0.06-0.17) | Negligible | 0.08 (0.06-0.11) | Negligible | 0.04 (0.03-0.05) | Negligible |
| **Early morning wakening** | 0.02 (-0.03-0.07) | Negligible | 0.09 (0.03-0.14) | Negligible | 0.09 (0.06-0.11) | Negligible | 0.04 (0.03-0.05) | Negligible |
| **Echolalia** | 0.03 (-0.02-0.08) | Negligible | -0.02 (-0.05-0.01) | Negligible | -0.03 (-0.05-0) | Negligible | 0.01 (0-0.03) | Negligible |
| **Elation** | 0.19 (0.14-0.25) | Negligible | 0.13 (0.08-0.18) | Negligible | -0.08 (-0.11--0.06) | Negligible | 0.08 (0.07-0.1) | Negligible |
| **Emotionally withdrawn** | -0.04 (-0.08-0) | Negligible | -0.09 (-0.12--0.05) | Negligible | -0.05 (-0.08--0.03) | Negligible | 0.03 (0.02-0.04) | Negligible |
| **Feeling helpless** | -0.08 (-0.12--0.05) | Negligible | 0.04 (0-0.09) | Negligible | 0.11 (0.09-0.14) | Negligible | 0.05 (0.04-0.07) | Negligible |
| **Feeling hopeless** | -0.12 (-0.15--0.08) | Negligible | 0.1 (0.06-0.15) | Negligible | 0.2 (0.17-0.22) | Small | 0.09 (0.08-0.1) | Negligible |
| **Feeling lonely** | -0.12 (-0.15--0.09) | Negligible | -0.03 (-0.06-0.01) | Negligible | 0.09 (0.06-0.13) | Negligible | 0.05 (0.03-0.06) | Negligible |
| **Feeling worthless** | -0.08 (-0.11--0.04) | Negligible | 0.12 (0.08-0.17) | Negligible | 0.15 (0.13-0.18) | Negligible | 0.01 (0-0.02) | Negligible |
| **Flight of ideas** | 0.11 (0.05-0.16) | Negligible | 0.07 (0.02-0.13) | Negligible | -0.11 (-0.13--0.08) | Negligible | 0.06 (0.05-0.07) | Negligible |
| **Formal thought disorder** | 0.03 (-0.02-0.08) | Negligible | -0.07 (-0.1--0.04) | Negligible | -0.08 (-0.11--0.06) | Negligible | 0.04 (0.03-0.05) | Negligible |
| **Grandiosity** | 0.12 (0.06-0.18) | Negligible | 0.05 (0-0.1) | Negligible | -0.21 (-0.27--0.18) | Small | 0.08 (0.07-0.09) | Negligible |
| **Guilt** | -0.12 (-0.15--0.08) | Negligible | 0.06 (0.03-0.09) | Negligible | 0.13 (0.11-0.16) | Negligible | 0.07 (0.05-0.08) | Negligible |
| **Hallucinations (all)** | 0.06 (0.01-0.11) | Negligible | -0.24 (-0.29--0.2) | Small | -0.34 (-0.37--0.32) | Small | 0.17 (0.14-0.18) | Small |
| **Hallucinations (auditory)** | 0.05 (0-0.1) | Negligible | -0.24 (-0.28--0.2) | Small | -0.31 (-0.34--0.29) | Small | 0.16 (0.14-0.17) | Small |
| **Hallucinations (OTG)** | 0.03 (-0.02-0.08) | Negligible | -0.03 (-0.08-0.02) | Negligible | -0.11 (-0.13--0.08) | Negligible | 0.04 (0.03-0.05) | Negligible |
| **Hallucinations (visual)** | 0.04 (-0.01-0.08) | Negligible | -0.05 (-0.08--0.02) | Negligible | -0.07 (-0.1--0.04) | Negligible | 0.04 (0.02-0.05) | Negligible |
| **Hostility** | 0.02 (-0.01-0.04) | Negligible | -0.08 (-0.11--0.05) | Negligible | -0.07 (-0.1--0.05) | Negligible | 0.04 (0.02-0.05) | Negligible |
| **Insomnia** | -0.02 (-0.06-0.01) | Negligible | 0.12 (0.07-0.16) | Negligible | 0.11 (0.09-0.14) | Negligible | 0.05 (0.04-0.06) | Negligible |
| **Irritability** | 0.11 (0.07-0.16) | Negligible | 0.06 (0.02-0.11) | Negligible | -0.06 (-0.08--0.03) | Negligible | 0.04 (0.03-0.05) | Negligible |
| **Loss of coherence** | 0.08 (0.04-0.13) | Negligible | -0.1 (-0.13--0.07) | Negligible | -0.13 (-0.16--0.11) | Negligible | 0.07 (0.06-0.08) | Negligible |
| **Low energy** | -0.05 (-0.09--0.02) | Negligible | 0.2 (0.15-0.25) | Small | 0.17 (0.15-0.2) | Negligible | 0.08 (0.07-0.09) | Negligible |
| **MDMA use** | 0.01 (-0.03-0.04) | Negligible | 0.02 (-0.02-0.06) | Negligible | 0.01 (-0.02-0.03) | Negligible | 0.01 (0-0.03) | Negligible |
| **Mood instability** | 0.2 (0.15-0.25) | Small | 0.27 (0.22-0.33) | Small | 0.11 (0.09-0.14) | Negligible | 0.11 (0.1-0.13) | Small |
| **Mutism** | -0.03 (-0.06-0) | Negligible | -0.11 (-0.14--0.08) | Negligible | -0.08 (-0.1--0.05) | Negligible | 0.04 (0.03-0.05) | Negligible |
| **Negative symptom** | 0.01 (-0.02-0.05) | Negligible | -0.18 (-0.21--0.15) | Negligible | -0.18 (-0.21--0.15) | Negligible | 0.1 (0.08-0.11) | Small |
| **Nightmare** | -0.13 (-0.17--0.1) | Negligible | -0.02 (-0.06-0.02) | Negligible | 0.12 (0.09-0.14) | Negligible | 0.06 (0.05-0.07) | Negligible |
| **Paranoia** | 0.06 (0.03-0.1) | Negligible | -0.43 (-0.47--0.4) | Small | -0.44 (-0.47--0.41) | Small | 0.23 (0.22-0.24) | Small |
| **Passivity** | 0.05 (0-0.11) | Negligible | -0.1 (-0.13--0.06) | Negligible | -0.14 (-0.17--0.12) | Negligible | 0.07 (0.06-0.09) | Negligible |
| **Persecutory delusions** | 0.05 (0-0.09) | Negligible | -0.24 (-0.32--0.21) | Small | -0.26 (-0.29--0.24) | Small | 0.14 (0.13-0.15) | Small |
| **Poor appetite** | -0.05 (-0.08--0.01) | Negligible | 0 (-0.04-0.04) | Negligible | 0.04 (0.01-0.06) | Negligible | 0.02 (0.01-0.03) | Negligible |
| **Poor concentration** | -0.05 (-0.08--0.02) | Negligible | 0.13 (0.08-0.17) | Negligible | 0.13 (0.11-0.16) | Negligible | 0.06 (0.05-0.07) | Negligible |
| **Poor insight** | 0.06 (0.02-0.09) | Negligible | 0.04 (0-0.09) | Negligible | -0.02 (-0.04-0.01) | Negligible | 0.02 (0-0.03) | Negligible |
| **Poor motivation** | -0.02 (-0.06-0.01) | Negligible | 0.07 (0.02-0.11) | Negligible | 0.07 (0.05-0.1) | Negligible | 0.04 (0.02-0.05) | Negligible |
| **Poverty of speech** | 0 (-0.04-0.04) | Negligible | -0.08 (-0.11--0.04) | Negligible | -0.07 (-0.1--0.05) | Negligible | 0.04 (0.03-0.05) | Negligible |
| **Poverty of thought** | -0.02 (-0.04-0.01) | Negligible | -0.08 (-0.11--0.06) | Negligible | -0.06 (-0.08--0.03) | Negligible | 0.03 (0.02-0.04) | Negligible |
| **Social withdrawal** | -0.01 (-0.05-0.02) | Negligible | -0.02 (-0.06-0.02) | Negligible | -0.01 (-0.03-0.02) | Negligible | 0.01 (0-0.02) | Negligible |
| **Stupor** | 0.03 (-0.02-0.08) | Negligible | -0.03 (-0.05-0) | Negligible | -0.03 (-0.06--0.01) | Negligible | 0.02 (0-0.03) | Negligible |
| **Suicidality** | -0.1 (-0.13--0.07) | Negligible | 0.07 (0.03-0.11) | Negligible | 0.13 (0.1-0.15) | Negligible | 0.06 (0.05-0.07) | Negligible |
| **Tangential speech** | 0.14 (0.09-0.19) | Negligible | -0.04 (-0.08-0) | Negligible | -0.17 (-0.2--0.15) | Negligible | 0.08 (0.07-0.1) | Negligible |
| **Tearfulness** | -0.18 (-0.22--0.15) | Negligible | 0.17 (0.13-0.22) | Small | 0.3 (0.28-0.33) | Small | 0.14 (0.13-0.16) | Small |
| **Thought block** | 0.03 (-0.01-0.08) | Negligible | -0.06 (-0.09--0.03) | Negligible | -0.07 (-0.1--0.05) | Negligible | 0.04 (0.03-0.05) | Negligible |
| **Thought broadcast** | 0.05 (0-0.1) | Negligible | -0.1 (-0.13--0.06) | Negligible | -0.13 (-0.15--0.1) | Negligible | 0.07 (0.05-0.08) | Negligible |
| **Thought insertion** | 0.02 (-0.02-0.06) | Negligible | -0.14 (-0.17--0.12) | Negligible | -0.15 (-0.17--0.12) | Negligible | 0.08 (0.07-0.09) | Negligible |
| **Thought withdrawal** | 0 (-0.04-0.03) | Negligible | -0.1 (-0.13--0.08) | Negligible | -0.1 (-0.12--0.07) | Negligible | 0.05 (0.04-0.06) | Negligible |
| **Tobacco use** | -0.01 (-0.04-0.02) | Negligible | -0.11 (-0.14--0.08) | Negligible | -0.08 (-0.11--0.06) | Negligible | 0.01 (0-0.02) | Negligible |
| **Waxy flexibility** | 0 (0-0) | Negligible | -0.03 (-0.06--0.01) | Negligible | -0.03 (-0.06--0.01) | Negligible | 0.04 (0.03-0.06) | Negligible |
| **Weight loss** | -0.04 (-0.08-0.01) | Negligible | -0.02 (-0.06-0.02) | Negligible | 0.01 (-0.01-0.04) | Negligible | 0.02 (0-0.03) | Negligible |

# **eResults 2 Discriminability analysis for feature normalised frequency**

Compared to the occurrences, normalised frequency showed overall reduced three-wise and pair-wise discriminability scores.

Specifically, for the three-wise discriminability scores, out of the 30 features with small effect sizes calculated with feature occurrences, all features were reduced to negligible effect sizes with the exception of 4 features (remained small), and the 5 features with medium effects sizes were reduced to small effect sizes in the normalised frequency analysis.

For the two-wise discriminability scores, all the small effect sizes for the comparison BMD-UMD calculated with feature occurrences were reduced to negligible effect sizes in the normalised frequency analysis, and the only feature with a medium effect size (mood instability) was reduced to a small effect size. Similarly, for the comparison PSY-BMD, out of the 13 features with small effect sizes, all the effect sizes were reduced to negligible effect sizes in the normalised frequency analysis, except for 6 features (remained small), and the only feature with a medium effect size (passivity) was reduced to a small effect size. For the comparison PSY-UMD, out of the 28 features with small effect sizes, all the effect sizes were reduced to negligible effect sizes in the normalised frequency analysis, except for 2 features (remained small), and the 4 features with medium effects sizes were reduced to small effect sizes in the normalised frequency analysis.

These results confirm that the occurrences metric used in the main analysis is more robust to discriminate between SMD groups, and could suggest that the presence/absence of a feature is more informative than the number of times it is recorded in a clinical record.

**eTable 19 Discriminability scores for feature occurrences in sensitivity analyses.**

|  | **Pair-wise discriminability scores** | | | | | | | | **Three-wise discriminability scores** | |  |
| --- | --- | --- | --- | --- | --- | --- | --- | --- | --- | --- | --- |
|  | | **≤35 years of age (N=10 491)** | | | | | | | | | |
|  | | **BMD-UMD** | | **BMD-PSY** | | **UMD-PSY** | |  | |  | |
|  | | **Cohen’s d effect size (95%CI)** | **Magnitude** | **Cohen’s d effect size (95%CI)** | **Magnitude** | **Cohen’s d effect size (95%CI)** | **Magnitude** | **Cohen’s f effect size (95%CI)** | | **Magnitude** | |
| **Aggression** | | 0.23 (0.15-0.32) | Small | -0.24 (-0.3--0.18) | Small | -0.45 (-0.49--0.4) | Small | 0.22 (0.21-0.24) | | Small | |
| **Agitation** | | 0.38 (0.29-0.47) | Small | -0.19 (-0.25--0.12) | Negligible | -0.55 (-0.59--0.5) | Medium | 0.27 (0.22-0.29) | | Medium | |
| **Anergia** | | 0.1 (0.02-0.18) | Negligible | 0.07 (0-0.15) | Negligible | -0.02 (-0.06-0.02) | Negligible | 0.03 (0-0.05) | | Negligible | |
| **Anhedonia** | | 0.12 (0.05-0.2) | Negligible | 0.08 (0-0.15) | Negligible | -0.04 (-0.08-0) | Negligible | 0.04 (0.02-0.06) | | Negligible | |
| **Anxiety** | | 0.24 (0.16-0.32) | Small | -0.01 (-0.08-0.06) | Negligible | -0.25 (-0.29--0.21) | Small | 0.12 (0.1-0.14) | | Small | |
| **Apathy** | | 0.15 (0.07-0.23) | Negligible | -0.09 (-0.16--0.03) | Negligible | -0.22 (-0.26--0.18) | Small | 0.11 (0.09-0.13) | | Small | |
| **Arousal** | | 0.21 (0.13-0.3) | Small | -0.2 (-0.26--0.13) | Small | -0.38 (-0.42--0.34) | Small | 0.19 (0.17-0.21) | | Small | |
| **Bad dreams** | | 0.01 (-0.07-0.09) | Negligible | -0.03 (-0.11-0.05) | Negligible | -0.06 (-0.1--0.01) | Negligible | 0.03 (0-0.04) | | Negligible | |
| **Blunted affect** | | 0.12 (0.04-0.2) | Negligible | -0.16 (-0.23--0.09) | Negligible | -0.29 (-0.33--0.25) | Small | 0.14 (0.12-0.16) | | Small | |
| **Cannabis use** | | 0.27 (0.19-0.35) | Small | -0.3 (-0.37--0.24) | Small | -0.51 (-0.55--0.47) | Medium | 0.26 (0.21-0.28) | | Medium | |
| **Circumstantial speech** | | 0.25 (0.16-0.34) | Small | -0.18 (-0.23--0.12) | Negligible | -0.34 (-0.38--0.29) | Small | 0.18 (0.16-0.19) | | Small | |
| **Cocaine use** | | 0.25 (0.17-0.33) | Small | -0.1 (-0.16--0.04) | Negligible | -0.27 (-0.31--0.23) | Small | 0.14 (0.12-0.16) | | Small | |
| **Cognitive impairment** | | 0.23 (0.15-0.31) | Small | -0.15 (-0.21--0.08) | Negligible | -0.37 (-0.41--0.33) | Small | 0.18 (0.16-0.2) | | Small | |
| **Concrete thinking** | | 0.01 (-0.06-0.07) | Negligible | -0.15 (-0.2--0.1) | Negligible | -0.15 (-0.2--0.11) | Negligible | 0.08 (0.06-0.1) | | Negligible | |
| **Delusions** | | 0.44 (0.35-0.53) | Small | -0.47 (-0.61--0.42) | Small | -0.74 (-0.78--0.69) | Medium | 0.39 (0.37-0.41) | | Medium | |
| **Derailment of speech** | | 0.14 (0.05-0.23) | Negligible | -0.16 (-0.21--0.1) | Negligible | -0.25 (-0.29--0.21) | Small | 0.13 (0.11-0.15) | | Small | |
| **Disturbed sleep** | | 0.32 (0.24-0.4) | Small | 0 (-0.07-0.07) | Negligible | -0.31 (-0.41--0.27) | Small | 0.16 (0.14-0.18) | | Small | |
| **Diurnal mood** | | 0.07 (-0.01-0.15) | Negligible | 0.1 (0.02-0.19) | Negligible | 0.03 (-0.01-0.07) | Negligible | 0.03 (0.01-0.05) | | Negligible | |
| **Early morning wakening** | | 0.13 (0.05-0.21) | Negligible | 0.13 (0.05-0.22) | Negligible | 0 (-0.04-0.04) | Negligible | 0.05 (0.03-0.06) | | Negligible | |
| **Echolalia** | | 0.06 (-0.02-0.15) | Negligible | -0.06 (-0.12-0) | Negligible | -0.11 (-0.15--0.06) | Negligible | 0.05 (0.03-0.07) | | Negligible | |
| **Elation** | | 0.68 (0.58-0.78) | Medium | 0.15 (0.08-0.22) | Negligible | -0.37 (-0.48--0.33) | Small | 0.22 (0.2-0.24) | | Small | |
| **Emotionally withdrawn** | | 0.14 (0.05-0.22) | Negligible | -0.2 (-0.27--0.14) | Small | -0.35 (-0.39--0.3) | Small | 0.17 (0.15-0.19) | | Small | |
| **Feeling helpless** | | 0.05 (-0.02-0.13) | Negligible | 0.06 (-0.01-0.14) | Negligible | 0.01 (-0.03-0.05) | Negligible | 0.02 (0-0.03) | | Negligible | |
| **Feeling hopeless** | | 0.18 (0.1-0.26) | Negligible | 0.15 (0.07-0.23) | Negligible | -0.03 (-0.07-0.01) | Negligible | 0.05 (0.03-0.07) | | Negligible | |
| **Feeling lonely** | | 0.03 (-0.04-0.11) | Negligible | -0.08 (-0.15--0.01) | Negligible | -0.12 (-0.16--0.08) | Negligible | 0.06 (0.04-0.08) | | Negligible | |
| **Feeling worthless** | | 0.19 (0.11-0.27) | Negligible | 0.21 (0.13-0.29) | Small | 0.04 (-0.01-0.08) | Negligible | 0.07 (0.05-0.09) | | Negligible | |
| **Flight of ideas** | | 0.43 (0.33-0.52) | Small | 0.05 (-0.02-0.12) | Negligible | -0.31 (-0.36--0.27) | Small | 0.17 (0.15-0.19) | | Small | |
| **Formal thought disorder** | | 0.14 (0.04-0.23) | Negligible | -0.16 (-0.23--0.1) | Negligible | -0.29 (-0.33--0.24) | Small | 0.15 (0.13-0.18) | | Small | |
| **Grandiosity** | | 0.5 (0.41-0.6) | Small | -0.07 (-0.13--0.01) | Negligible | -0.4 (-0.45--0.36) | Small | 0.21 (0.19-0.23) | | Small | |
| **Guilt** | | 0.14 (0.06-0.23) | Negligible | 0.11 (0.03-0.19) | Negligible | -0.04 (-0.08-0) | Negligible | 0.05 (0.03-0.07) | | Negligible | |
| **Hallucinations (all)** | | 0.33 (0.24-0.41) | Small | -0.51 (-0.65--0.45) | Medium | -0.73 (-0.78--0.68) | Medium | 0.38 (0.36-0.4) | | Medium | |
| **Hallucinations (auditory)** | | 0.27 (0.19-0.36) | Small | -0.54 (-0.6--0.49) | Medium | -0.7 (-0.75--0.66) | Medium | 0.37 (0.35-0.39) | | Medium | |
| **Hallucinations (OTG)** | | 0.11 (0.02-0.19) | Negligible | -0.14 (-0.2--0.09) | Negligible | -0.21 (-0.26--0.17) | Small | 0.11 (0.09-0.13) | | Small | |
| **Hallucinations (visual)** | | 0.2 (0.11-0.28) | Small | -0.24 (-0.3--0.17) | Small | -0.4 (-0.45--0.36) | Small | 0.21 (0.19-0.22) | | Small | |
| **Hostility** | | 0.25 (0.16-0.34) | Small | -0.23 (-0.29--0.16) | Small | -0.44 (-0.49--0.4) | Small | 0.23 (0.21-0.24) | | Small | |
| **Insomnia** | | 0.27 (0.19-0.36) | Small | 0.12 (0.04-0.19) | Negligible | -0.16 (-0.2--0.12) | Negligible | 0.1 (0.08-0.12) | | Small | |
| **Irritability** | | 0.45 (0.36-0.54) | Small | -0.08 (-0.15--0.02) | Negligible | -0.43 (-0.47--0.38) | Small | 0.22 (0.2-0.24) | | Small | |
| **Loss of coherence** | | 0.24 (0.15-0.33) | Small | -0.24 (-0.3--0.19) | Small | -0.38 (-0.43--0.34) | Small | 0.2 (0.18-0.22) | | Small | |
| **Low energy** | | 0.19 (0.11-0.27) | Negligible | 0.2 (0.12-0.27) | Small | 0.01 (-0.03-0.05) | Negligible | 0.06 (0.04-0.08) | | Negligible | |
| **MDMA use** | | 0.13 (0.05-0.22) | Negligible | 0.05 (-0.03-0.13) | Negligible | -0.08 (-0.12--0.04) | Negligible | 0.05 (0.03-0.07) | | Negligible | |
| **Mood instability** | | 0.59 (0.5-0.68) | Medium | 0.36 (0.28-0.44) | Small | -0.22 (-0.26--0.18) | Small | 0.2 (0.18-0.22) | | Small | |
| **Mutism** | | 0.11 (0.03-0.19) | Negligible | -0.23 (-0.28--0.18) | Small | -0.28 (-0.32--0.23) | Small | 0.15 (0.13-0.17) | | Small | |
| **Negative symptom** | | 0.12 (0.04-0.2) | Negligible | -0.36 (-0.41--0.31) | Small | -0.4 (-0.44--0.35) | Small | 0.22 (0.2-0.24) | | Small | |
| **Nightmare** | | -0.03 (-0.09-0.04) | Negligible | -0.04 (-0.11-0.03) | Negligible | -0.01 (-0.05-0.03) | Negligible | 0.01 (0-0.03) | | Negligible | |
| **Paranoia** | | 0.38 (0.3-0.47) | Small | -0.5 (-0.65--0.44) | Medium | -0.78 (-0.82--0.73) | Medium | 0.4 (0.38-0.42) | | Medium | |
| **Passivity** | | 0.13 (0.04-0.22) | Negligible | -0.26 (-0.32--0.21) | Small | -0.33 (-0.37--0.28) | Small | 0.18 (0.16-0.2) | | Small | |
| **Persecutory delusions** | | 0.27 (0.19-0.36) | Small | -0.45 (-0.51--0.4) | Small | -0.62 (-0.66--0.57) | Medium | 0.33 (0.26-0.35) | | Small | |
| **Poor appetite** | | 0.13 (0.05-0.21) | Negligible | -0.13 (-0.19--0.06) | Negligible | -0.24 (-0.28--0.19) | Small | 0.12 (0.1-0.14) | | Small | |
| **Poor concentration** | | 0.3 (0.22-0.38) | Small | 0 (-0.07-0.08) | Negligible | -0.29 (-0.37--0.25) | Small | 0.15 (0.13-0.17) | | Small | |
| **Poor insight** | | 0.4 (0.32-0.49) | Small | -0.09 (-0.16--0.02) | Negligible | -0.46 (-0.51--0.42) | Small | 0.23 (0.21-0.25) | | Small | |
| **Poor motivation** | | 0.15 (0.08-0.23) | Negligible | -0.07 (-0.14-0) | Negligible | -0.22 (-0.26--0.18) | Small | 0.11 (0.09-0.13) | | Small | |
| **Poverty of speech** | | 0.08 (0-0.16) | Negligible | -0.17 (-0.23--0.11) | Negligible | -0.23 (-0.27--0.19) | Small | 0.12 (0.1-0.14) | | Small | |
| **Poverty of thought** | | 0.07 (-0.01-0.16) | Negligible | -0.16 (-0.21--0.11) | Negligible | -0.19 (-0.23--0.14) | negligible | 0.1 (0.08-0.12) | | Small | |
| **Social withdrawal** | | 0.16 (0.07-0.24) | Negligible | -0.14 (-0.21--0.08) | Negligible | -0.28 (-0.32--0.23) | Small | 0.14 (0.12-0.16) | | Small | |
| **Stupor** | | 0.07 (-0.02-0.16) | Negligible | -0.02 (-0.09-0.04) | Negligible | -0.07 (-0.12--0.03) | Negligible | 0.04 (0.02-0.06) | | Negligible | |
| **Suicidality** | | 0.12 (0.05-0.2) | Negligible | 0.09 (0.01-0.16) | Negligible | -0.03 (-0.07-0.01) | Negligible | 0.04 (0.01-0.05) | | Negligible | |
| **Tangential speech** | | 0.28 (0.19-0.37) | Small | -0.23 (-0.29--0.17) | Small | -0.44 (-0.48--0.39) | Small | 0.23 (0.21-0.25) | | Small | |
| **Tearfulness** | | 0.17 (0.1-0.25) | Negligible | 0.17 (0.1-0.25) | Negligible | 0.01 (-0.03-0.05) | Negligible | 0.05 (0.03-0.07) | | Negligible | |
| **Thought block** | | 0.21 (0.12-0.29) | Small | -0.22 (-0.28--0.17) | Small | -0.33 (-0.37--0.29) | Small | 0.17 (0.15-0.19) | | Small | |
| **Thought broadcast** | | 0.16 (0.07-0.24) | Negligible | -0.27 (-0.32--0.22) | Small | -0.35 (-0.45--0.3) | Small | 0.18 (0.17-0.2) | | Small | |
| **Thought insertion** | | 0.15 (0.06-0.23) | Negligible | -0.26 (-0.32--0.21) | Small | -0.35 (-0.39--0.3) | Small | 0.18 (0.16-0.2) | | Small | |
| **Thought withdrawal** | | 0.08 (-0.01-0.16) | Negligible | -0.19 (-0.24--0.13) | Negligible | -0.24 (-0.28--0.19) | Small | 0.12 (0.1-0.14) | | Small | |
| **Tobacco use** | | 0.2 (0.12-0.28) | Small | -0.27 (-0.33--0.21) | Small | -0.4 (-0.44--0.36) | Small | 0.21 (0.19-0.23) | | Small | |
| **Waxy flexibility** | | 0 (0-0) | Negligible | -0.06 (-0.11--0.02) | Negligible | -0.06 (-0.11--0.02) | Negligible | 0.04 (0.01-0.05) | | Negligible | |
| **Weight loss** | | 0.14 (0.06-0.22) | Negligible | -0.05 (-0.12-0.03) | Negligible | -0.21 (-0.25--0.17) | Small | 0.1 (0.08-0.12) | | Small | |
|  | | **Relevant medication at index excluded (N=13 021)** | | | | | | | | | |
| **Aggression** | | 0.14 (0.07-0.22) | Negligible | -0.14 (-0.2--0.08) | Negligible | -0.27 (-0.31--0.23) | Small | 0.14 (0.12-0.15) | | Small | |
| **Agitation** | | 0.27 (0.2-0.35) | Small | -0.09 (-0.16--0.03) | Negligible | -0.35 (-0.45--0.31) | Small | 0.18 (0.16-0.2) | | Small | |
| **Anergia** | | 0.11 (0.04-0.18) | Negligible | 0.15 (0.07-0.23) | Negligible | 0.04 (0-0.07) | Negligible | 0.04 (0.02-0.06) | | Negligible | |
| **Anhedonia** | | 0.08 (0.01-0.14) | Negligible | 0.14 (0.07-0.21) | Negligible | 0.06 (0.03-0.1) | Negligible | 0.04 (0.02-0.06) | | Negligible | |
| **Anxiety** | | 0.1 (0.03-0.17) | Negligible | 0.04 (-0.03-0.11) | Negligible | -0.06 (-0.1--0.02) | Negligible | 0.04 (0.02-0.05) | | Negligible | |
| **Apathy** | | 0.04 (-0.03-0.11) | Negligible | -0.08 (-0.14--0.02) | Negligible | -0.11 (-0.15--0.07) | Negligible | 0.05 (0.04-0.07) | | Negligible | |
| **Arousal** | | 0.14 (0.06-0.22) | Negligible | -0.09 (-0.16--0.03) | Negligible | -0.24 (-0.28--0.2) | Small | 0.12 (0.1-0.14) | | Small | |
| **Bad dreams** | | -0.06 (-0.12--0.01) | Negligible | -0.08 (-0.14--0.02) | Negligible | -0.01 (-0.05-0.02) | Negligible | 0.02 (0-0.03) | | Negligible | |
| **Blunted affect** | | -0.03 (-0.09-0.03) | Negligible | -0.17 (-0.22--0.11) | Negligible | -0.14 (-0.18--0.1) | Negligible | 0.07 (0.06-0.09) | | Negligible | |
| **Cannabis use** | | 0.15 (0.08-0.22) | Negligible | -0.16 (-0.22--0.1) | Negligible | -0.27 (-0.31--0.23) | Small | 0.14 (0.12-0.16) | | Small | |
| **Circumstantial speech** | | 0.15 (0.07-0.23) | Negligible | -0.07 (-0.13--0.01) | Negligible | -0.19 (-0.23--0.14) | Negligible | 0.1 (0.08-0.12) | | Small | |
| **Cocaine use** | | 0.11 (0.04-0.18) | Negligible | -0.04 (-0.1-0.02) | Negligible | -0.13 (-0.17--0.09) | Negligible | 0.07 (0.05-0.08) | | Negligible | |
| **Cognitive impairment** | | 0.05 (-0.02-0.12) | Negligible | -0.12 (-0.19--0.06) | Negligible | -0.17 (-0.21--0.13) | Negligible | 0.08 (0.06-0.1) | | Negligible | |
| **Concrete thinking** | | -0.05 (-0.1-0) | Negligible | -0.13 (-0.18--0.08) | Negligible | -0.11 (-0.15--0.06) | Negligible | 0.06 (0.04-0.08) | | Negligible | |
| **Delusions** | | 0.29 (0.22-0.37) | Small | -0.46 (-0.51--0.4) | Small | -0.58 (-0.63--0.54) | Medium | 0.33 (0.31-0.35) | | Medium | |
| **Derailment of speech** | | 0.08 (0-0.15) | Negligible | -0.11 (-0.16--0.05) | Negligible | -0.16 (-0.2--0.11) | Negligible | 0.09 (0.07-0.1) | | Negligible | |
| **Disturbed sleep** | | 0.18 (0.11-0.25) | Negligible | 0.11 (0.04-0.18) | Negligible | -0.07 (-0.11--0.03) | Negligible | 0.06 (0.04-0.07) | | Negligible | |
| **Diurnal mood** | | 0.05 (-0.02-0.12) | Negligible | 0.14 (0.06-0.22) | Negligible | 0.1 (0.06-0.13) | Negligible | 0.05 (0.03-0.06) | | Negligible | |
| **Early morning wakening** | | -0.01 (-0.07-0.05) | Negligible | 0.08 (0.01-0.15) | Negligible | 0.08 (0.04-0.12) | Negligible | 0.04 (0.02-0.05) | | Negligible | |
| **Echolalia** | | 0.02 (-0.05-0.09) | Negligible | -0.02 (-0.07-0.03) | Negligible | -0.03 (-0.07-0.01) | Negligible | 0.02 (0-0.03) | | Negligible | |
| **Elation** | | 0.56 (0.47-0.65) | Medium | 0.33 (0.25-0.41) | Small | -0.26 (-0.31--0.22) | Small | 0.24 (0.19-0.25) | | Small | |
| **Emotionally withdrawn** | | 0.04 (-0.03-0.11) | Negligible | -0.12 (-0.18--0.06) | Negligible | -0.16 (-0.2--0.12) | Negligible | 0.08 (0.06-0.09) | | Negligible | |
| **Feeling helpless** | | 0.02 (-0.05-0.09) | Negligible | 0.09 (0.02-0.16) | Negligible | 0.07 (0.04-0.11) | Negligible | 0.03 (0.02-0.05) | | Negligible | |
| **Feeling hopeless** | | 0.03 (-0.04-0.1) | Negligible | 0.14 (0.06-0.21) | Negligible | 0.12 (0.08-0.16) | Negligible | 0.05 (0.04-0.07) | | Negligible | |
| **Feeling lonely** | | -0.05 (-0.12-0.01) | Negligible | -0.02 (-0.09-0.05) | Negligible | 0.03 (-0.01-0.07) | Negligible | 0.02 (0-0.03) | | Negligible | |
| **Feeling worthless** | | 0.01 (-0.05-0.08) | Negligible | 0.12 (0.04-0.19) | Negligible | 0.1 (0.07-0.14) | Negligible | 0.05 (0.03-0.06) | | Negligible | |
| **Flight of ideas** | | 0.35 (0.26-0.43) | Small | 0.17 (0.1-0.25) | Negligible | -0.19 (-0.23--0.14) | Negligible | 0.14 (0.12-0.16) | | Small | |
| **Formal thought disorder** | | 0.07 (-0.01-0.15) | Negligible | -0.14 (-0.19--0.09) | Negligible | -0.18 (-0.22--0.14) | Negligible | 0.1 (0.08-0.12) | | Small | |
| **Grandiosity** | | 0.4 (0.31-0.49) | Small | 0.04 (-0.02-0.11) | Negligible | -0.25 (-0.3--0.21) | Small | 0.15 (0.14-0.17) | | Small | |
| **Guilt** | | 0.03 (-0.04-0.1) | Negligible | 0.16 (0.08-0.23) | Negligible | 0.13 (0.1-0.17) | Negligible | 0.06 (0.04-0.08) | | Negligible | |
| **Hallucinations (all)** | | 0.14 (0.07-0.21) | Negligible | -0.46 (-0.52--0.41) | Small | -0.54 (-0.59--0.5) | Medium | 0.3 (0.24-0.32) | | Medium | |
| **Hallucinations (auditory)** | | 0.13 (0.06-0.2) | Negligible | -0.46 (-0.51--0.41) | Small | -0.53 (-0.58--0.49) | Medium | 0.3 (0.24-0.32) | | Medium | |
| **Hallucinations (OTG)** | | 0.07 (-0.01-0.15) | Negligible | -0.13 (-0.19--0.07) | Negligible | -0.19 (-0.23--0.15) | Negligible | 0.1 (0.08-0.12) | | Small | |
| **Hallucinations (visual)** | | 0.09 (0.01-0.16) | Negligible | -0.18 (-0.24--0.12) | Negligible | -0.25 (-0.3--0.21) | Small | 0.13 (0.11-0.16) | | Small | |
| **Hostility** | | 0.16 (0.08-0.24) | Negligible | -0.17 (-0.23--0.1) | Negligible | -0.31 (-0.35--0.26) | Small | 0.16 (0.14-0.18) | | Small | |
| **Insomnia** | | 0.16 (0.09-0.24) | Negligible | 0.18 (0.11-0.26) | Negligible | 0.03 (-0.01-0.07) | Negligible | 0.06 (0.04-0.07) | | Negligible | |
| **Irritability** | | 0.27 (0.19-0.35) | Small | 0.01 (-0.06-0.07) | Negligible | -0.24 (-0.28--0.2) | Small | 0.13 (0.11-0.15) | | Small | |
| **Loss of coherence** | | 0.17 (0.09-0.25) | Negligible | -0.13 (-0.19--0.07) | Negligible | -0.25 (-0.29--0.2) | Small | 0.13 (0.12-0.16) | | Small | |
| **Low energy** | | 0.08 (0.01-0.15) | Negligible | 0.21 (0.14-0.29) | Small | 0.14 (0.11-0.18) | Negligible | 0.07 (0.05-0.09) | | Negligible | |
| **MDMA use** | | 0.05 (-0.02-0.11) | Negligible | 0.01 (-0.05-0.08) | Negligible | -0.03 (-0.07-0.01) | Negligible | 0.02 (0-0.03) | | Negligible | |
| **Mood instability** | | 0.42 (0.34-0.5) | Small | 0.41 (0.33-0.49) | Small | 0 (-0.04-0.03) | Negligible | 0.15 (0.13-0.17) | | Small | |
| **Mutism** | | -0.02 (-0.08-0.04) | Negligible | -0.18 (-0.22--0.13) | Negligible | -0.16 (-0.21--0.12) | Negligible | 0.09 (0.07-0.11) | | Negligible | |
| **Negative symptom** | | 0.08 (-0.01-0.16) | Negligible | -0.3 (-0.35--0.25) | Small | -0.32 (-0.37--0.28) | Small | 0.19 (0.17-0.21) | | Small | |
| **Nightmare** | | -0.1 (-0.15--0.04) | Negligible | -0.01 (-0.07-0.06) | Negligible | 0.08 (0.05-0.12) | Negligible | 0.04 (0.02-0.06) | | Negligible | |
| **Paranoia** | | 0.19 (0.11-0.26) | Negligible | -0.51 (-0.56--0.45) | Medium | -0.63 (-0.67--0.58) | Medium | 0.34 (0.33-0.36) | | Medium | |
| **Passivity** | | 0.07 (-0.01-0.15) | Negligible | -0.17 (-0.27--0.11) | Negligible | -0.22 (-0.26--0.17) | Small | 0.12 (0.1-0.14) | | Small | |
| **Persecutory delusions** | | 0.13 (0.05-0.2) | Negligible | -0.39 (-0.44--0.34) | Small | -0.45 (-0.49--0.4) | Small | 0.25 (0.2-0.27) | | Medium | |
| **Poor appetite** | | 0.08 (0-0.16) | Negligible | 0 (-0.07-0.07) | Negligible | -0.08 (-0.12--0.04) | Negligible | 0.04 (0.02-0.06) | | Negligible | |
| **Poor concentration** | | 0.12 (0.05-0.19) | Negligible | 0.12 (0.05-0.19) | Negligible | 0 (-0.04-0.04) | Negligible | 0.04 (0.02-0.05) | | Negligible | |
| **Poor insight** | | 0.25 (0.17-0.33) | Small | 0 (-0.07-0.06) | Negligible | -0.25 (-0.29--0.2) | Small | 0.13 (0.11-0.14) | | Small | |
| **Poor motivation** | | 0.09 (0.02-0.16) | Negligible | 0.03 (-0.04-0.1) | Negligible | -0.06 (-0.1--0.02) | Negligible | 0.03 (0.01-0.05) | | Negligible | |
| **Poverty of speech** | | 0.02 (-0.05-0.08) | Negligible | -0.14 (-0.19--0.08) | Negligible | -0.15 (-0.19--0.1) | Negligible | 0.08 (0.06-0.09) | | Negligible | |
| **Poverty of thought** | | 0.03 (-0.04-0.1) | Negligible | -0.13 (-0.18--0.08) | Negligible | -0.14 (-0.18--0.1) | Negligible | 0.08 (0.06-0.1) | | Negligible | |
| **Social withdrawal** | | 0.07 (0-0.14) | Negligible | -0.05 (-0.11-0.01) | Negligible | -0.11 (-0.15--0.07) | Negligible | 0.05 (0.03-0.07) | | Negligible | |
| **Stupor** | | 0.05 (-0.03-0.14) | Negligible | 0.01 (-0.07-0.09) | Negligible | -0.06 (-0.1--0.01) | Negligible | 0.03 (0.01-0.05) | | Negligible | |
| **Suicidality** | | 0.05 (-0.02-0.12) | Negligible | 0.17 (0.1-0.25) | Negligible | 0.13 (0.09-0.17) | Negligible | 0.06 (0.04-0.08) | | Negligible | |
| **Tangential speech** | | 0.15 (0.08-0.23) | Negligible | -0.13 (-0.19--0.07) | Negligible | -0.24 (-0.28--0.19) | Small | 0.13 (0.11-0.14) | | Small | |
| **Tearfulness** | | 0 (-0.07-0.07) | Negligible | 0.2 (0.13-0.28) | Small | 0.23 (0.19-0.26) | Small | 0.1 (0.08-0.11) | | Small | |
| **Thought block** | | 0.08 (0-0.16) | Negligible | -0.13 (-0.18--0.07) | Negligible | -0.18 (-0.22--0.14) | Negligible | 0.1 (0.08-0.12) | | Small | |
| **Thought broadcast** | | 0.1 (0.02-0.18) | Negligible | -0.15 (-0.2--0.09) | Negligible | -0.22 (-0.26--0.17) | Small | 0.12 (0.1-0.14) | | Small | |
| **Thought insertion** | | 0.04 (-0.03-0.11) | Negligible | -0.23 (-0.28--0.18) | Small | -0.24 (-0.29--0.2) | Small | 0.14 (0.12-0.15) | | Small | |
| **Thought withdrawal** | | -0.06 (-0.11--0.02) | Negligible | -0.15 (-0.2--0.1) | Negligible | -0.12 (-0.16--0.07) | Negligible | 0.07 (0.05-0.08) | | Negligible | |
| **Tobacco use** | | 0.15 (0.07-0.22) | Negligible | -0.14 (-0.2--0.08) | Negligible | -0.27 (-0.32--0.23) | Small | 0.14 (0.12-0.16) | | Small | |
| **Waxy flexibility** | | 0 (0-0) | Negligible | -0.02 (-0.07-0.02) | Negligible | -0.02 (-0.07-0.02) | Negligible | 0.01 (0-0.03) | | Negligible | |
| **Weight loss** | | 0.06 (-0.01-0.13) | Negligible | -0.06 (-0.12-0.01) | Negligible | -0.12 (-0.16--0.08) | Negligible | 0.06 (0.04-0.07) | | Negligible | |

# **eReferences**

1. Kotlicka-Antczak M, Podgórski M, Oliver D, Maric NP, Valmaggia L, Fusar-Poli P. Worldwide implementation of clinical services for the prevention of psychosis: The IEPA early intervention in mental health survey. Early Intervention in Psychiatry. 2020;14(6):741–50.

2. Fusar-Poli P. The Clinical High-Risk State for Psychosis (CHR-P), Version II. Schizophrenia Bulletin. 2017 Jan 1;43(1):44–7.

3. Yung AR, Yung AR, Pan Yuen H, Mcgorry PD, Phillips LJ, Kelly D, et al. Mapping the Onset of Psychosis: The Comprehensive Assessment of At-Risk Mental States. Aust N Z J Psychiatry. 2005 Nov 1;39(11–12):964–71.

4. MD TM, PhD BW, MD SW. The Psychosis-Risk Syndrome: Handbook for Diagnosis and Follow-Up. Oxford University Press; 2010. 256 p.

5. Oliver D, Arribas M, Radua J, Salazar de Pablo G, De Micheli A, Spada G, et al. Prognostic accuracy and clinical utility of psychometric instruments for individuals at clinical high-risk of psychosis: a systematic review and meta-analysis. Mol Psychiatry. 2022 Jun 3;1–9.

6. Fusar-Poli P, Borgwardt S, Bechdolf A, Addington J, Riecher-Rössler A, Schultze-Lutter F, et al. The Psychosis High-Risk State. JAMA Psychiatry. 2013 Jan;70(1):107–20.

7. Cupo L, McIlwaine SV, Daneault JG, Malla AK, Iyer SN, Joober R, et al. Timing, Distribution, and Relationship Between Nonpsychotic and Subthreshold Psychotic Symptoms Prior to Emergence of a First Episode of Psychosis. Schizophr Bull. 2021 Apr 29;47(3):604–14.

8. Benasi G, Fava GA, Guidi J. Prodromal Symptoms in Depression: A Systematic Review. PPS. 2021;90(6):365–72.

9. Meter ARV, Burke C, Youngstrom EA, Faedda GL, Correll CU. The Bipolar Prodrome: Meta-Analysis of Symptom Prevalence Prior to Initial or Recurrent Mood Episodes. Journal of the American Academy of Child & Adolescent Psychiatry. 2016 Jul 1;55(7):543–55.

10. Fusar-Poli P, Lai S, Di Forti M, Iacoponi E, Thornicroft G, McGuire P, et al. Early Intervention Services for First Episode of Psychosis in South London and the Maudsley (SLaM): 20 Years of Care and Research for Young People. Front Psychiatry. 2020 Nov 24;11:577110.

11. Stewart R, Soremekun M, Perera G, Broadbent M, Callard F, Denis M, et al. The South London and Maudsley NHS Foundation Trust Biomedical Research Centre (SLAM BRC) case register: development and descriptive data. BMC Psychiatry. 2009 Aug 12;9(1):51.

12. Jackson RG, Patel R, Jayatilleke N, Kolliakou A, Ball M, Gorrell G, et al. Natural language processing to extract symptoms of severe mental illness from clinical text: the Clinical Record Interactive Search Comprehensive Data Extraction (CRIS-CODE) project. BMJ Open. 2017 Jan 1;7(1):e012012.

13. Perera G, Broadbent M, Callard F, Chang CK, Downs J, Dutta R, et al. Cohort profile of the South London and Maudsley NHS Foundation Trust Biomedical Research Centre (SLaM BRC) Case Register: current status and recent enhancement of an Electronic Mental Health Record-derived data resource. BMJ Open. 2016 Mar 1;6(3):e008721.

14. CRIS Natural Language Processing [Internet]. [cited 2022 Jul 13]. Available from: https://www.maudsleybrc.nihr.ac.uk/facilities/clinical-record-interactive-search-cris/cris-natural-language-processing/

15. Fusar-Poli P, Solmi M, Brondino N, Davies C, Chae C, Politi P, et al. Transdiagnostic psychiatry: a systematic review. World Psychiatry. 2019;18(2):192–207.

16. Fusar-Poli P. TRANSD recommendations: improving transdiagnostic research in psychiatry. World Psychiatry. 2019;18(3):361–2.

17. Solmi M, Bodini L, Cocozza S, Seeman MV, Vieta E, Dragioti E, et al. Aripiprazole monotherapy as transdiagnostic intervention for the treatment of mental disorders: An umbrella review according to TRANSD criteria. Eur Neuropsychopharmacol. 2020 Dec;41:16–27.

18. Arango C, Dragioti E, Solmi M, Cortese S, Domschke K, Murray RM, et al. Risk and protective factors for mental disorders beyond genetics: an evidence-based atlas. World Psychiatry. 2021 Oct;20(3):417–36.
